# Flexible transition metal dichalcogenide nanosheets for band-selective photodetection

**DOI:** 10.1038/ncomms9063

**Published:** 2015-09-02

**Authors:** Dhinesh Babu Velusamy, Richard Hahnkee Kim, Soonyoung Cha, June Huh, Reza Khazaeinezhad, Sahar Hosseinzadeh Kassani, Giyoung Song, Suk Man Cho, Sung Hwan Cho, Ihn Hwang, Jinseong Lee, Kyunghwan Oh, Hyunyoug Choi, Cheolmin Park

**Affiliations:** 1 Department of Materials Science and Engineering, Yonsei University, Seoul 120-749, Korea; 2 School of Electrical and Electronic Engineering, Yonsei University, Seoul 120-749, Korea; 3 Department of Chemical and Biological Engineering, Korea University, Anam-dong, Seongbuk-gu, Seoul 136-713, Korea; 4 Photonic Device Physics Laboratory, Institute of Physics and Applied Physics, Yonsei University, Seoul 120-749, Korea

## Abstract

The photocurrent conversions of transition metal dichalcogenide nanosheets are unprecedentedly impressive, making them great candidates for visible range photodetectors. Here we demonstrate a method for fabricating micron-thick, flexible films consisting of a variety of highly separated transition metal dichalcogenide nanosheets for excellent band-selective photodetection. Our method is based on the non-destructive modification of transition metal dichalcogenide sheets with amine-terminated polymers. The universal interaction between amine and transition metal resulted in scalable, stable and high concentration dispersions of a single to a few layers of numerous transition metal dichalcogenides. Our MoSe_2_ and MoS_2_ composites are highly photoconductive even at bending radii as low as 200 μm on illumination of near infrared and visible light, respectively. More interestingly, simple solution mixing of MoSe_2_ and MoS_2_ gives rise to blended composite films in which the photodetection properties were controllable. The MoS_2_/MoSe_2_ (5:5) film showed broad range photodetection suitable for both visible and near infrared spectra.

## Abstract (web summary)

Transition metal dichalgogenide sheets prepared by liquid phase exfoliation can be limited in terms of scalability. Here, Velusamy *et al*. use a scalable liquid phase exfoliation process to fabricate micrometre thick composite nanosheets with amine-terminated polymers, which exhibit photo-detective properties.

## Graphical abstract

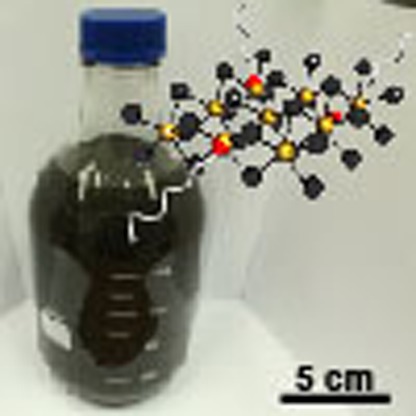

Two-dimensional (2D) nanosheets of transition metal dichalcogenides (TMDs) such as MoS_2_, WS_2_ and MoSe_2_ have been of great attraction due to their intriguing photoelectronic properties associated with the 2D confined chemical structures from insulators, direct bandgap semiconductors to metals^1^,^2^,^3^. These materials have potential for applications in electronics, optics, energy conversion and storage^4^,^5^,^6^,^7^,^8^,^9^,^10^,^11^. In particular, the photocurrent conversion at the characteristic photon energies corresponding to their material-dependent energy band gaps is impressive, making them great candidates for wavelength-selective photodetectors^12^,^13^,^14^,^15^,^16^,^17^,^18^. Excellent visible photodetection has been, for instance, achieved with devices containing single or few layered MoS_2_, as well as WS_2_ fabricated by mechanical exfoliation with Scotch film^19^; convenient but hardly applicable for arrays of the devices.

These promising photoconversion performances of various 2D TMDs is driving a demand for their technological implementation. Furthermore, considering the mechanically flexible nature of the 2D TMDs^20^,^21^, arrays of the photodetectors on either plastic or paper would be beneficial for wearable and patchable applications. Several challenges are, however, ahead for the successful realization. Most importantly, a scalable and universal process suitable for various TMDs irrespective of their detailed chemical structures is required for thin uniform film fabrication on diverse substrates. The method should involve efficient exfoliation of the sheets of 2D TMDs from stacked bulk samples, as well as prevention of re-aggregation of the sheets on film formation. In addition, individual or a few layers of 2D TMDs should be well-connected with each other in the film to readily develop conducting pathways between two electrodes for efficient photocarrier transport.

Many studies were devoted to liquid phase exfoliation and stabilization of TMDs^22^,^23^,^24^,^25^,^26^,^27^,^28^,^29^,^30^,^31^,^32^,^33^ not only because the solvent medium offers an extra driving force for the separation of the sheets but also because 2D TMDs dispersed in solvent can be suitable for various solution-based film processes such as spin-coating, dip-coating and layer-by-layer assembly. To further promote the separation of the sheets, additional interactions with TMDs were required, including ion intercalation^22^, surfactant-driven interaction^29^ and highly boiled medium^33^. Some of the previous approaches are promising and scalable, but limited to specific solvent medium. In addition, photoelectronic properties of mechanically flexible TMD films have not been extensively investigated, in particular, comparable with those with mechanically exfoliated sheets. The non-destructive dispersion strategy with synthetic polymers can be additionally beneficial due to their long and flexible chains that adhere to the surface of TMDs and thus provide sufficient physical gaps between two sheets to mitigate the strong van der Waal interactions of the sheets^34^. We envisioned that end-functionalized polymers can meet the requirements aforementioned with end-functional groups universally interactive with various TMDs. Flexible polymer chains dangled with end-functional moieties firmly anchored on the surface of TMDs in solvents can allow for good dispersion of the sheets with their minimum amount, leading to flexible composite films with many conducting pathways.

Here we present mechanically flexible TMD nanosheets for band-selective photodetection. Our strategy of modifying TMDs with amine-terminated polymers successfully offers a scalable platform suitable for fabricating various flexible TMD/polymer composite films in which few-layer TMD nanosheets are properly separated from each other and embedded in a polymer matrix. The pivotal process for the platform is the extremely efficient liquid exfoliation of TMDs with primary amine-terminated polymers whose end-functional amines are firmly anchored on the surface of TMDs due to strong Lewis acid–base interaction between transition metal and non-pair electrons of amine, allowing us to develop various uniform composite films with numerous combinations of TMD nanosheets and end-functionalized polymers. Films of MoSe_2_ nanosheets with amine-terminated poly(styrene) (PS-NH_2_) exhibited excellent photodetection with an ON/OFF photocurrent ratio of 10^5^, a detectivity of 4 × 10^12^ Jones, a responsivity of 16 A W^−1^ and a response time of 100 ms on 1,064-nm illumination, even when the films were severely deformed at a bending radius of ∼200 μm. Moreover, simple solution mixing of MoSe_2_ and MoS_2_ modified with NH_2_-PS offers an extremely convenient route for tuning the photodetection properties, and thus a blended composite film allows for broad range photodetection from visible to near infrared (NIR). The band-selective photodetection of our blended films is also evidenced by thorough investigation of photo-induced, carrier-relaxation dynamics.

## Results

### Universal exfoliation of TMDs with amine-terminated polymers

**Figure 1:**
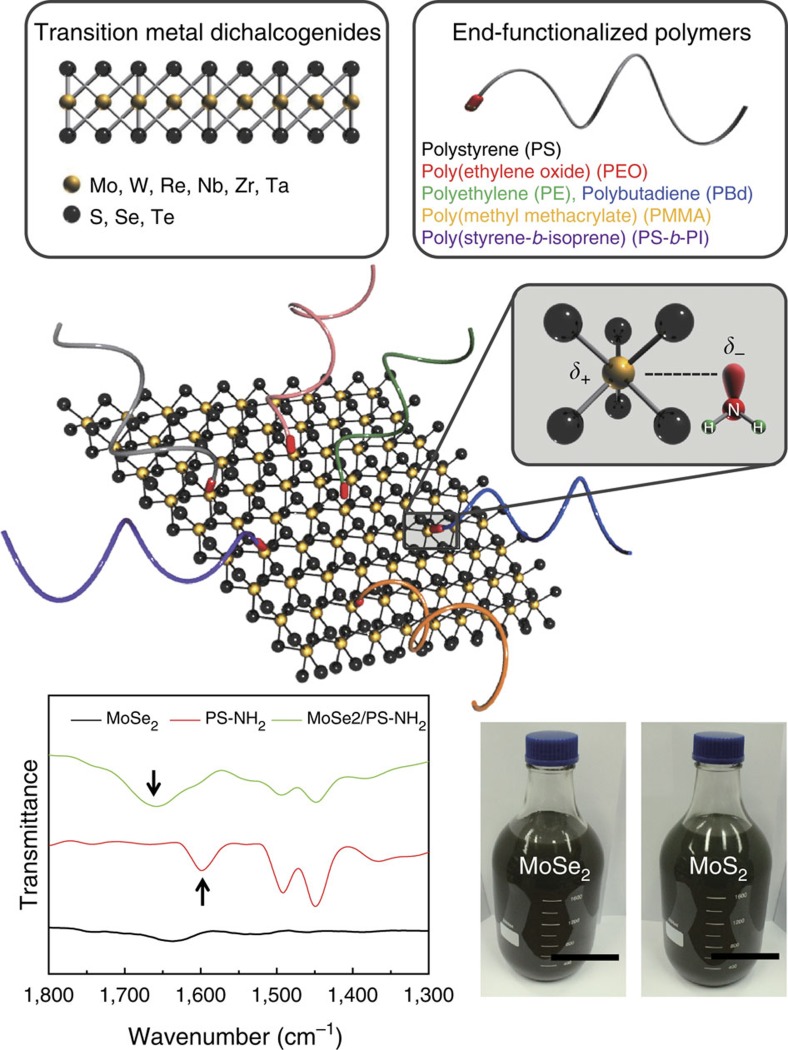
Liquid phase exfoliation of TMDs with end-functionalized polymers. Schematic representation of the proposed mechanism for the exfoliation of various TMDs using different types of amine-terminated end-functionalized polymers in organic solvents. Hexagonal layers of transition metal atoms (M) sandwiched between two layers of chalcogenides (X) are represented by a stoichiometry of MX_2_ by the yellow and grey spheres, respectively. The amine-terminated polymers are simplified for clarity. Interaction between the lone electron pairs of nitrogen atoms and the electron-accepting metal atoms weakens the self layer–layer attraction and the polymer chains provide further separation between the layers, thereby ensuring good exfoliation and dispersion. FT-IR spectra of MoSe_2_, PS-NH_2_ and MoSe_2_-PS-NH_2_ are shown. Few-layer MoSe_2_ and MoS_2_ nanosheets dispersed on the 2-l scale are apparent in the photographs (Scale bar, 5 cm).

In this work, we achieved successful exfoliation of numerous TMD nanosheets such as MoS_2_, WS_2_, MoSe_2_, WSe_2_, ReS_2_, ZrTe_2_ and NbSe_2_ with amine-terminated polymers. The principle is based on the Lewis-like acid–base interaction^22^,^35^ between the transition metal and primary amine, as shown in the scheme in Fig. 1. The representative amine-terminated polymers employed here are glassy PS, poly(methyl methacrylate), rubbery poly(butadiene), semi-crystalline poly(ethylene), poly(ethylene oxide) (PEO) and poly(styrene-*b*-isoprene) copolymer. The interaction between the donated lone pairs of nitrogen atoms and electron-accepting metal atoms was evidenced by Fourier transform infrared spectroscopic (FT-IR) for the case of MoSe_2_ modified with PS-NH_2_. (Supplementary Note 1) The characteristic peak at 1,600 cm^−1^ corresponding to neat NH_2_ was significantly shifted to ∼1,650 cm^−1^, which resulted from the N–H bonds bent due to the transition metal–amine interaction, as shown in Fig. 1. Upshift of N–H mode occurred when primary amines interact with transition metals^36^,^37^. In addition, the computation of IR spectra for model systems (PS-NH_2_, MoSe_2_/PS-NH_2_) based on density functional theory frequency calculation also shows that the peak corresponding to bending vibration of the NH_2_ in the absence of MoSe_2_ was shifted to higher frequency in the MoSe_2_/PS-NH_2_ bound state, which consolidates our experimental observation (Supplementary Fig. 1).

Amine groups of polymers efficiently interact with transition metals when surface of TMD nanosheets is exposed upon sonication step. Simultaneously, flexible polymer chains from the anchored amine on the TMD surface are fully extended in their good solvent medium. Firmly anchored polymer chains on the TMD surface prevent the exfoliated nanosheets from re-aggregating on solvent evaporation. It should be noted that our process does not involve the intercalation of TMD nanosheets, different from ones previously developed with metal ions^22^,^23^,^24^,^25^ (Supplementary Fig. 2; Supplementary Note 1). The successful interaction between amine groups of polymers and transition metals in the TMD nanosheets is confirmed by density functional tight binding^38^,^39^,^40^ and Born–Oppenheimer molecular dynamics simulation (Supplementary Fig. 3). Combined with conventional sonication and centrifugation processes, we were able to exfoliate the bulk TMDs into few-layer nanosheets on a large scale, as shown in the photograph in Fig. 1.

**Figure 2:**
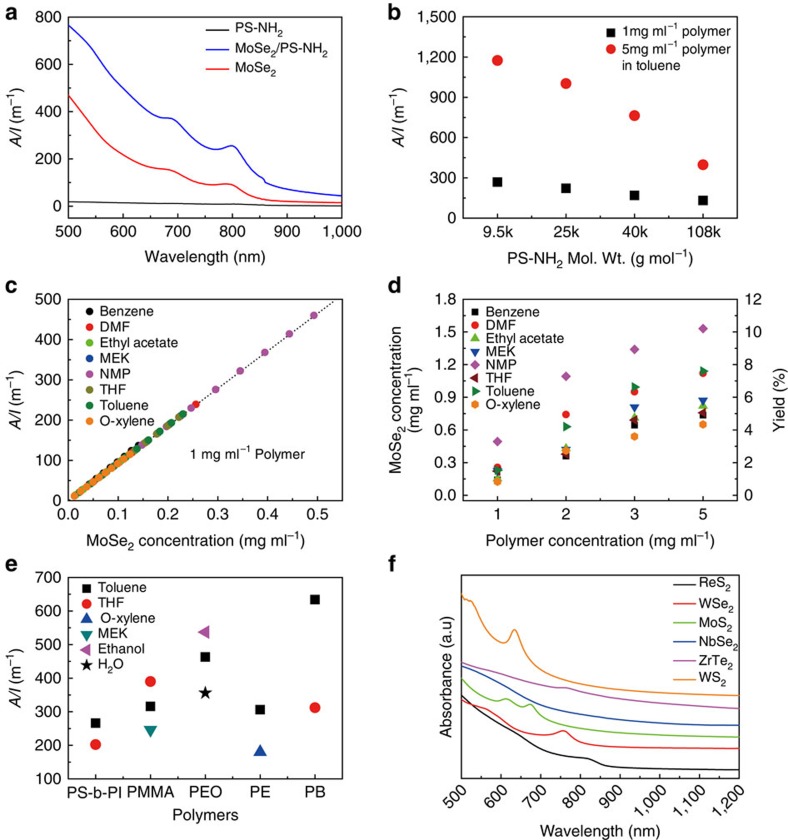
Dispersion characteristics of TMD nanosheets in organic solvents. (**a**) Absorbance spectra of PS-NH_2_ (black), MoSe_2_ dispersed with PS-NH_2_ in toluene (blue) and MoSe_2_ dispersed without PS-NH_2_ in NMP (red). (**b**) Effect of the molecular weight of PS-NH_2_ on the efficiency of the MoSe_2_ dispersion in toluene characterized by the absorbance values of the dispersions at 800 nm. The initial concentrations of MoSe_2_ and PS-NH_2_ were kept constant at 10 mg ml^−1^ and 1 mg ml^−1^, respectively, for all of the molecular weights of PS-NH_2_ evaluated. Increasing the molecular weight of PS-NH_2_ leads to a decrease of the efficiency of the MoSe_2_ dispersion. (**c**) Absorbance at a wavelength of 800 nm as a function of the MoSe_2_ concentration in each solvent. The absorbance linearly increased with the amount of MoSe_2_ following Lambert–Beer behaviour, which implies uniform dispersion of MoSe_2_ without aggregation in all of the solvents. (**d**) Plots of the concentration of MoSe_2_ as a function of the initial concentration of PS-NH_2_ in different organic solvents. Yield of one to three layers (70%) of the exfoliated MoSe_2_ nanosheets with PS-NH_2_ as a function of the initial concentration of PS-NH_2_ in different organic solvents are also shown. (**e**) Absorbance per cell length (proportional to the concentration of dispersed MoSe_2_) of MoSe_2_ dispersed in a range of solvents with a concentration of 3 mg ml^−1^ of the different amine-terminated polymers. Note that PE-NH_2_ was dissolved in hot toluene and xylene. For MoSe_2_ dispersion in water with PEO-NH_2_, sonication was applied for 2 h. (**f**) Absorbance spectra of the dispersions of different TMDs exfoliated in toluene with PS-NH_2_. The absorbance spectra are vertically displaced for clarity.

To demonstrate the effectiveness of our strategy, we extensively investigated the dispersion of MoSe_2_ nanosheets with PS-NH_2_ (Supplementary Note 2). The suspension of bulk MoSe_2_ with 1 mg ml^−1^ PS-NH_2_ in Fig. 2a exhibits the characteristic absorbance spectrum of MoSe_2_ (blue colour) after optimization of the sonication time, initial MoSe_2_ concentration and centrifugation rate (Supplementary Fig. 4). Different from pure PS-NH_2_, discernible peaks were observed at 800 and 690 nm, which are similar to the characteristic peaks of exfoliated MoSe_2_ (red colour) obtained by other methods without PS-NH_2_, confirming the stable exfoliation and dispersion of MoSe_2_ in toluene^26^,^29^,^41^. The increased absorbance at the short wavelength side is attributed to the background scattering arising from TMD nanosheets with 2D anisotropic shape^26^,^29^. To further evidence that the strong absorption peaks in Fig. 2a are attributed to the A or B exciton peaks of MoSe_2_, we performed the absorbance experiments at wavelength-selective system (Supplementary Fig. 5) and the results are the same as Fig. 2a (Supplementary Fig. 6). Additional experiments using X-ray photoelectron spectroscopy, Raman spectroscopy and X-ray diffraction further confirmed the exfoliation and dispersion of MoSe_2_ nanosheets (Supplementary Fig. 7). The characteristic direct bandgap structure of mono and a few layered MoSe_2_ nanosheets was evidenced by photoluminescence (PL) measurement (Supplementary Fig. 8). The PL peak centred at ∼808 nm (1.54±0.01 eV) from A excitons of MoSe_2_ is attributed to an indirect-to-direct bandgap transition, which occurs at the K high symmetry point of the Brillouin zone associated with the quantum confinement in the perpendicular direction^16^,^17^,^42^,^43^,^44^. The PL emission peak from MoSe_2_ modified with PS-NH_2_ is consistent with that from either mechanically exfoliated or CVD synthesized monolayer and the results suggest that the observed PL arises from the intrinsic electronic properties of monolayer MoSe_2_ and our exfoliated MoSe_2_ nanosheets possess semiconducting 2H phase with hexagonal prismatic D_3h_ symmetry. The results we obtained with MoSe_2_ nanosheets modified with PS-NH_2_ were similarly observed with the nanocomposites of MoS_2_ with PS-NH_2_, which confirms the universality of our method (Supplementary Fig. 9).

The effect of the molecular weight of PS-NH_2_ (9.5, 25 and 40 k, and 108 k g mol^−1^) on the exfoliation of MoSe_2_ additionally supports our arguments. For initial polymer concentrations of 1 and 5 mg ml^−1^, the absorbance nearly linearly decreased with increasing molecular weight of polymers due to the decrease of the total number of end-amine groups with increasing molecular weight of the polymers, as shown in Fig. 2b. Based on the results, 9.5 k PS-NH_2_ was further utilized for the exfoliation and dispersions of the TMDs (Supplementary Fig. 10).

A key advantage of our method is the capability of dispersing MoSe_2_ in many solvents. Eight different solvents were examined, which were all good solvents for PS. The results were well-fitted on a single absorbance versus concentration plot of dispersed MoSe_2_, which implies that the MoSe_2_ nanosheets were uniformly dispersed without aggregation, as shown in Fig. 2c (Supplementary Figs 11 and 12). The maximum amount of dispersed MoSe_2_ as a function of the PS-NH_2_ concentration depends on the solvent surface energy and the degree of solubility of PS with solvents^26^,^29^,^30^,^34^ where the results are shown in Fig. 2d. The concentration of dispersed MoSe_2_ increases with increasing PS-NH_2_ concentration but rarely changes when the polymer concentration is 5 mg ml^−1^, indicating the saturation of interaction sites available on the TMD surface. Notably, for all of the solvents examined, at least 0.55 mg ml^−1^ MoSe_2_ was stably dispersed and ∼1.6 mg ml^−1^ MoSe_2_ was successfully exfoliated with our PS-NH_2_ in NMP (Supplementary Fig. 13). The maximum yield percentage of the dispersed MoSe_2_ is >12% in NMP. (Fig. 2d) Compared with previous works^25^,^26^,^27^,^28^,^29^,^30^,^31^,^32^, the maximum amount of dispersed MoSe_2_ is substantially high and, in particular, it should be noted that the scalable exfoliation of MoSe_2_ in various solvents was achieved without re-aggregation for a period of >3 weeks (Supplementary Fig. 14). As proposed in Fig. 1, other amine-terminated polymers also produced MoSe_2_ dispersed in various solvent medium, as shown in Fig. 2e. By choosing PEO, for example, a large amount of MoSe_2_ nanosheets was dispersed in polar media including ethanol and water. To confirm the universality of our method, we dispersed six additional TMDs including MoS_2_, WS_2_, WSe_2_, ReS_2_, ZrTe_2_ and NbSe_2_ with PS-NH_2_ in toluene where their absorbance spectra are shown in Fig. 2f (Supplementary Fig. 15)^29^,^41^.

The microstructures of the exfoliated nanosheets were investigated using both surface probe and electron microscopy, where the results show that the thickness of the single-layer MoSe_2_ was 1.0±0.15 nm, which is thicker than that of pristine MoSe_2_ (ref. 16) (∼0.7 nm) due to the attached polymer chains on both sides of the nanosheet. The single crystalline nature^45^ of the single- and few-layer MoSe_2_ was preserved during the exfoliation process as well (Supplementary Fig. 16). Statistical analysis of both the lateral size and thickness of the exfoliated MoSe_2_ nanosheets (Supplementary Fig. 17) suggests that ∼70% of the MoSe_2_ nanosheets was one to three layers with broadly distributed lateral dimensions ranging from 400 to 800 nm. To demonstrate the ability of controlling the number of TMD layers by our dispersion process with amine-terminated polymers, we varied the centrifuge rate. Statistical analysis of various samples with different centrifuge rates shows that the mean number of layers decreases with the rate and at an optimized condition, a few layered TMDs dispersion was obtained (Supplementary Fig. 17). As expected, bulk and many layers of MoSe_2_ nanosheets prepared by low centrifugation rate do not exhibit any noticeable PL. In addition, electron microscopy results of both bulk and exfoliated TMDs including MoS_2_, WS_2_, WSe_2_, ReS_2_, ZrTe_2_ and NbSe_2_ with PS-NH_2_ in toluene evidence the universality of our method (Supplementary Fig. 18).

### NIR photodetection of flexible MoSe_2_ nanosheets with PS-NH_2_

**Figure 3:**
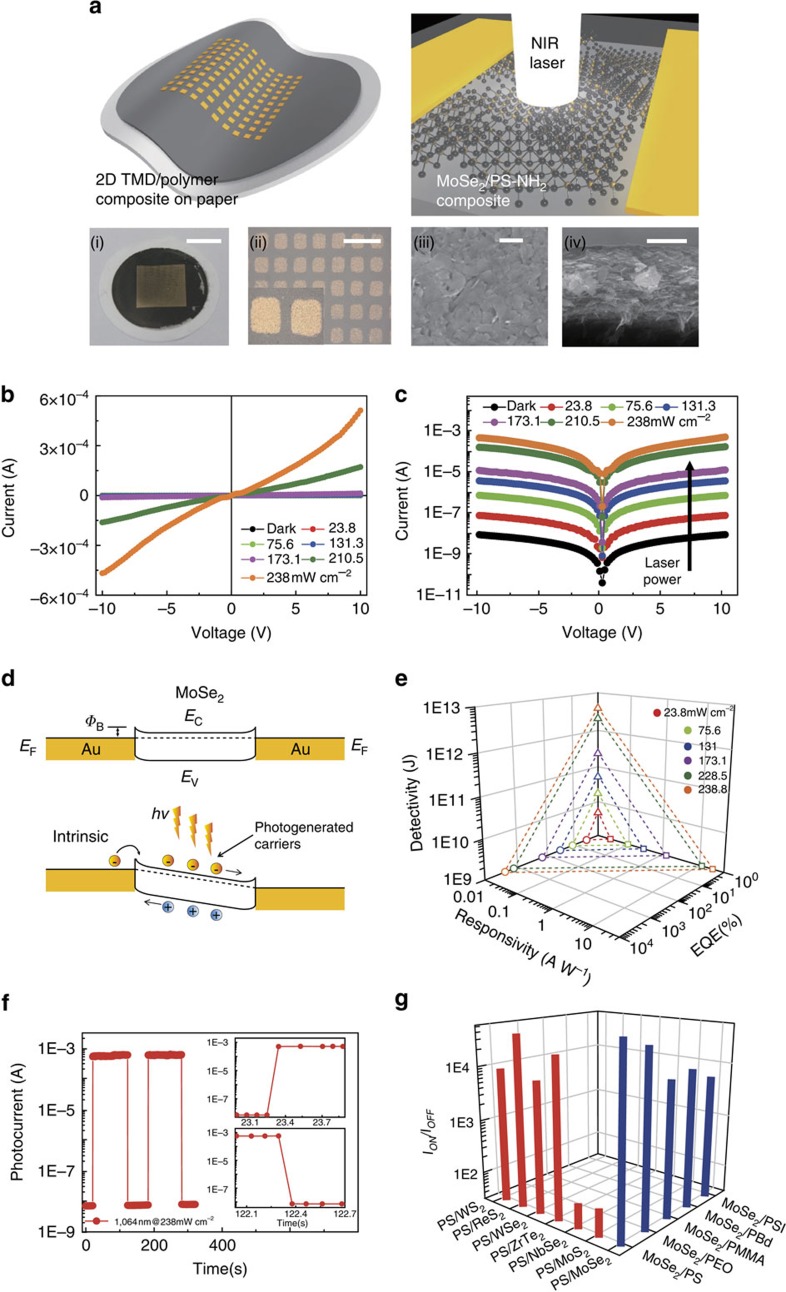
NIR photodetection performance of thin TMD composite films. (**a**) Schematic illustration and photographs of the two-terminal, parallel-type photodetector device cells consisting of MoSe_2_ nanosheets exfoliated with PS-NH_2._ The composite film was deposited on nylon membrane filter paper by a vacuum filtration method. SEM images of the surface and cross-sectional structure of the composite film reveal that few-layer MoSe_2_ nanosheets are stacked with each other with the normal surface of the nanosheets preferentially aligned parallel to the film normal direction. (Scale bars: (i) 6 mm; (ii) 500 μm and (iii and iv) 500 nm) (**b**) Linear and (**c**) semi-log scale *I–V* characteristics of the MoSe_2_ photodetector in the dark and under different light intensities of NIR light at a wavelength of 1,064 nm with a bias voltage of ±10 V. The photocurrent increases with increasing light intensity due to photo-excited carriers in the exfoliated MoSe_2_ nanosheets. (**d**) Band diagram of the MoSe_2_ photodetector device. (**e**) The corresponding responsivity, specific detectivity and external quantum efficiency values of the photodetector as a function of the NIR intensity at a bias voltage of 9 V. (**f**) Photoswitching behaviour of the photodetector under alternating ON and OFF NIR light with an intensity of 238 mW cm^−2^. Both the switch-ON and -OFF times of the detector were ∼100 ms. (**g**) Ratios of the photocurrent to the dark current of the MoSe_2_ composites with different amine-terminated polymers. The current ratios of various TMD composites with PS-NH_2_ are also shown. All of the values were obtained at a light intensity of 238 mW cm^−2^.

To prepare a flexible composite film for use in high performance photodetectors, a suspension of MoSe_2_ nanosheets modified with PS-NH_2_ was carefully poured on a filter paper through which the solvent with non-interacted PS-NH_2_ chains was quickly removed. A micron-thick composite film was developed on the filter paper, as shown in Fig. 3a. The surface and cross-sectional structures of the composite film exhibit that few-layer MoSe_2_ nanosheets were stacked with each other, in which the normal surface of the nanosheets is preferentially aligned parallel to the film normal direction. Subsequent thermal deposition of metal electrodes gave rise to mechanically flexible arrays of two-terminal, parallel-type photodetectors, as schematically depicted in Fig. 3a. On illumination with a NIR laser with a wavelength of 1,064 nm, our composite film became conductive due to photo-excited carriers in the exfoliated MoSe_2_ nanosheets. The photocurrent increases with increasing laser power, yielding a maximum *I*_on_*/I*_off_ ratio of ∼10^5^ at a power density and bias voltage of 238 mW cm^−2^ and 10 V, respectively, as shown in Fig. 3b,c (Supplementary Fig. 19).

There exist mainly three sources of photocurrent arising from TMDs on visible and NIR exposure: (1) photoconduction by photo-induced band excited carriers that can be dissociated into free electrons and holes thermally or by a large electric field; and (2) one by photo-excited carriers decaying into heat that makes TMDs warm, resulting in the reduction of electrical resistance, that is, bolometric photocurrent. (3) Photothermoelectric generation of current by light illumination across metal–TMD interface. In our TMD composite film, we believe that the model by the photo-induced band excitation was dominant while both bolometric and photothermoelectric effect are very marginal (Supplementary Fig. 20). As schematically shown in a band diagram of our two-terminal device of Fig. 3d, the photo-excited carriers in conduction band of MoSe_2_ are drifted to Au electrode with quasi-Ohmic contact under bias field^4^,^18^,^46^,^47^.

The amine-terminated polymer matrix may further promote exciton dissociation on NIR exposure by two possible mechanisms as evidenced in polymer composites with networked semiconducting carbon nanotubes^48^: (1) Exciton separation by thermal energy built up in the insulating polymer around TMDs; and (2) dissociation by enhanced local electric field at the interface of the insulating polymer and TMDs due to the potential barrier formed by the insulating polymer (Supplementary Note 3). The photodetection performance is independent of film thickness ranging from 500 nm to 2 μm, which implies that the laser was able to penetrate through the entire micron-thick film (Supplementary Fig. 21). In addition, no degradation of PS-NH_2_ was observed on NIR and visible laser exposure (Supplementary Fig. 22).

The photocurrent (*I*_ph_) arising from photo-excited photocarriers has in general the relation of *I*_ph_*∝P*^*α*^, and in consequence the current linearly increases with laser power in both log scale^13^. In our system, a similar linear relation was observed in the low power regime but the linearity was deviated at high power >200 mW cm^−2^ with another slope (Supplementary Fig. 19). The rapid increase of photocurrent at high power illumination is known as superlinearity dependence, which has been found in several materials systems but no satisfactory understanding has been made^49^,^50^,^51^,^52^,^53^. Recently, the superlinear photoresponse was also observed in TMD system in which CVD-grown monolayers of chemically alloyed TMDs exhibited very strong superlinearity in particular at high power regimes, which is similar to our results^54^. The superlinear behaviour at high light intensity was explained using a simplified model with three different types of recombination centres in which initially empty and filled intra-gap states exist close to the conduction and valence band, respectively. At increased laser intensity, the occupancy change of these centres arising from shifts in the quasi Fermi levels makes the carrier lifetime longer and in turn the recombination rate slower, leading to superlinear photocurrent.

Both the specific detectivity (D*) and external quantum efficiency values^55^ of our detector increased with increasing laser power up to 4 × 10^12^ Jones and 3 × 10^3^, respectively, as shown in Fig. 3e (Supplementary Note 3). The maximum photoresponsivity of our device was ∼16 A W^−1^ at a power intensity of 238 mW cm^−2^. Notably, our NIR photodetector arrays demonstrated very high cell-to-cell and batch-to-batch reliabilities (Supplementary Fig. 23). The device also exhibited a very fast current switching feature in which a sharp response and decay of photocurrent within ∼100 ms was observed on turn-ON and -OFF of the pulsed laser illumination, as shown in Fig. 3f (Supplementary Fig. 24). In conventional TMD phototransistors (air-exposed TMDs as a channel material), the photo-switching characteristics are primarily determined by the photoconductive effect^56^. In this case, the ON/OFF photocurrent ratio is strongly deteriorated with increasing switching cycle. This is because the air-exposed TMD channel exhibits strong charge-transfer effect between TMDs and the adsorbed H_2_O or O_2_, such that more photo-induced carriers contribute to the charge transfer with decreasing the switching cycle. On the contrary, our photoresponse shows nearly identical ON/OFF photocurrent ratio regardless of the switching cycle (Supplementary Fig. 24). The near zero variation of ON/OFF photocurrent ratio with switching cycle is possibly because the TMD nanosheets are protected by thin polymer composites dispersed in the matrix, that is, TMDs are not exposed to the air. Dark current measured in air was not different from that under vacuum (Supplementary Fig. 24). Similar to the encapsulated TMD devices^57^, the composite amine-terminated polymers effectively prevent from adsorbing H_2_O or O_2_ redox couple, suppressing the charge-transfer effect, with which we are able to achieve stable photodetection operation.

The performance of our MoSe_2_/PS-NH_2_ composite films considering the *I*_on_*/I*_off_ ratio, detectivity and response time is even comparable to the results of previous works of a visible photodetector made of single- or few-layer TMDs. Although the switching shorter than 100 ms were not able to be examined due to the limitation of our switching facility, the switching performance of our composite film is very comparable to that with the monolayer device mechanically cleaved^13^. For the detailed switching dynamics, we provide a zoom-in behaviour in Fig. 3f. Only fast rising transient is observed, implying that the increased photocurrent is largely determined by the photoelectric effect, that is, band-to-band excitation, without the reversible charge transfer from adsorbed molecules to TMD nanosheets. The OFF transient is similar to the ON transient, and comparable to other published results^13^,^16^,^17^,^18^,^46^, suggesting that a fast band-to-band recombination is dominant with absence of the charge-transfer effect.

NIR photodetectors consisting of MoSe_2_ nanosheets were readily fabricated with other amine-terminated polymers such as PEO, poly(methyl methacrylate), PB and PIS, as shown in Fig. 3g (Supplementary Fig. 25). All of the MoSe_2_ composites showed excellent photodetection with a high *I*_on_*/I*_off_ ratio (10^4^) and a detectivity >10^12^, similar to PS-NH_2_. It is also straightforward to develop PS-NH_2_-based composite films containing various TMDs including MoS_2_, WS_2_, ReS_2_, WSe_2_, ZrTe_2_ and NbSe_2_. All of the photodetectors responded on NIR illumination with little variation of the detection performance, which depends on the characteristic photoelectronic properties of the TMDs (Supplementary Fig. 26).

### Band-selective photodetection of mixed TMDs with PS-NH_2_

**Figure 4:**
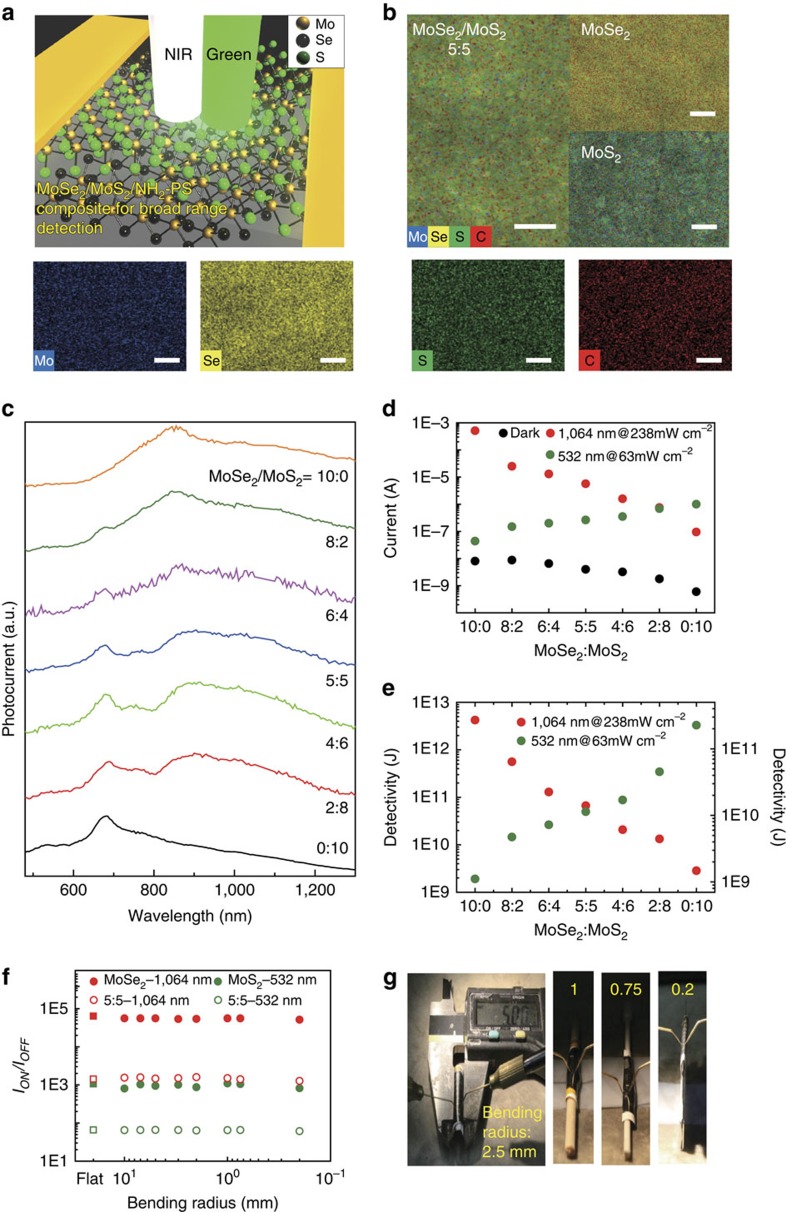
Flexible TMD composite films for band-selective photodetection. (**a**) Schematic illustration of the photodetector containing a mixture of MoSe_2_ and MoS_2_ nanosheets exfoliated with PS-NH_2_. (**b**) Energy dispersive X-ray spectroscopy (EDX) mapping of blended MoS_2_/MoSe_2_ (5:5), MoSe_2_ and MoS_2_ films with PS-NH_2_. Elemental mapping of the individual atoms in the MoS_2_/MoSe_2_ (5:5) film are also showed. (Mo, Blue; Se, Yellow; S, Green; C, Red). The Se and S atoms are uniformly distributed over the composite film, which implies that both MoSe_2_ and MoS_2_ are mixed homogeneously. (In all cases: the scale bar, 2 μm) (**c**) Photoresponse performance of blended composites with different mixing ratio of MoSe_2_ and MoS_2_ with PS-NH_2_ as a function of wavelength in the visible to NIR. (**d**) Photocurrent and (**e**) specific detectivity as functions of the composition of the MoSe_2_ and MoS_2_ mixtures embedded in PS-NH_2_ at wavelengths of 532 nm and 1,064 nm with light intensities of 63 mW cm^−2^ and 230 mW cm^−2^, respectively. (**f**) Ratios of the photocurrent to the dark current of the detectors containing MoSe_2_, MoS_2_ and blended MoSe_2_/MoS_2_ (5:5) composites with PS-NH_2_ as a function of the bending radius. MoSe_2_ and MoS_2_ composites were examined at a wavelength of 1,064 nm with a light intensity of 230 mW cm^−2^ and 532 nm with a light intensity of 63 mW cm^−2^, respectively. On the other hand, blended MoSe_2_/MoS_2_ (5:5) composite film was investigated at both the wavelengths of 1,064 nm and 532 nm with a light intensity of 230 mW cm^−2^ and 63 mW cm^−2^, respectively. (**g**) Photographs of *in situ* measurements of the flexible photodetectors at different bending radii.

More interestingly, our solution process is capable of fabricating composite films with various TMDs in the same polymer matrix, that is, PS-NH_2_, and this method offers a convenient route for band-selective photodetection by simple mixing of two or more different TMDs (Supplementary Note 4). For instance, the excellent photodetection properties of MoSe_2_ and MoS_2_ on illumination at 1,064 nm and 532 nm, respectively, immediately suggest that the performance of a photodetector can be tuned by mixing two TMDs in a PS-NH_2_ matrix (Supplementary Fig. 27). Moreover, our approach allows for a novel photodetector that can detect over a broad optical spectrum range in both visible and NIR regions, as schematically shown in Fig. 4a. Solution-blended composite films of MoSe_2_ and MoS_2_ in a PS-NH_2_ matrix with different compositions showed homogeneous mixing of the two TMDs, as confirmed by energy dispersive X-ray spectroscopy (EDX) in Fig. 4b (Supplementary Figs 28–30).

To demonstrate broadband photoresponse of our blended composites more in detail, we employed a tunable and highly selective laser system (Supplementary Fig. 31) and examined photoresponse performance of blended composites with different mixing ratio of MoS_2_ and MoSe_2_ as a function of wavelength as shown in Fig. 4c. The results clearly show that the wavelength at the maximum photocurrent was controlled by the blend ratio. The band-selective photodetection performance of solution-blended composite films of MoSe_2_ and MoS_2_ in a PS-NH_2_ matrix with different compositions was examined at two representative wavelengths—1,064 and 532 nm. The photocurrent and photodetectivity arising from NIR almost linearly decrease while those from visible light increase with increasing amount of MoS_2_ in the composites, as shown in Fig. 4d,e, respectively (Supplementary Fig. 32). The results clearly indicate that broadband detection is possible, for example, by using a MoS_2_/MoSe_2_ (5:5) composite film. Blended composite films (Supplementary Fig. 33) instantly respond to both 1,064 and 532 nm light at the millisecond level, which is very similar to a MoSe_2_ composite (Fig. 3f).

Our TMD composite photodetector demonstrated high mechanical flexibility. The arrays of photodetectors fabricated on conventional filter paper are readily bendable and photocurrent was detected *in situ* under various bending conditions, as shown in Fig. 4g. Figure 4f shows three representative composite films with PS-NH_2_: neat MoSe_2_, MoS_2_ and blended MoS_2_/MoSe_2_ (5:5). For all three composites, the initial *I*_on_*/I*_off_ ratio values barely changed as a function of the bending radius. The values were still maintained even at a bending radius of ∼200 μm. Our mechanically flexible TMD composite photodetectors are also resistant to multiple and repeated deformation. After 1,000 bending cycles at a bending radius of 1 mm, the devices worked properly without any significant deterioration of performance (Supplementary Fig. 34).

## Discussion

The origin of photocurrent from MoS_2_ and MoSe_2_ nanosheets in composites was further revealed by the behaviour of the photo-induced, carrier-relaxation dynamics of the films. Being strongly quantum confined nature of TMD nanosheets, a well-established fact is that TMDs become a direct-gap semiconductor when the thickness is reduced down to a monolayer limit, and the band-to-band exciton generation/recombination is dominant. At the same time, the increased surface-to-volume ratio leads to fact that the photo-induced exciton recombination suffers enhanced non-radiative recombination, where such changes are strongly dependent of the number of layers. Thus, understanding the photo-induced response of TMD nanosheets and the photodetector performance requires detailed information on the light-induced, time-dependent exciton dynamics. It is known that for 2D TMDs, the non-radiative recombination contains multiple exponential decay components, typically in the sub-nanosecond time scale, which are not easily accessible by conventional electronic measurements. In addition, ultrafast spectroscopy measurements can provide not only qualitative information on the layer-number-dependent non-radiative recombination, but also afford quantitative evaluation on the extracted lifetime. Two sets of ultrafast time-resolved pump–probe spectroscopy measurements were performed: optical-pump and optical-probe (OP–OP) and optical-pump and THz-probe (OP–TP) spectroscopy (Supplementary Fig. 35).

**Figure 5:**
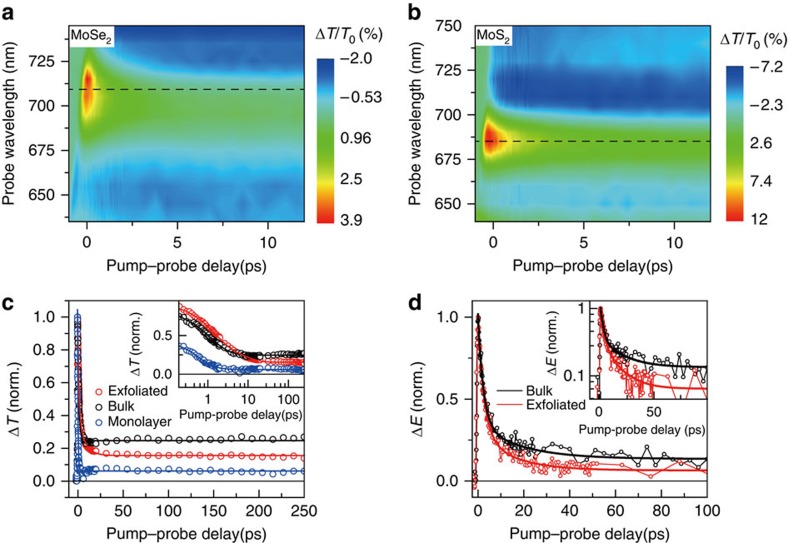
Ultrafast carrier-relaxation dynamics of the TMD composite films. Two-dimensional plots of transient differential transmission Δ*T*/*T*_0_ spectra of the TMD/polymer film. Each plot represents the measurement result—(**a**) MoSe_2_ and (**b**) MoS_2_. (**c**) Time-resolved optical-pump and optical-probe data. Normalized Δ*T* dynamics are shown for polymer-exfoliated MoS_2_ nanosheets (red), bulk (black) and monolayer MoS_2_ (blue). Inset: Δ*T* dynamics plotted with a log scale on *x*-axis. (**d**) Transient dynamics measured by the optical-pump and THz-probe spectroscopy. Pump-induced field change (Δ*E*) of the bulk and polymer-exfoliated MoS_2_ are plotted as a function of the pump–probe delay. Both of the signals were fitted with a bi-exponential decay. Inset: transient THz dynamics plotted on the log scale to emphasize the slow decay dynamics of the exfoliated sample.

To examine the effectiveness of liquid exfoliation of individual TMDs and the corresponding photoresponse of uniformly blended TMDs composite films, we first performed the spectrally and temporally resolved OP–OP measurement on the individual MoS_2_ and MoSe_2_ nanosheets modified with PS-NH_2_, as shown in Fig. 5a,b, and the results are later compared with those from the blended MoS_2_/MoSe_2_ composite (Supplementary Note 5). Here ultrashort 400-nm pump pulse excited the carriers into the continuum, and the broadband white-light pulse was used to probe the spectrally resolved exciton transients. For MoSe_2_ nanosheets, Fig. 5a shows pronounced peak at 710 nm (dashed line), that is, *A*-exciton resonance of MoSe_2_. After a few picosecond decays (∼5 ps), negative signal (Δ*T*/*T*_0_<0) was observed below/above the *A*-exciton resonance. The layer-number-dependent dynamics show clear difference on the carrier-relaxation dynamics between the exfoliated, bulk and monolayer MoSe_2_ as shown in Fig. 5c (Supplementary Fig. 36). Being strongly quantum confined 2D nature of TMD, the large surface-to-volume ratio implies multiple and yet very fast exciton dissociation pathways. On the contrary, the carrier relaxation in bulk typically exhibits much slower responses. Figure 5c shows that our TMD/polymer composite film shows very different carrier-relaxation dynamics compared with the bulk. This corroborates that our TMD nanosheets are well-exfoliated. Similar spectral feature (negative Δ*T*/*T*_0_ around the *A* exciton of MoS_2_ after 5-ps delay) was observed for MoS_2_ nanosheets, as shown in Fig. 5b.

It should be, however, noted that one should be cautious to interpret the optical transient responses since there exist multiple reasons for the observed responses such as exciton linewidth broadening, resonance shift and biexcition formation, together with defect- and Auger-induced recombination. We observed both linewidth broadening and exciton energy shift in our nanocomposite samples but it is not trivial to exactly identify the physical origin of the transient responses from our TMD nanocomposites. For instance, due to the dispersive characters of the MoS_2_ sheet solution exfoliated with PS-NH_2_, inhomogeneous broadening of A and B resonances might occur, giving rise to the energy shift. In addition, the ‘spectral wing' of B exciton overlapped with that of A exciton for the pump-excited non-equilibrium response may result in the energy shift. Under high pump excitation condition, Auger effect may influence our measured signals. In spite of some unambiguity for interpreting the transient responses, the results sufficiently support our claims that our blended MoS_2_/MoSe_2_ composite is well-dispersed individually, rather than just simply mixing two dissimilar TMD powders, corroborating the band-selective photodetection mechanism of Fig. 4.

The THz probe provides a straightforward measure of the photo-induced conductivity dynamics due to the low-energy nature of THz radiation (1 THz=4.136 meV; Supplementary Note 6). In addition, the OP–TP measurements allowed us to examine the validity of liquid polymer exfoliation of TMDs from the bulk, in which the photogenerated THz intraband carrier relaxation is expected to show different conductivity dynamics between the exfoliated TMD-polymer nanosheets and the bulk (Supplementary Figs 37 and 38). In Fig. 5d, we directly compare the THz dynamics of bulk MoS_2_ (black) with the few-layer MoS_2_ nanosheets exfoliated with PS-NH_2_ (red). Immediately after photoexcitation, both the bulk and few-layer MoS_2_ show almost the same rising dynamics. This rapidly rising signal reflects the extremely fast (within a 1-ps THz pulse width) intraband carrier relaxation from the continuum to the bound exciton state. For the long decay component, however, the time scale of the two samples is largely different. The inset in Fig. 5d clearly shows that the MoS_2_-polymer nanosheets possess much faster decay dynamics than that of bulk MoS_2_, presumably due to the interfacial traps arising from the high surface-to-volume ratio of the few-layer MoS_2_ nanosheets^56^,^58^,^59^. It is worth mentioning that the existing THz study^58^ performed similar ultrafast THz experiments and reported the transient decrease of THz conductivity after optical excitation, which is quite different compared with our measurements. There, the negative conductivity dynamics was attributed to the trion formation in highly doped MoS_2_ monolayer. Because the trion can be observed in the high doping case, the possibility of trion formation is low for the weakly doped system. In fact, the mechanically exfoliated or CVD-grown MoS_2_ without any electrostatic doping or chemical doping shows a non-degenerated n-type behaviour, so that it is unlikely that the trion formation is contributed to the THz conductivity dynamics for our case. The results of both the OP–OP and OP–TP spectroscopy analyses suggest not only the effectiveness of our exfoliation process with amine-terminated polymers but also the independent response of MoSe_2_ and MoS_2_ nanosheets in a blended film, which validates our approach for a band-selective detector.

In summary, we demonstrated a scalable platform suitable for fabricating various flexible TMD/polymer composite films in which few-layer TMD nanosheets were properly separated from each other and embedded in a polymer matrix. The pivotal process for the successful platform is the extremely efficient liquid exfoliation of TMDs with primary amine-terminated polymers whose end-functional amines were firmly anchored on the surface of TMDs. More interestingly, our MoSe_2_ and MoS_2_ composites were highly photoconductive even at extreme bending radii as low as 200 μm on illumination of NIR and visible light, respectively. Furthermore, simple solution mixing of MoSe_2_ and MoS_2_ gave rise to blended composite films in which the photodetection properties were tunable. The MoS_2_/MoSe_2_ (5:5) film showed broad range photodetection suitable for both visible and NIR spectra, as confirmed by photo-induced, carrier-relaxation dynamics.

## Methods

### Exfoliation of MoSe_2_ with amine-end-functionalized polymers

TMD powders were purchased from Alfa Aeser and Sigma Aldrich. The amine-terminated polymers and polystyrene homopolymer were procured from Polymer Source Inc., Doval, Canada. The physical properties of the TMD powders and polymers are listed in the Supplementary Tables 1 and 2, respectively. All of the solvents were purchased from Sigma Aldrich. All of the materials were used as received unless otherwise stated. In a typical procedure, 250 mg of bulk MoSe_2_ powder and 25 mg of PS-NH_2_ were added into a 30-ml glass vial containing 25 ml of toluene. The solution was sonicated for 45 min by using a tip sonicator with a 10-s On pulse and a 5-s OFF pulse at an amplitude of 50% in an ice bath. The dispersions were allowed to settle for 24 h, and then the top dispersion was decanted and centrifuged for 30 min at 1,500 r.p.m. to remove the unexfoliated and large particles. After centrifugation, the top half of the dispersion was collected and the concentration of MoSe_2_ nanosheets was determined by standard gravimetric analysis.

### Photodetector fabrication

To fabricate the devices, stock solutions were centrifuged for 90 min at 15,000 r.p.m. to remove the excess non-interacted polymers in the dispersions. Then, the precipitate was collected and dried to remove the solvents. The resultant powder consisted of ∼8:2 (w/w) ratio of MoSe_2_ and PS-NH_2_. The desired amount of powder was dispersed in the solvent by bath sonication for 20 min and then filtered under vacuum onto a nylon membrane filter paper with a pore size of 200 nm and a diameter of 25 mm. For the fabrication of the mixture of the TMD composite films, first, powder of the individual TMD nanosheets with polymers was dispersed in the solvent. Then, the solutions were mixed together at different weight ratios by a simple solution blending method and deposited on filter paper. The resulting film was dried in a vacuum oven at 60 °C for 4 h. Parallel Au electrodes with thicknesses of 50 nm were deposited onto a MoSe_2_ film by thermal evaporation under a vacuum of 10^−6^ torr using a patterned shadow mask. The length and width of the channels were 50 μm and 200 μm, respectively.

### Equipment and characterization

A horn probe tip sonicator (VibraCell CVX; 750 W) was used to exfoliate the TMDs with amine-terminated polymers. Centrifugation was carried out using a Hettich Mikro 22R centrifuge. The optical absorbance spectra were measured by a ultraviolet–visible–NIR spectrophotometer (JASCO V-530) using 1-cm quartz cuvettes. FT-IR studies were performed using a JASCO FT-IR 300E apparatus (Tokyo, Japan) with KBr as a standard. The thermogravimetric analysis (TGA) of the samples was carried out using a TA Q500 thermal analyser at a heating rate of 10 °C min^−1^ under a nitrogen atmosphere. X-ray photoelectron spectroscopy (K-alpha Thermo VG, .K.) measurements were acquired using a monochromated Al X-ray source (Al Kα line: 1486.6 eV). Raman and PL measurements (LabRamAramis) were carried out using a 532-nm laser at a power of 0.5 mW and an exposition time of 10 s. X-ray diffraction patterns were recorded using a Dmax/2500-H (Rigaku, Japan). The nanostructures of the TMDs were examined by tapping mode atomic force microscopy (Nanoscope IV Digital Instruments) in the height and phase contrast mode, field emission scanning electron microscopy (FESEM, JEOL JSM-7001F) with an acceleration voltage of 10 kV and high resolution transmission electron microscopy (HRTEM, JEOL 2100F) at 200 kV in the bright field. TEM samples were prepared by drop casting the dispersions on a holy carbon grid, followed by drying under vacuum for 24 h at 50 °C. Selected area electron diffraction patterns were obtained in the HRTEM analysis. Energy dispersive X-ray (EDX) spectra of the TMD/polymer composite films were obtained using an EDX spectrometer by FESEM with an acceleration voltage of 10 kV. The electrical properties of all devices were measured at room temperature in air with a Hewlett-Packard 4145B semiconductor parameter analyser. The 532 nm and 1,064 nm wavelength light were generated from different continuous-wave semiconductor diode laser sources, Shanghai dream Lasers Technology Co., Ltd., Model number: SDL-532-005T and Qbic Laser system, Model number: QBFDL-1064-150-1000, respectively. A laser power meter was used to measure the incident power of the laser pulses. The exciton formation and recombination kinetics of the MoSe_2_ and MoS_2_ composite film with PS-NH_2_ was measured with ultrafast optical-pump and optical-probe spectroscopy. The photogenerated intraband carrier-relaxation dynamics of few-layer MoS_2_ nanosheets exfoliated by PS-NH_2_ was examined with ultrafast OP–TP spectroscopy. The detailed experimental set-up for the ultrafast spectroscopy measurements are shown in Supplementary Figs 35 and 37.

## Additional information

**How to cite this article:** Velusamy, D. B. *et al*. Flexible transition metal dichalcogenide nanosheets for band-selective photodetection. *Nat. Commun*. 6:8063 doi: 10.1038/ncomms9063 (2015).

## Supplementary Material

Supplementary Information Supplementary Figures 1-38, Supplementary Tables 1-2, Supplementary Notes 1-6 and Supplementary References.
