# Supplementary material for: Flexible transition metal dichalcogenide nanosheets for band-selective photodetection
Source: Nat Commun. 2015 Sep 2;6:8063. doi: 10.1038/ncomms9063 (PMC4569699; doi:10.1038/ncomms9063)
Supplement: Supplementary Information — Supplementary Figures 1-38, Supplementary Tables 1-2, Supplementary Notes 1-6 and Supplementary References. [file ncomms9063-s1.pdf]

SUPPLEMENTARY INFORMATION

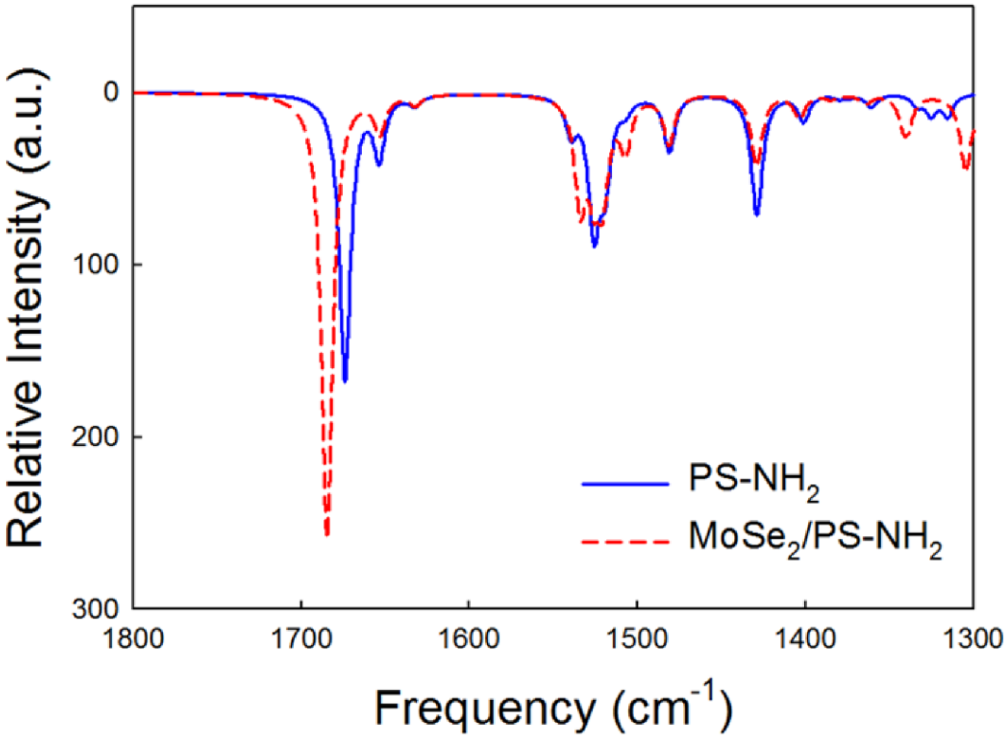

Supplementary Figure 1. IR simulation spectra of interaction between polymers and TMDs.

Comparison of the simulated IR spectra of PS-NH_2 (Blue line) and $MoSe_2$/PS-NH_2 (dotted red line).

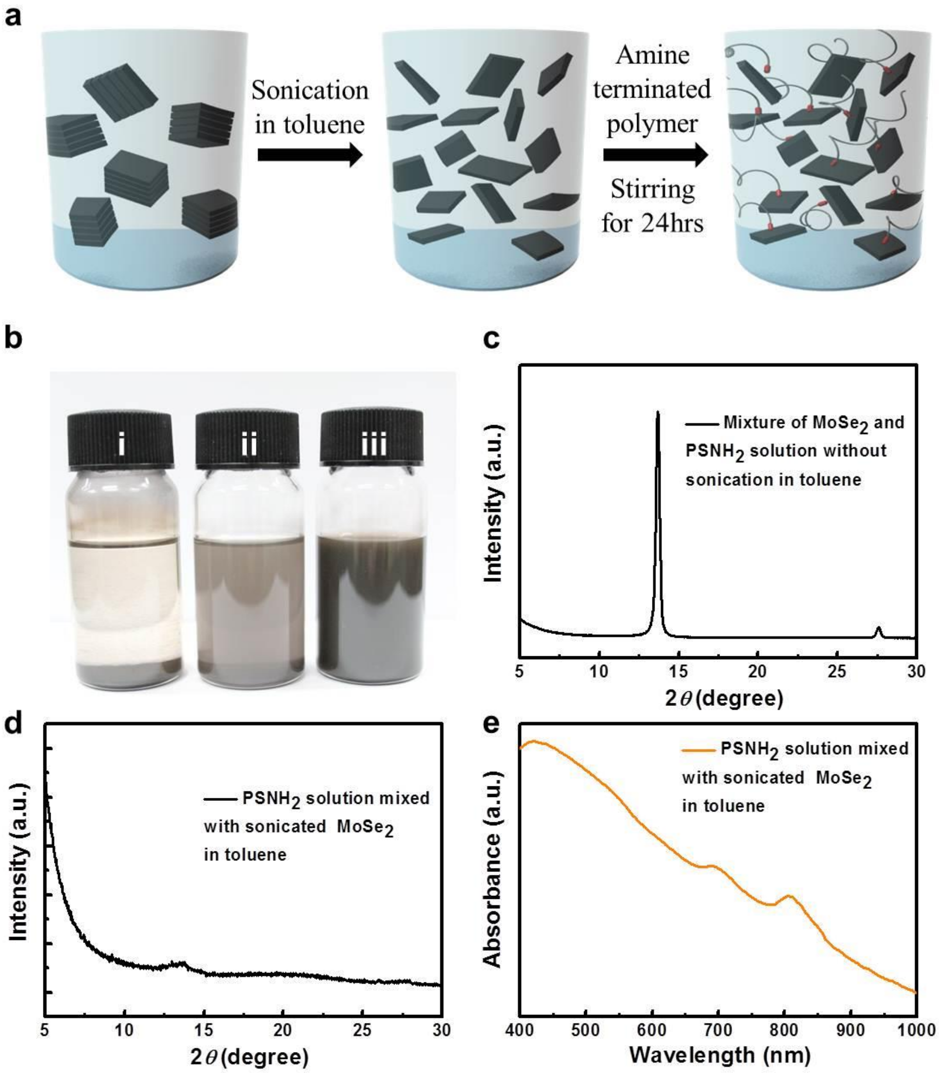

Supplementary Figure 2. **Exfoliation and dispersion of TMDs with end-functionalized polymers. a**, Schematic illustration of the proposed mechanism for the exfoliation and dispersion of various TMDs using different types of amine-terminated end-functionalized polymers. A solution of bulk $MoSe_2$ in toluene was sonicated without the addition of PS-NH_2 and subsequently mixed with PS-NH_2 solution (sonicated and mixed solution). Compared to $MoSe_2$ solution sonicated in toluene without polymer and simple mixing of bulk $MoSe_2$ and PS-NH_2 in toluene, which were immediately settled down in few minutes, the sonicated and mixed solution exhibited $MoSe_2$ well dispersed in toluene. **b**, Photograph of (**i**) bulk $MoSe_2$ in toluene was sonicated without the addition of PS-NH_2 (**ii**) simple mixing of bulk $MoSe_2$ and PS-NH_2 in toluene (**iii**) bulk $MoSe_2$ in toluene was sonicated and subsequently mixed with PS-NH_2 solution. XRD results of **c**, Mixture of bulk $MoSe_2$ and PS-NH_2 in toluene without sonication and **d**, bulk $MoSe_2$ in toluene was sonicated and subsequently mixed with PS-NH_2 solution. **e**, Absorbance spectra of bulk $MoSe_2$ in toluene was sonicated and subsequently mixed with PS-NH_2 solution.

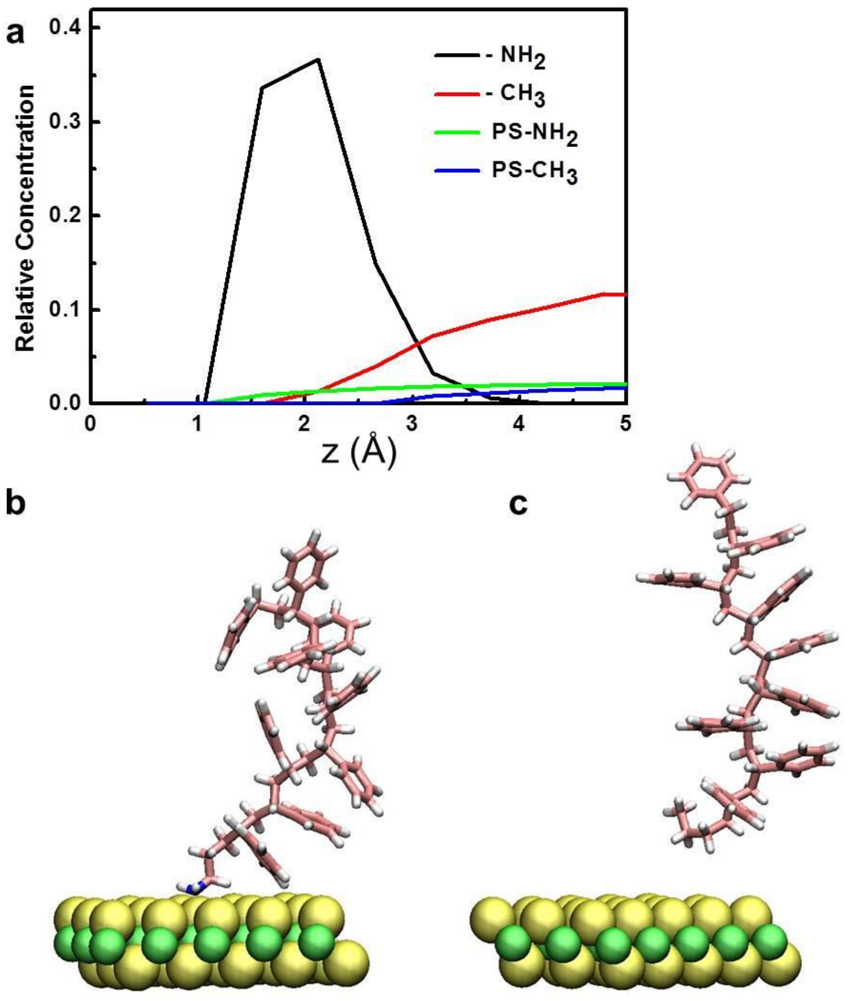

Supplementary Figure 3. **Simulation results of interaction between polymers and TMDs. a,** Comparison of the simulated concentration profile of -NH_2,-CH_3, PS-NH_2 and PS-CH_3 in the direction perpendicular to the TMD surface (z). Simulation snapshot of typical conformational states for **b,** PS-NH_2 and **c,** PS-CH_3 on the TMD surface.

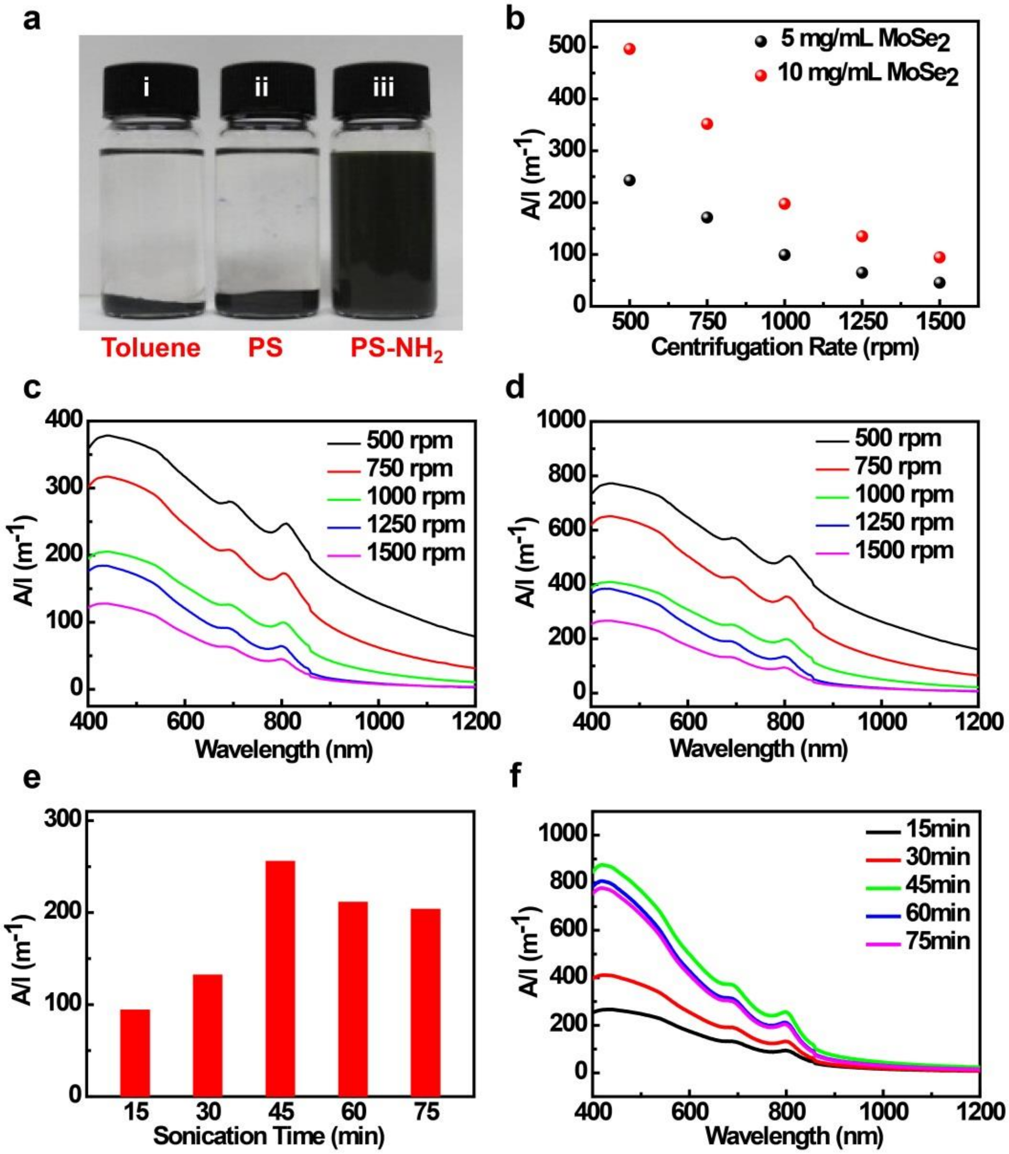

Supplementary Figure 4. **Optimization of the $MoSe_2$ dispersions with PS-NH_2**. **a**, Photographs of the $MoSe_2$ dispersion in toluene (i) without any dispersant, (ii) with PS, and (iii) with PS-NH_2. **b**, Effect of the centrifugation rate on the $MoSe_2$ dispersion. Absorbance spectra of two different initial $MoSe_2$ concentrations were evaluated: **c**, 5 mg/mL and **d**, 10 mg/mL. Sonication time was 15 min. **e**, Effect of the sonication time and **f**, the corresponding absorbance spectra of $MoSe_2$ dispersion in toluene. For all of the sonication times evaluated, the initial concentrations of $MoSe_2$ and PS-NH_2 were kept constant at 10 and 1 mg/mL, respectively, and the solutions were centrifuged at 1,500 rpm for 30 min.

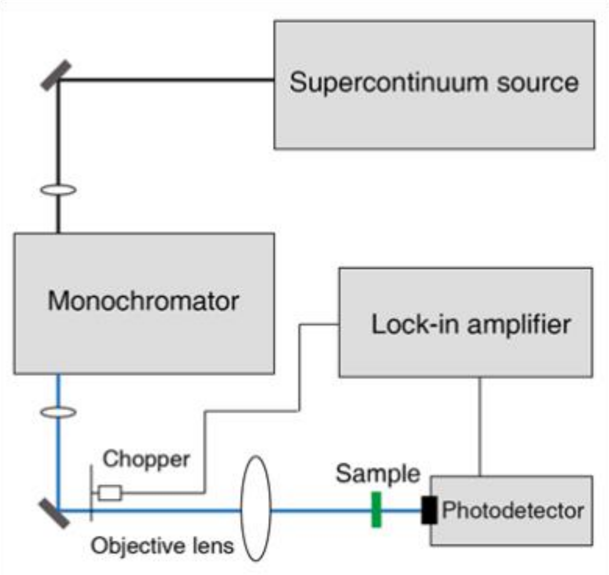

Supplementary Figure 5. **Absorption measurement with high selectivity system.** Schematic of the absorption spectrum measurements.

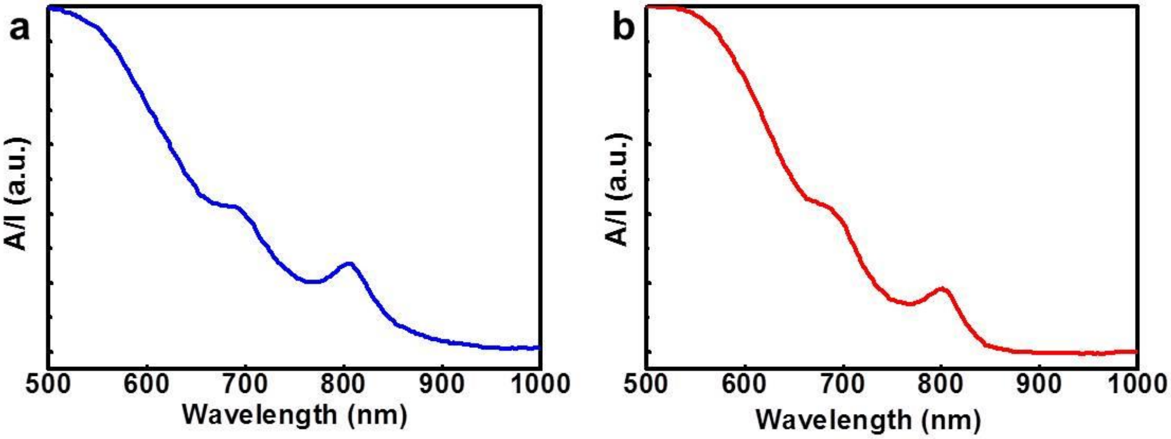

Supplementary Figure 6. **Absorption spectra of $MoSe_2$ with and without PS-NH_2.** Absorption spectra of **a**,$MoSe_2$ dispersed with PS-NH_2, and **b**,$MoSe_2$ dispersed without PS-NH_2.

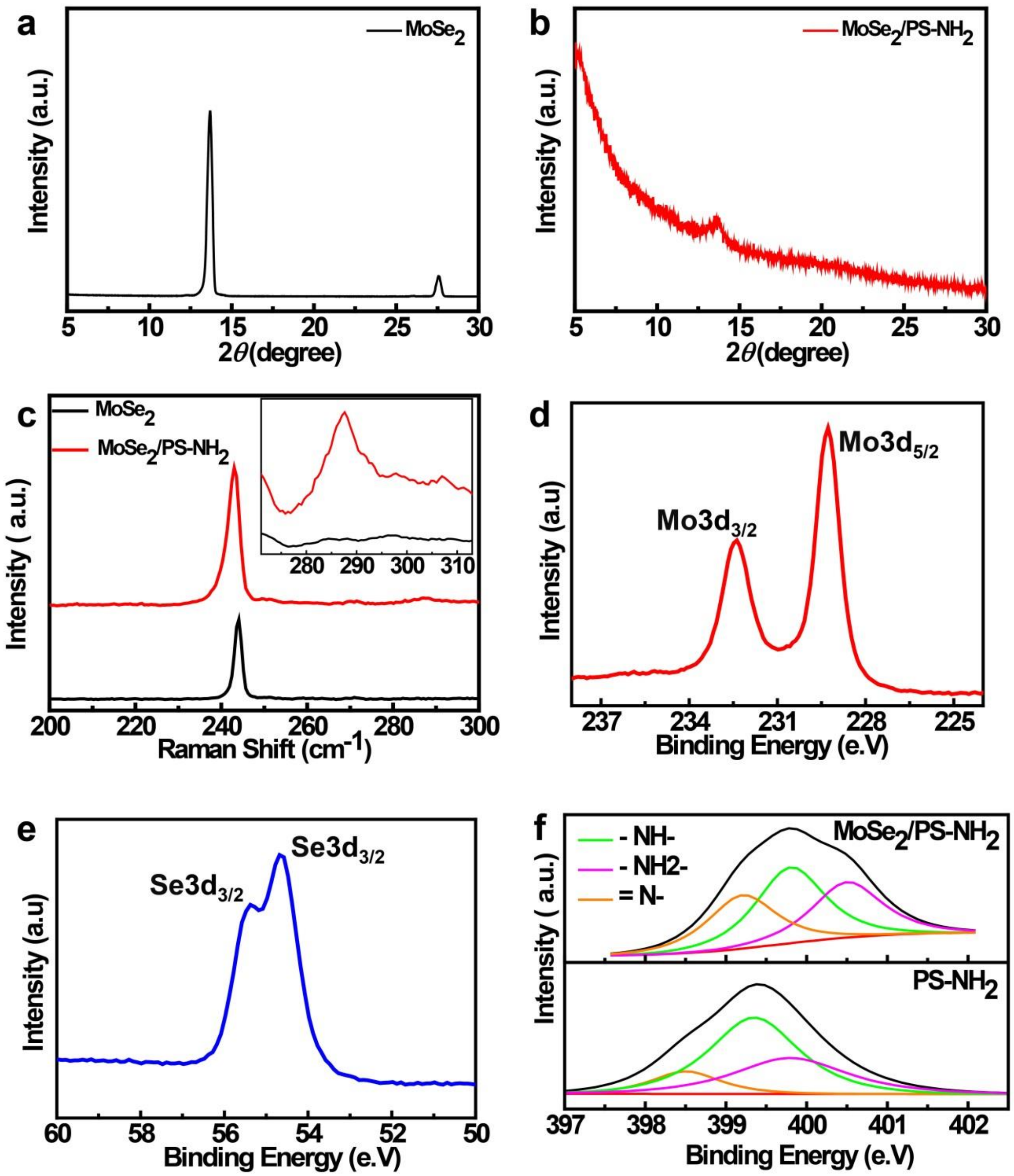

Supplementary Figure 7. **Characterization of the exfoliated $MoSe_2$ nanosheets with PS-NH_2**. XRD of **a**, bulk $MoSe_2$ and **b**, exfoliated $MoSe_2$ nanosheets with PS-NH_2. **c**, Raman spectra of bulk $MoSe_2$ and $MoSe_2$/PS-NH_2. XPS of the $MoSe_2$ nanosheets exfoliated with PS-NH_2. **d**, Mo 3d spectrum **e**, Se 3d Spectrum **f**, N 1s spectra of PS-NH_2 and $MoSe_2$/ PS-NH_2.

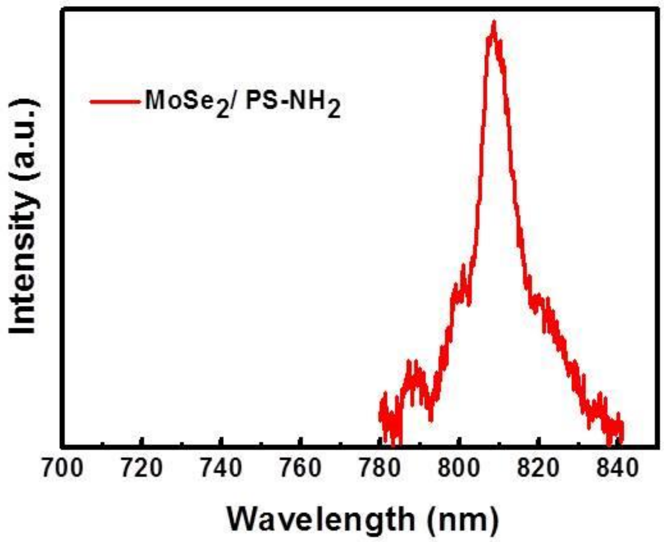

Supplementary Figure 8. **Optical properties of the exfoliated $MoSe_2$ with PS-NH_2**. Photoluminescence (PL) spectrum of $MoSe_2$/PS-NH_2 in toluene spin coated on the glass substrate with an excitation wavelength of 532 nm. The PL Spectrum of exfoliated $MoSe_2$/PS-NH_2 confirm the indirect-to–direct band gap transition of $MoSe_2$,which implies the single layer and semiconductor nature of the exfoliated $MoSe_2$ prepared in our method.

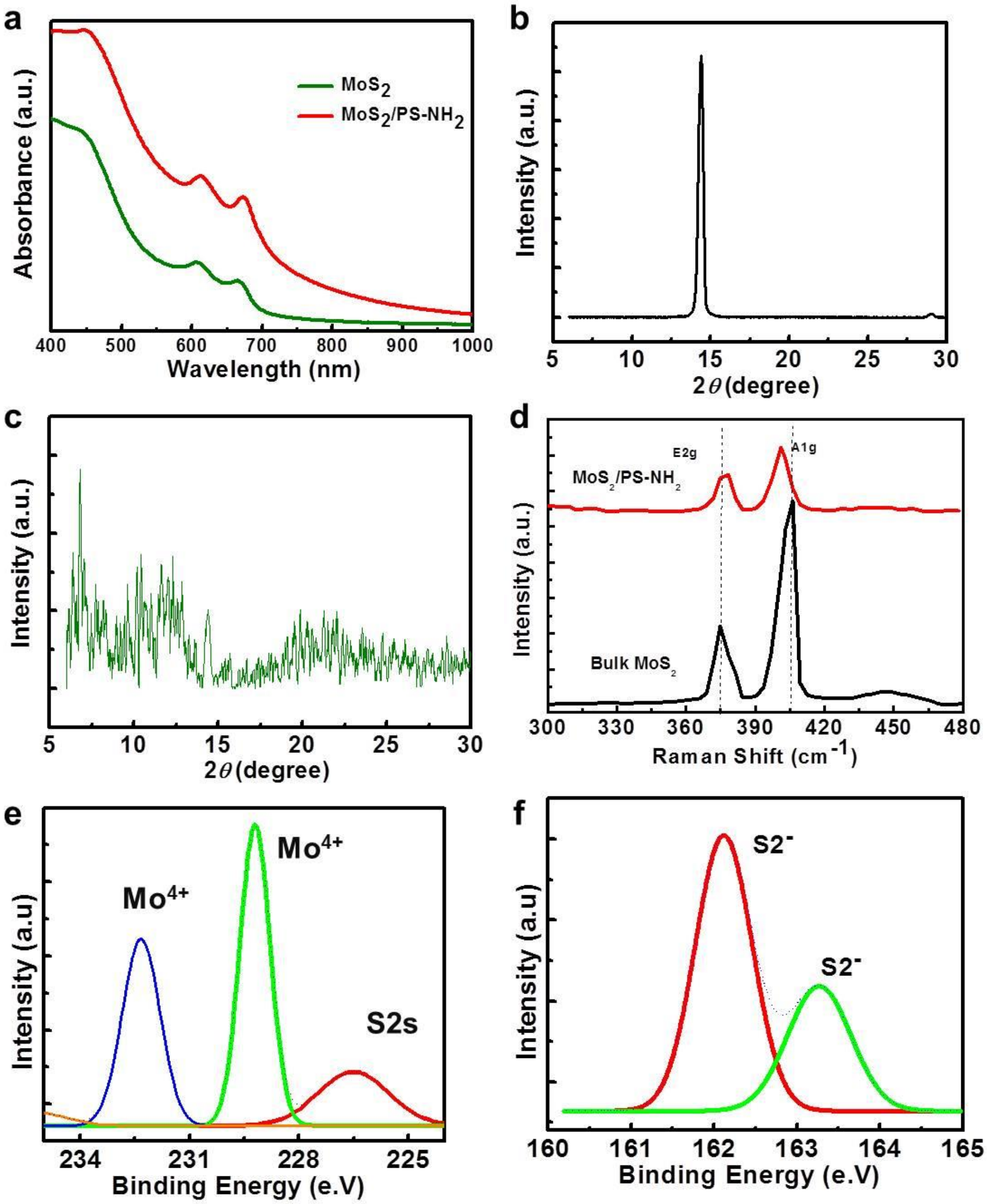

Supplementary Figure 9. **Characterization of the exfoliated MoS_2 nanosheets with PS-NH_2**. **a**, Absorbance spectra of MoS_2 dispersed with PS-NH_2 in toluene (red) and MoS_2 dispersed without PS-NH_2 in NMP (green).XRD of **b**, bulk MoS_2 and **c**, exfoliated MoS_2 nanosheets with PS-NH_2. **d**, Raman spectra of bulk MoS_2 and MoS_2/PS-NH_2. XPS of the MoS_2 nanosheets exfoliated with PS-NH_2. **e**, Mo 3d spectrum and **f**, S 3d Spectrum. The obtained results of MoS_2 nanosheets modified with PS-NH_2 were similar to the observed nanocomposites of $MoSe_2$ with PS-NH_2.

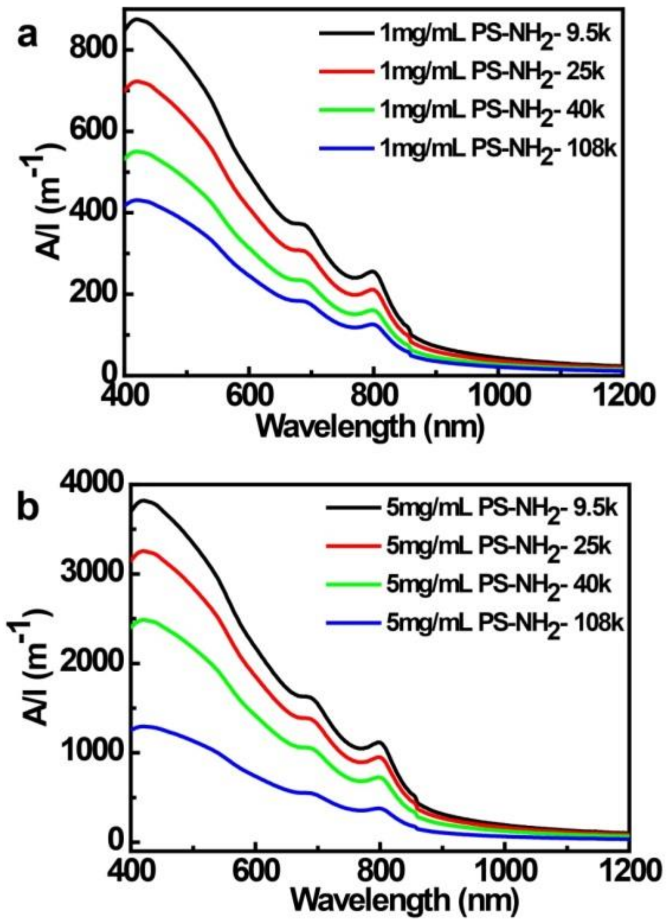

Supplementary Figure 10. **Effect of the PS-NH$_2$ molecular weight on the MoSe$_2$ dispersion.** Absorbance spectra of MoSe$_2$ dispersions depending on PS-NH$_2$ with different molecular weights for initial polymer concentrations of **a**, 1 mg/mL and **b**, 5 mg/mL.

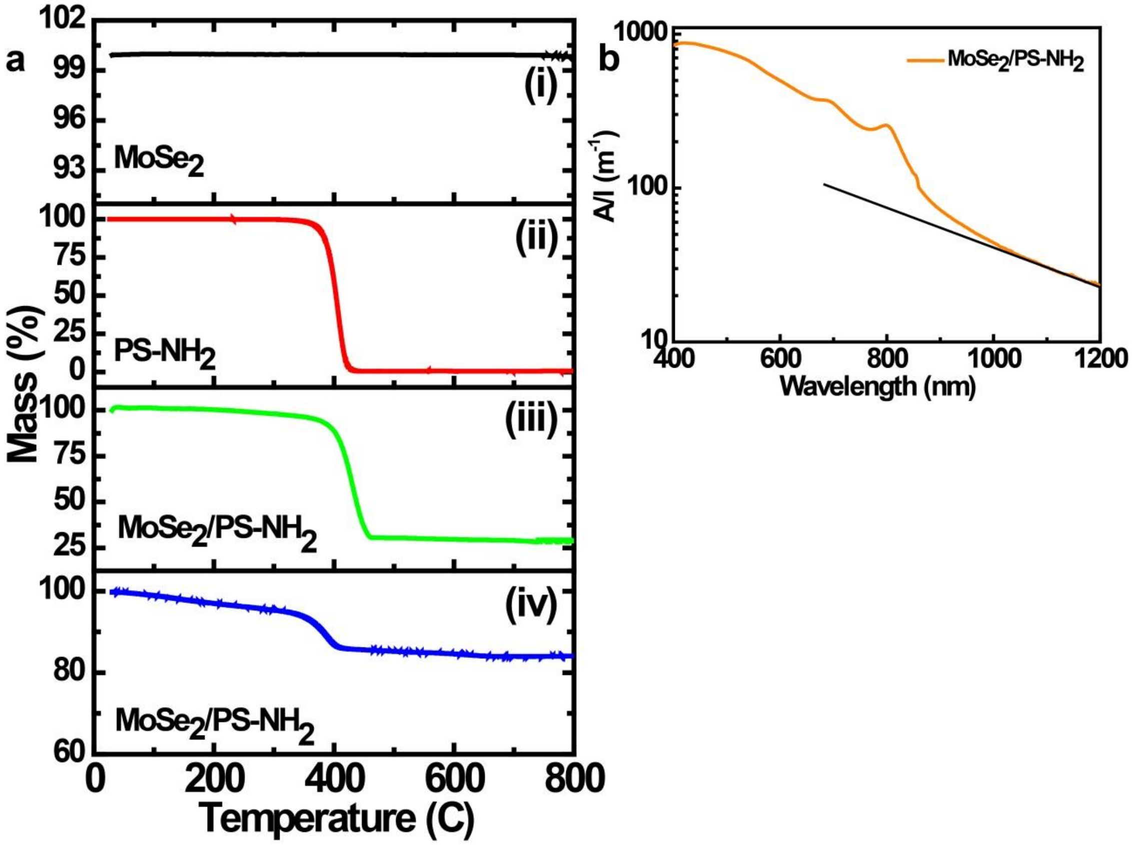

Supplementary Figure 11. **Determination of the $MoSe_2$ concentration. a**, Thermogravimetric analysis for (i) the $MoSe_2$ powder, (ii) PS-NH_2, (iii) $MoSe_2$ modified with PS-NH_2, and (iv) after removing the excess PS-NH_2 at 15,000 rpm for 90 min in the $MoSe_2$ dispersion. **b**, Absorbance spectrum of the $MoSe_2$ dispersion with 1 mg/mL PS-NH_2 on the log scale.

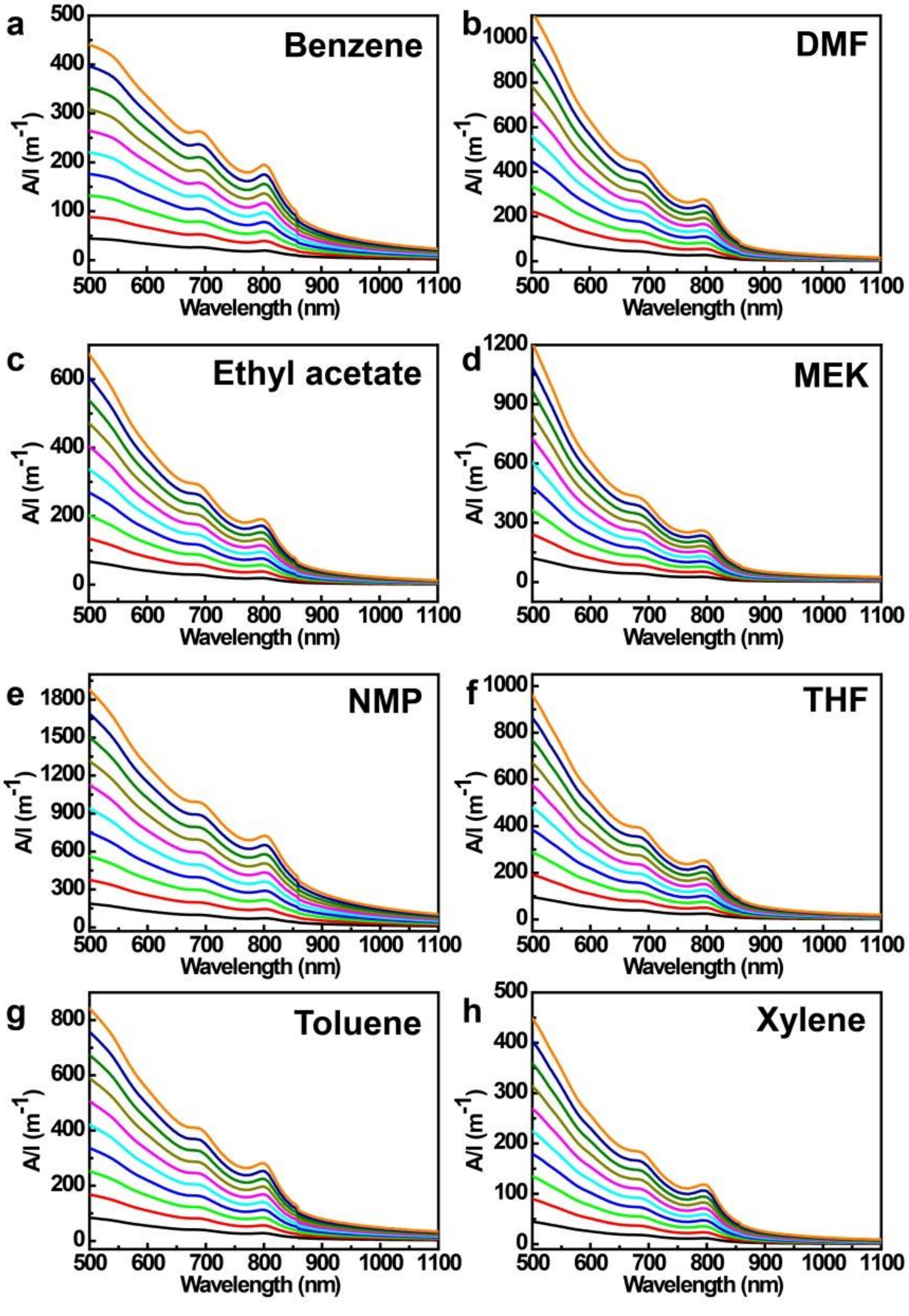

Supplementary Figure 12. **$MoSe_2$ dispersion in various solvents.** Absorbance spectra of the series of diluted $MoSe_2$ dispersions with 1 mg/mL PS-NH_2 in various solvents.

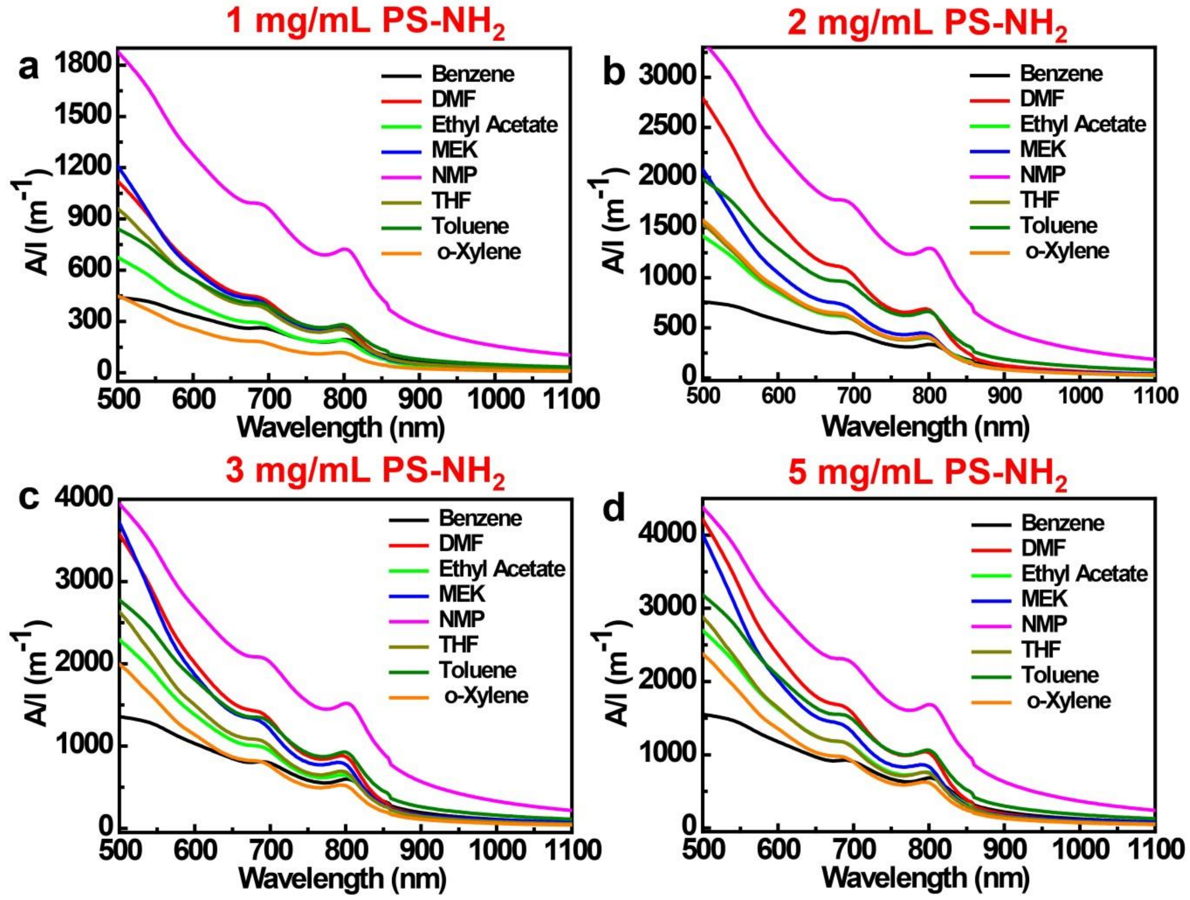

Supplementary Figure 13. **$MoSe_2$ dispersions with different PS-NH_2 concentrations.** Absorbance spectra of $MoSe_2$ dispersions in various solvents as a function of the PS-NH_2 concentration.

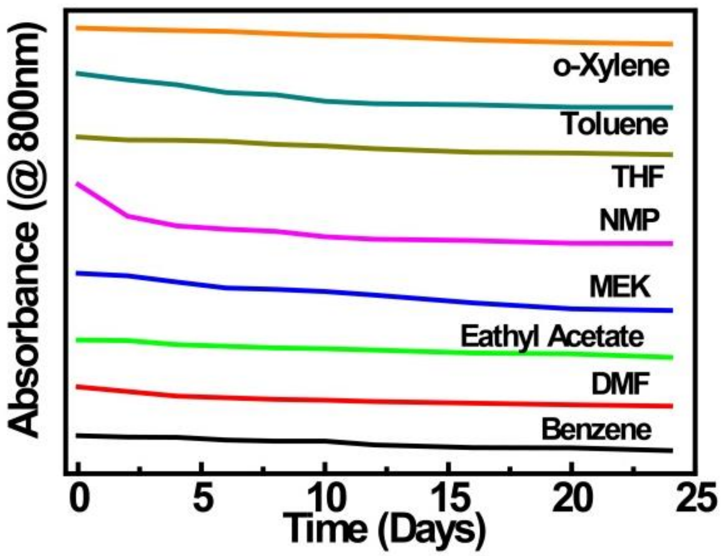

Supplementary Figure 14. **Stability of dispersed $MoSe_2$.** Absorbance of $MoSe_2$ dispersions measured at 800 nm in various solvents with 5 mg/mL PS-NH_2 as a function of time. The absorbance results are vertically shifted for clarity.

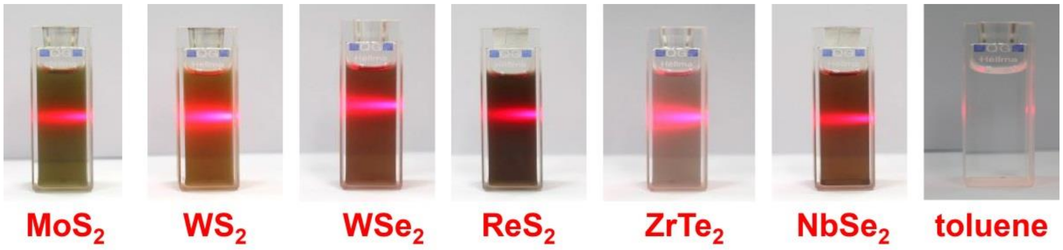

Supplementary Figure 15. **Exfoliation of the different TMDs with PS-NH_2.** Photographs of different TMDs dispersed in toluene with 1 mg/mL of PS-NH_2. All of the dispersions were diluted to observe variations of the color. A red laser beam was directed through the TMD dispersions to show the Tyndall effect of the colloidal solutions. For comparison, pure toluene is also shown.

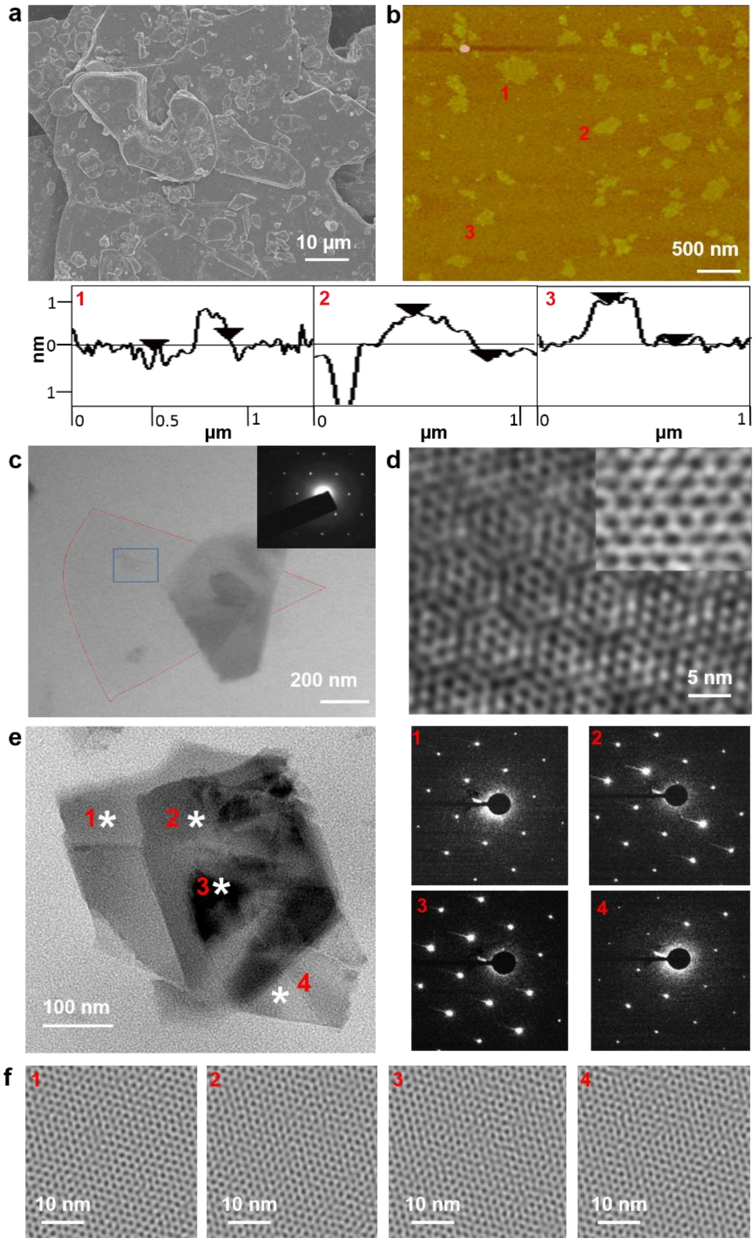

Supplementary Figure 16. **Morphology of the $MoSe_2$ nanosheets. a**, SEM image of bulk $MoSe_2$ powder. **b**, Tapping mode AFM micrograph along with height profile of distribution of exfoliated $MoSe_2$ nanosheets in toluene spin-coated on a Si substrate. The height profile measured in clearly shows a monolayer $MoSe_2$ sheet with a thickness of approximately 1.0 nm. **c**, Bright field TEM image of single- and few-layer $MoSe_2$ nanosheets. The inset shows the selected area electron diffraction (SAED) pattern of a $MoSe_2$ nanosheet. **d**, High resolution TEM (HR-TEM) image of the $MoSe_2$ nanosheets. The digitally filtered image in the inset clearly shows hexagonal symmetry of the $MoSe_2$ nanosheets. The SAED pattern and HR-TEM collected from the marked box region. **e**, Bright field TEM image of few-layer $MoSe_2$ nanosheets and different thickness regions marked with numbers and their corresponding SAED patterns. **f**,HR-TEM images of different regions.

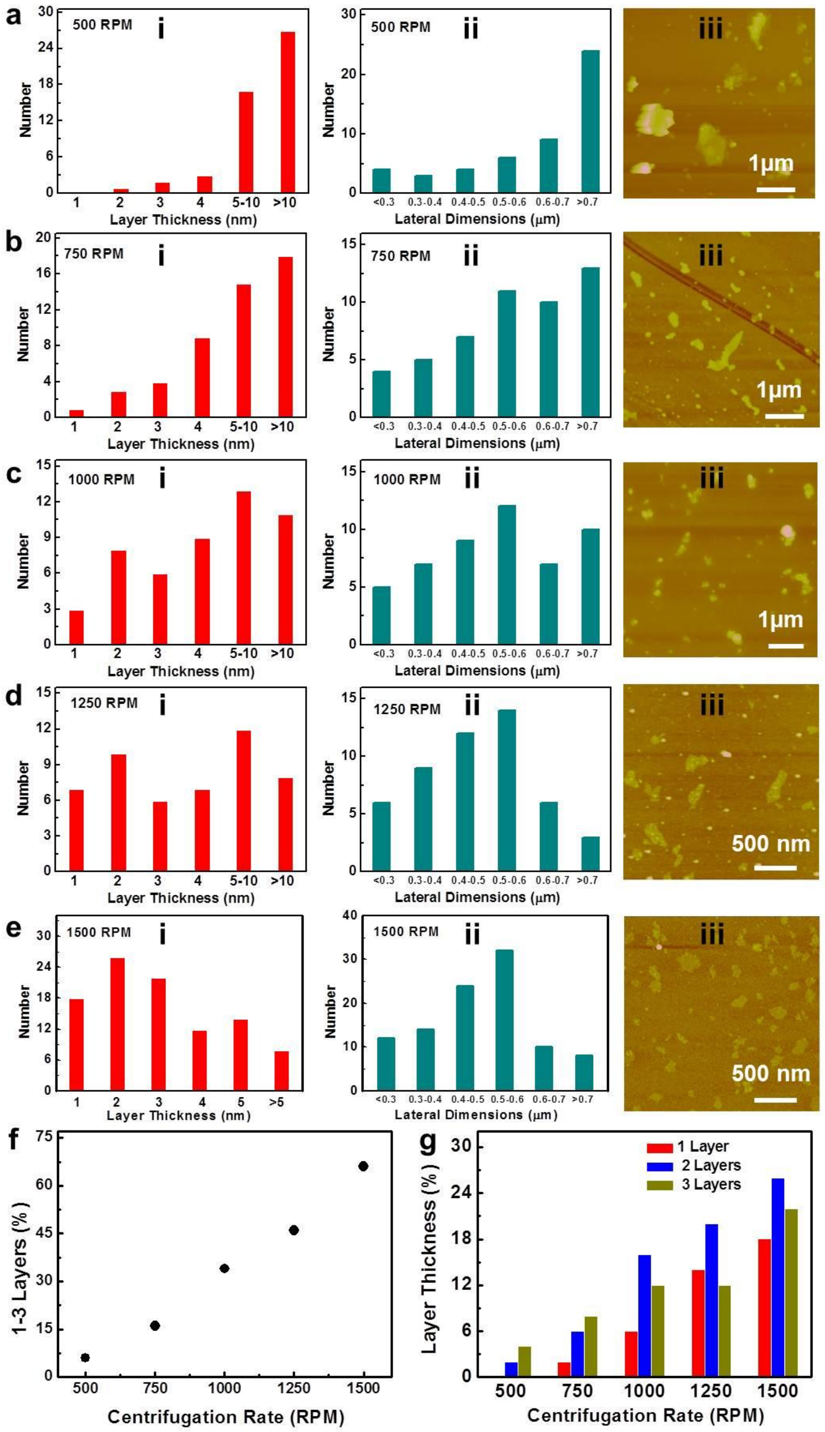

Supplementary Figure 17. Morphology of $MoSe_2$ as a function of centrifugation rate. Histograms showing **a-e,** (**i**) the average layer thickness, and (**ii**) the average size distribution and (**iii**) representative AFM images of the exfoliated $MoSe_2$ nanosheets at different centrifugation rates. **f**, Percentage of 1-3 layers and **g**, distribution of the 1-3 layers of the exfoliated $MoSe_2$ nanosheets as the function of centrifugation rate.

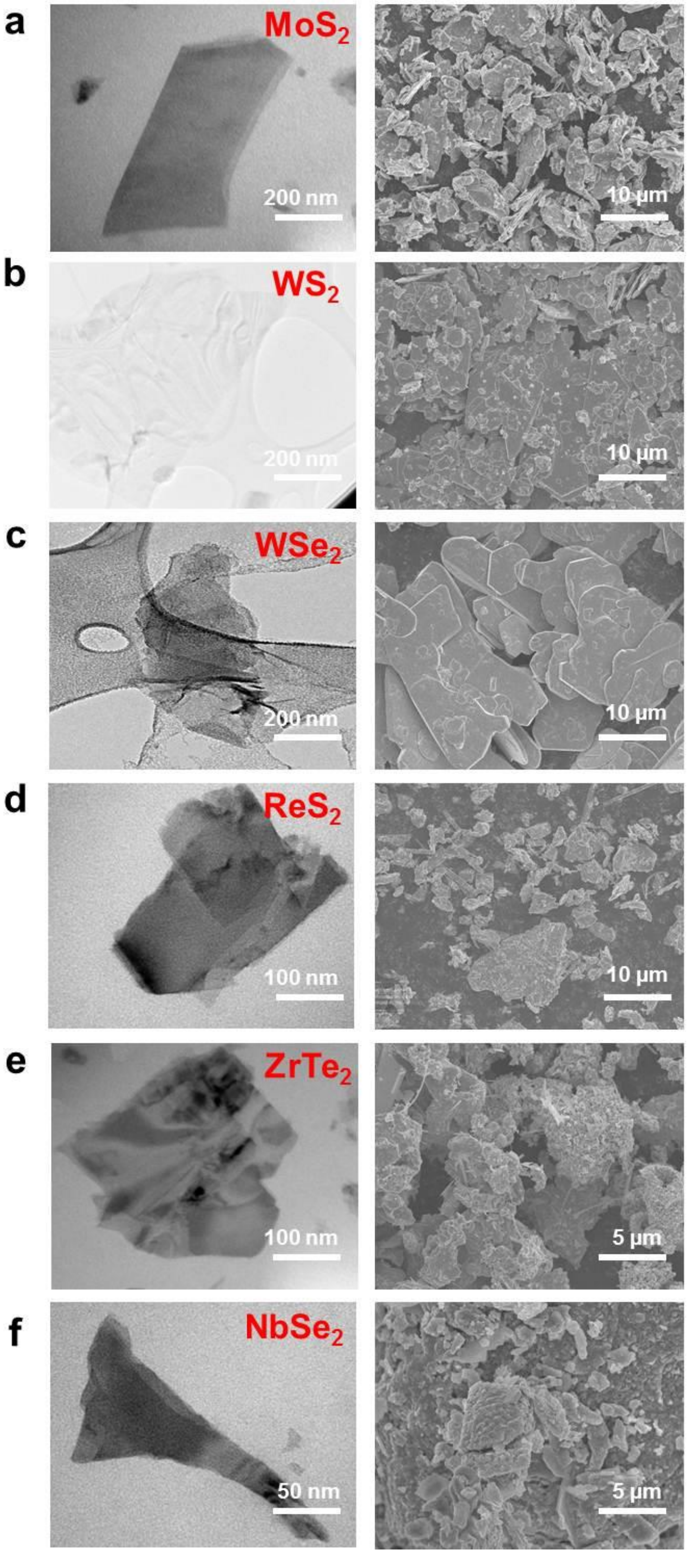

Supplementary Figure 18. **Morphology of the TMD nanosheets.** Bright field TEM images of the different exfoliated TMD nanosheets and SEM images of bulk TMD powders.

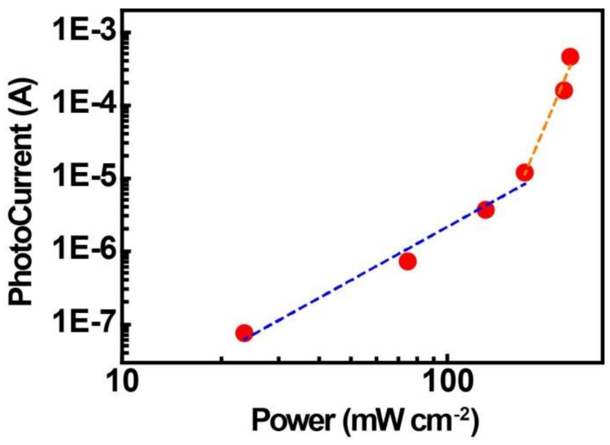

Supplementary Figure 19. **Photodetection performance of $MoSe_2$ nanosheets with PS-NH_2.** Photocurrent values the $MoSe_2$ photodetector as a function of the NIR light intensity at a wavelength of 1,064 nm with a bias voltage of 9 V.

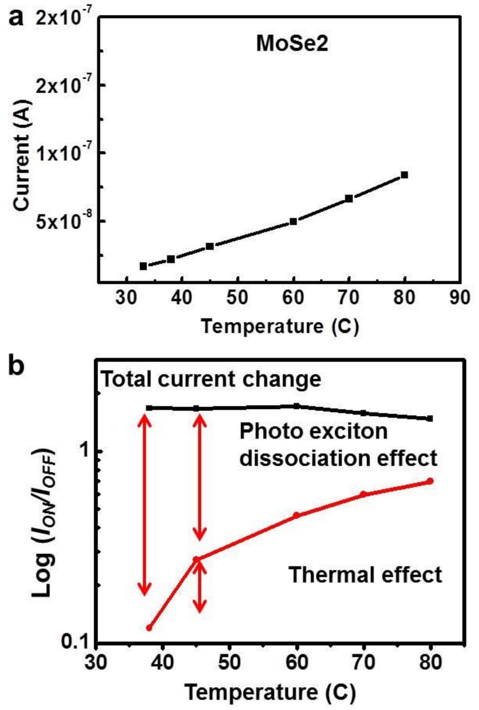

Supplementary Figure 20. **Temperature dependent dark and photocurrent. a**, Dark current variation of thin $MoSe_2$/PS-NH_2 composite film as a function of device temperature with a bias voltage of 9 V. **b**, Log scale ratio of thin $MoSe_2$/PS-NH_2 composite film under NIR light at a wavelength of 1,064 nm to dark conditions as a function of the device temperature. Based on the variation of the dark current at each temperature, the contribution of photocurrent arising from exciton-dissociation was calculated.

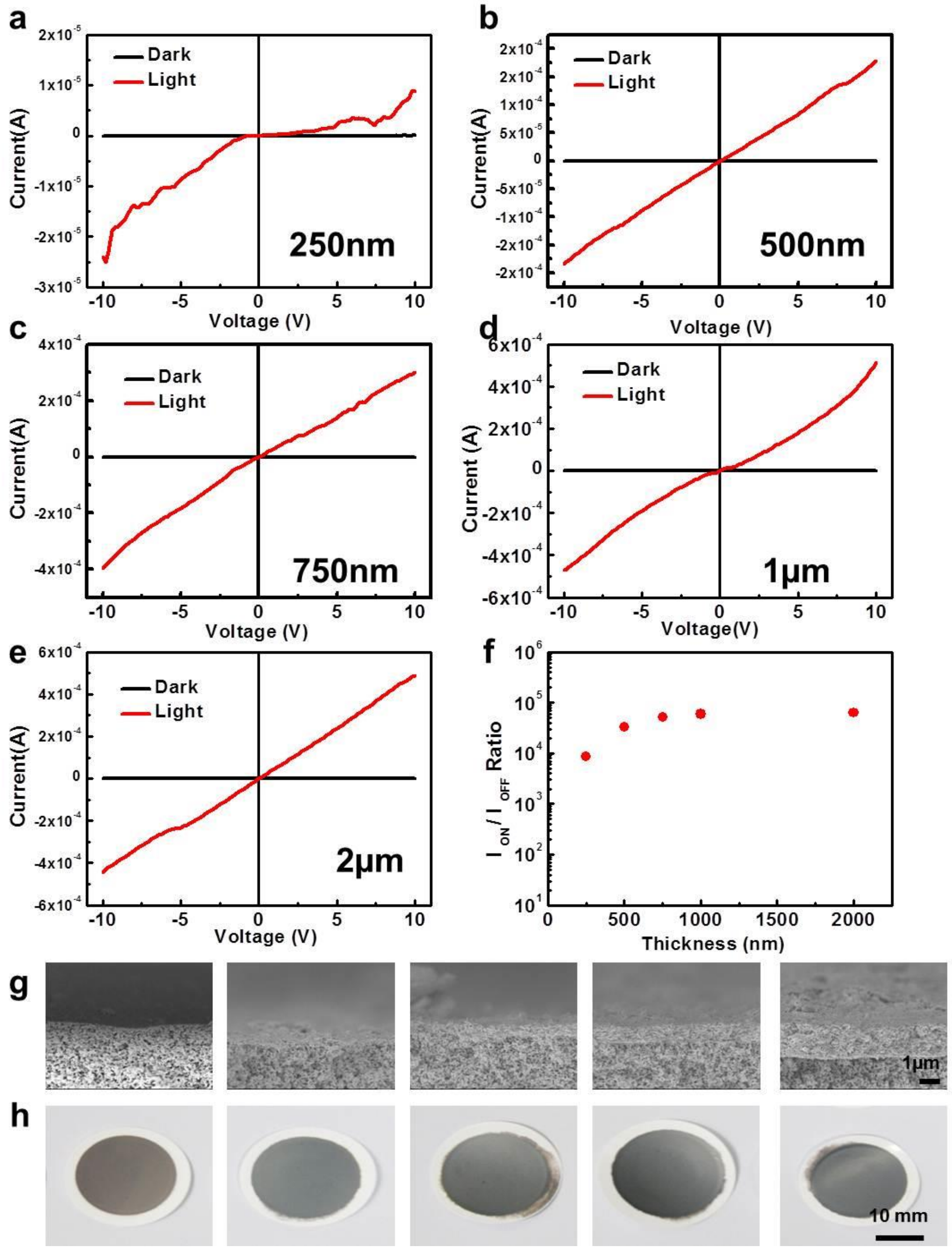

Supplementary Figure 21. Photoresponse as a function of different film thickness. a-e, Linear scale *I-V* characteristics of the $MoSe_2$ composite films with PS-NH_2 with different film thickness measured under dark and a NIR laser at a wavelength of 1,064 nm and a light intensity of 238 mW/cm^2. **f**, Ratio of the photocurrent to the dark current of the $MoSe_2$ composite films with PS-NH_2 as a function of different film thickness. **g**, SEM images of the cross-sectional structure and **h**, photograph of the composite films with different thickness.

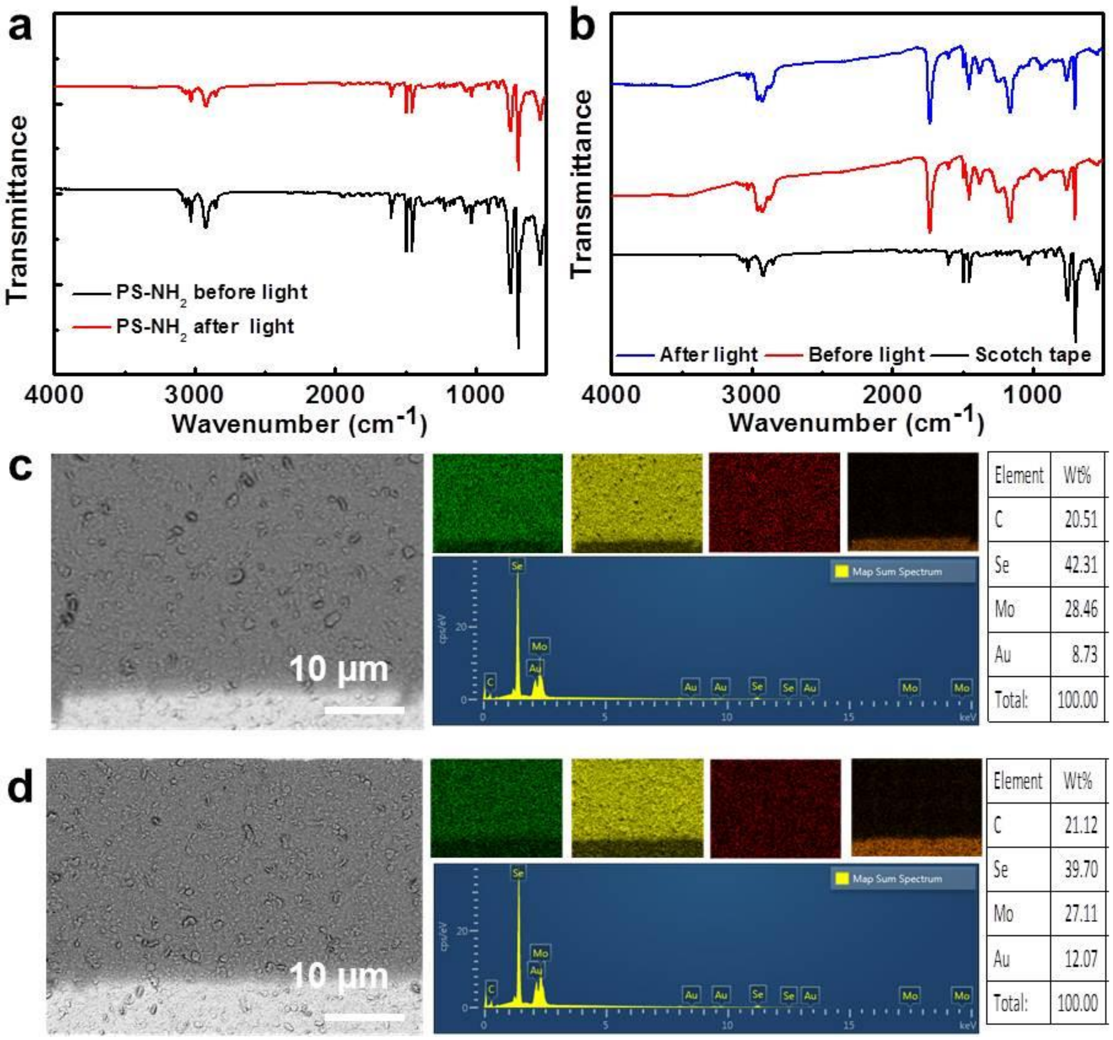

Supplementary Figure 22. Stability of the PS-NH$_2$. a,FT-IR spectrum of the neat PS-NH$_2$ polymer film **b**, MoSe$_2$ composite film with PS-NH$_2$ transferred on scotch tape before and after exposure of NIR laser with the maximum power used for photo-detection experiments. For comparison scotch tape spectrum also included. SEM and EDX spectra of the MoSe$_2$ composite film with PS-NH$_2$ **c**, before and **d**, after exposure of NIR laser with the maximum power used for photo-detection experiments. The absorbance peaks, morphology and chemical compositions were rarely changed when a composite of MoSe$_2$ with PS-NH$_2$ was exposed with NIR and visible laser, which confirms the stability of PS-NH$_2$.

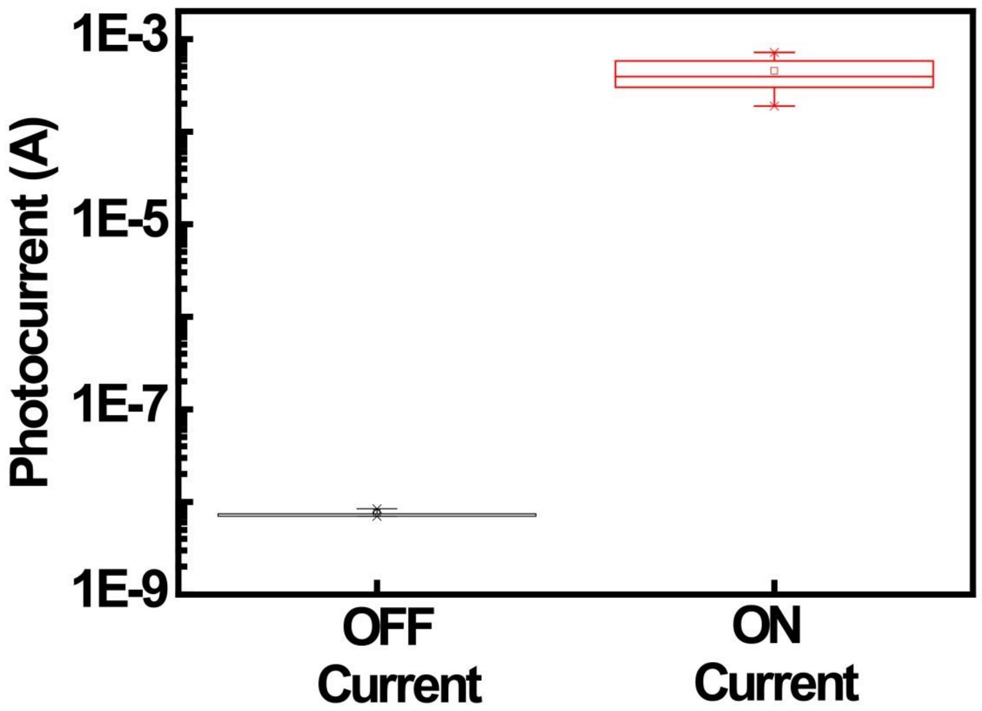

Supplementary Figure 23. **Reliability of the photodetectors.** Box-whisker plot of the distribution of the Off current and On current values of the $MoSe_2$ composite thin film devices. A total of 36 devices obtained from five different batches were measured showing high cell-to-cell and batch-to-batch reliabilities with very small changes for the both the OFF current and ON current values.

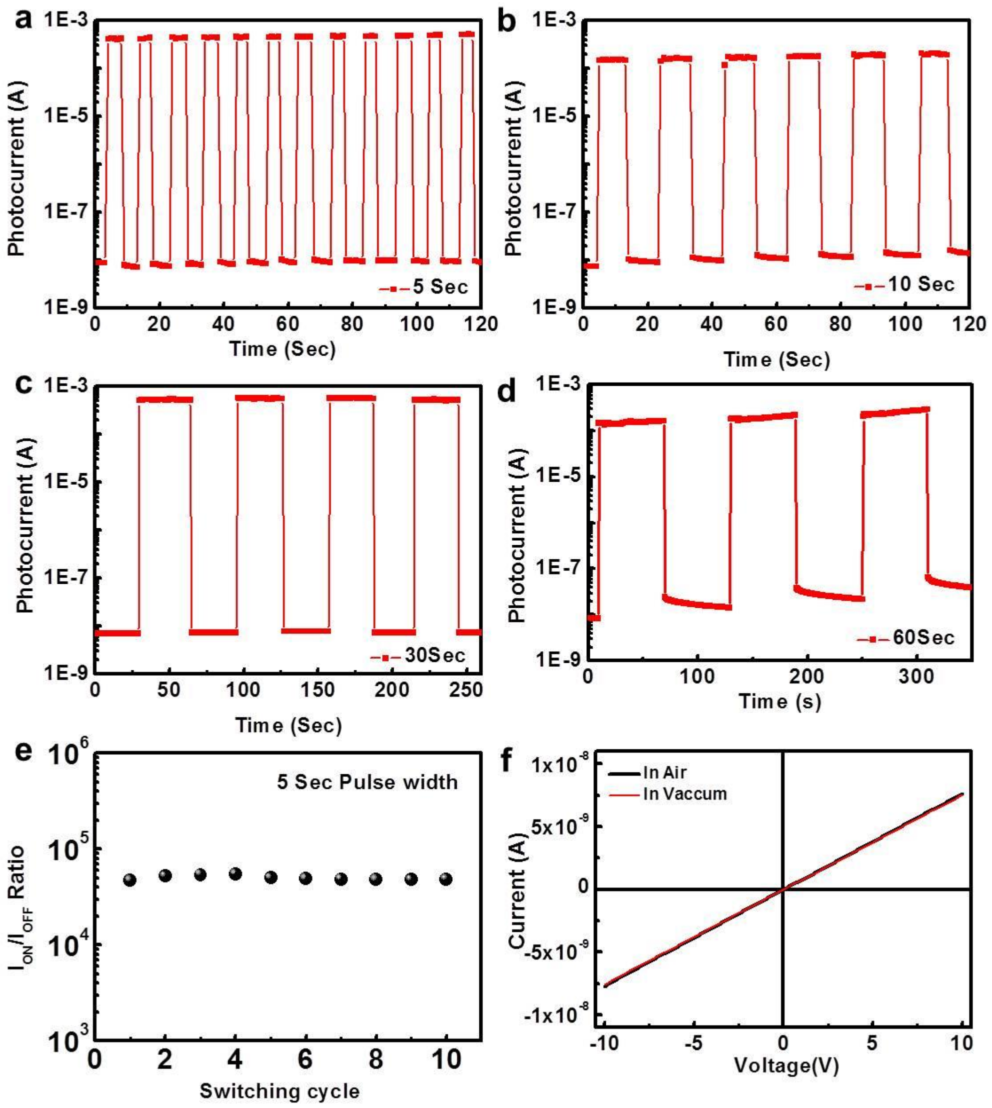

Supplementary Figure 24. **Photoresponse of the thin $MoSe_2$ composite films with PS-NH_2. a-d,** Photoresponse behavior of the $MoSe_2$ photodetector under alternating on and off pulses of NIR light with different pulse widths ranging from 5 to 60 s at an intensity of 238 mW/cm^2. Both the rise and decay times of the photocurrent are approximately 100 ms with a sharp response upon turn-on and -off of pulsed laser illumination, irrespective of the pulse width. **e,** Ratios of the photocurrent to the dark current of the $MoSe_2$ photodetector under alternating on and off pulses of NIR light with 5s pulse width at an intensity of 238 mW/cm^2. **f**, Typical I–V characteristics of a thin $MoSe_2$/PS-NH_2 composite film without light illumination measured in air and under vacuum conditions with a bias voltage of ± 10 V. No difference was observed.

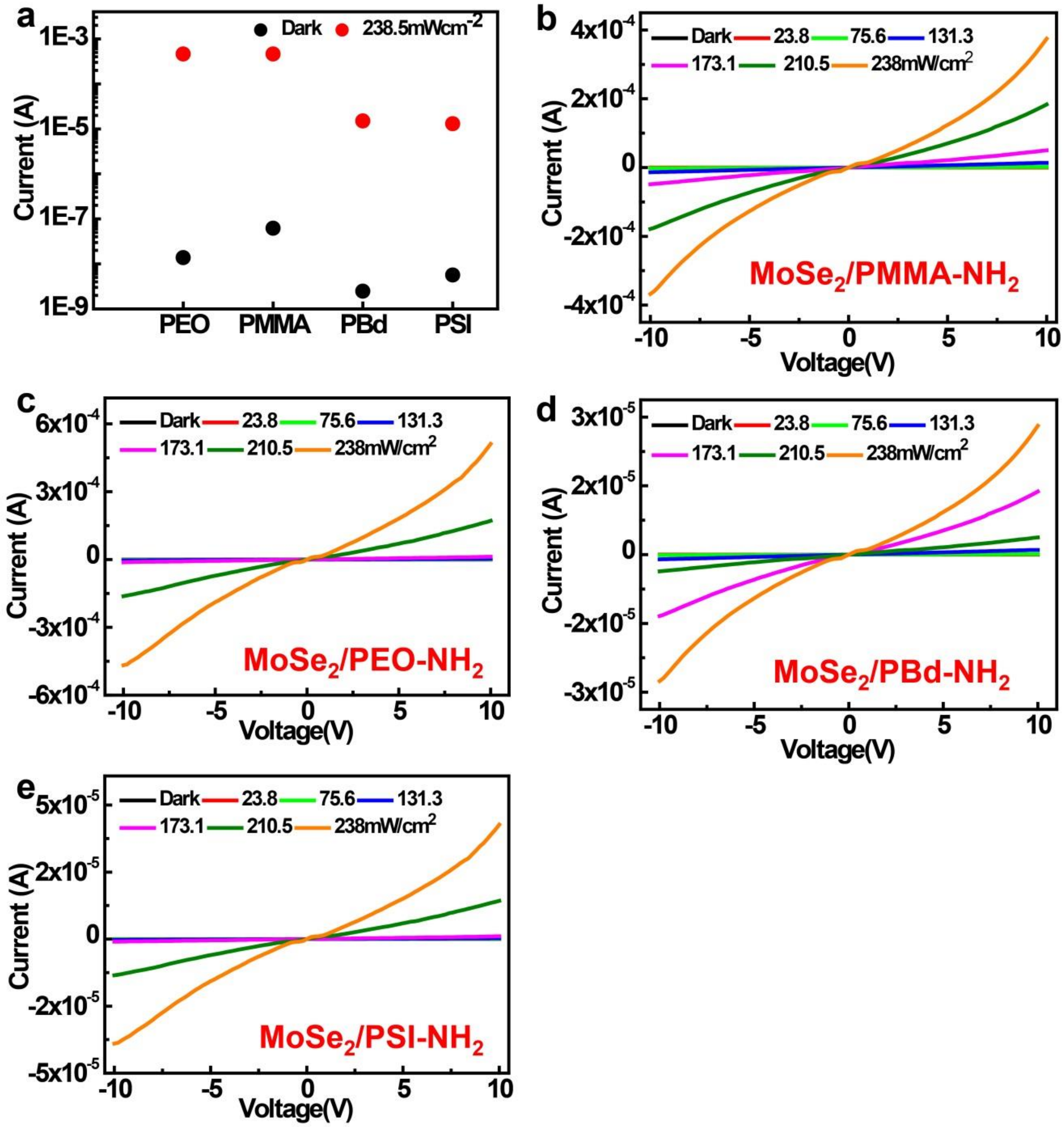

Supplementary Figure 25. **$MoSe_2$ devices with different amine-terminated polymers. a**, The photocurrent and dark current of the $MoSe_2$ photo-detectors with four different amine-terminated polymers measured using a NIR laser at a wavelength of 1,064 nm and a light intensity of 238 mW/cm^2. **b-e**, Linear scale *I-V* characteristics of the NIR photodetectors containing $MoSe_2$ nanosheets exfoliated with amine-terminated polymers such as PEO, PMMA, PB, and PSI as a function of the light intensity with a bias voltage of ± 10 V.

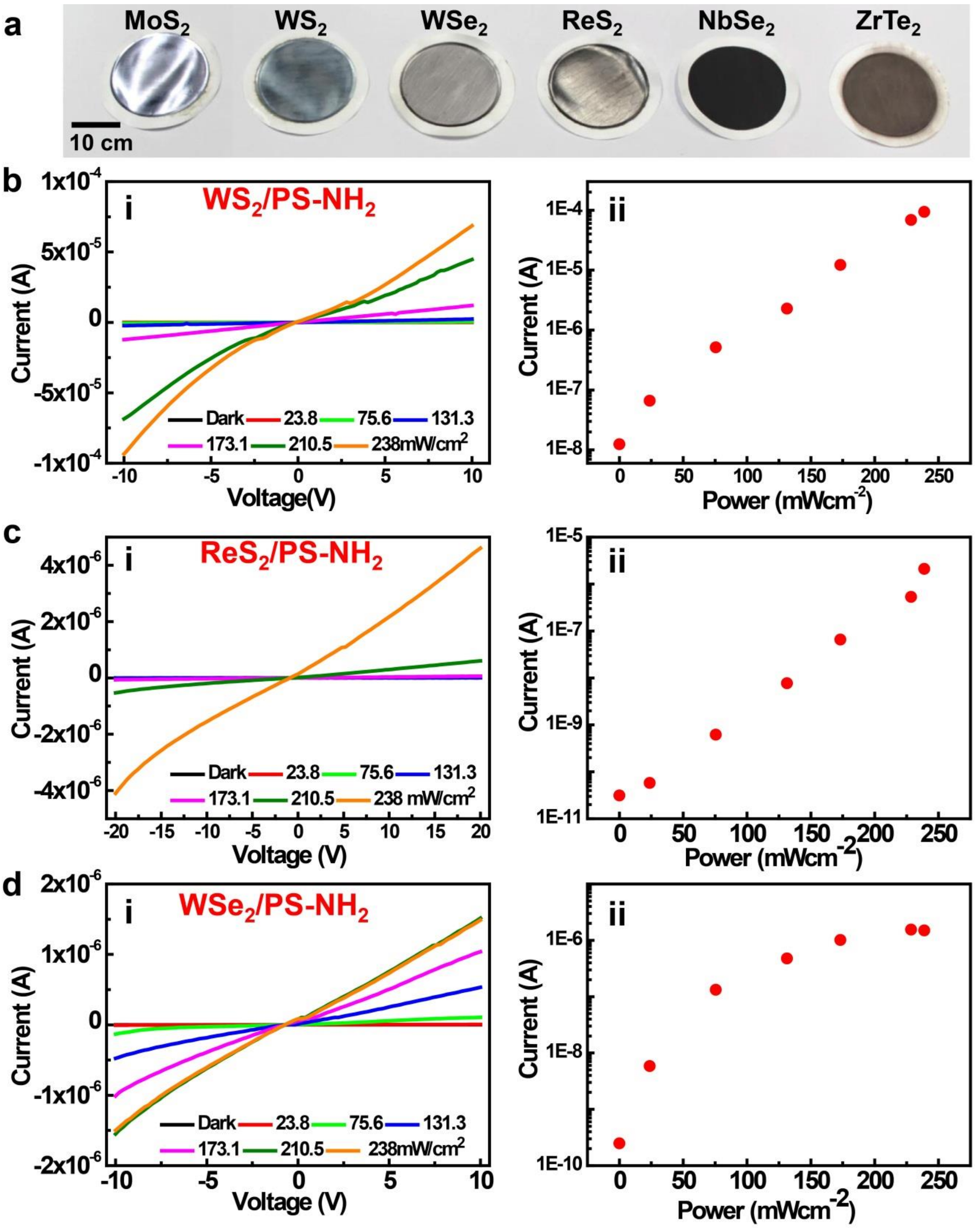

a
MoS2
WS2
WSe2
ReS2
NbSe2
ZrTe2
10 cm
b
i
WS2/PS-NH2
Current (A)
Voltage(V)
Dark 23.8 75.6 131.3
173.1 210.5 238mW/cm2
ii
Current (A)
Power (mWcm-2)
c
i
ReS2/PS-NH2
Current (A)
Voltage (V)
Dark 23.8 75.6 131.3
173.1 210.5 238 mW/cm2
ii
Current (A)
Power (mWcm-2)
d
i
WSe2/PS-NH2
Current (A)
Voltage (V)
Dark 23.8 75.6 131.3
173.1 210.5 238mW/cm2
ii
Current (A)
Power (mWcm-2)

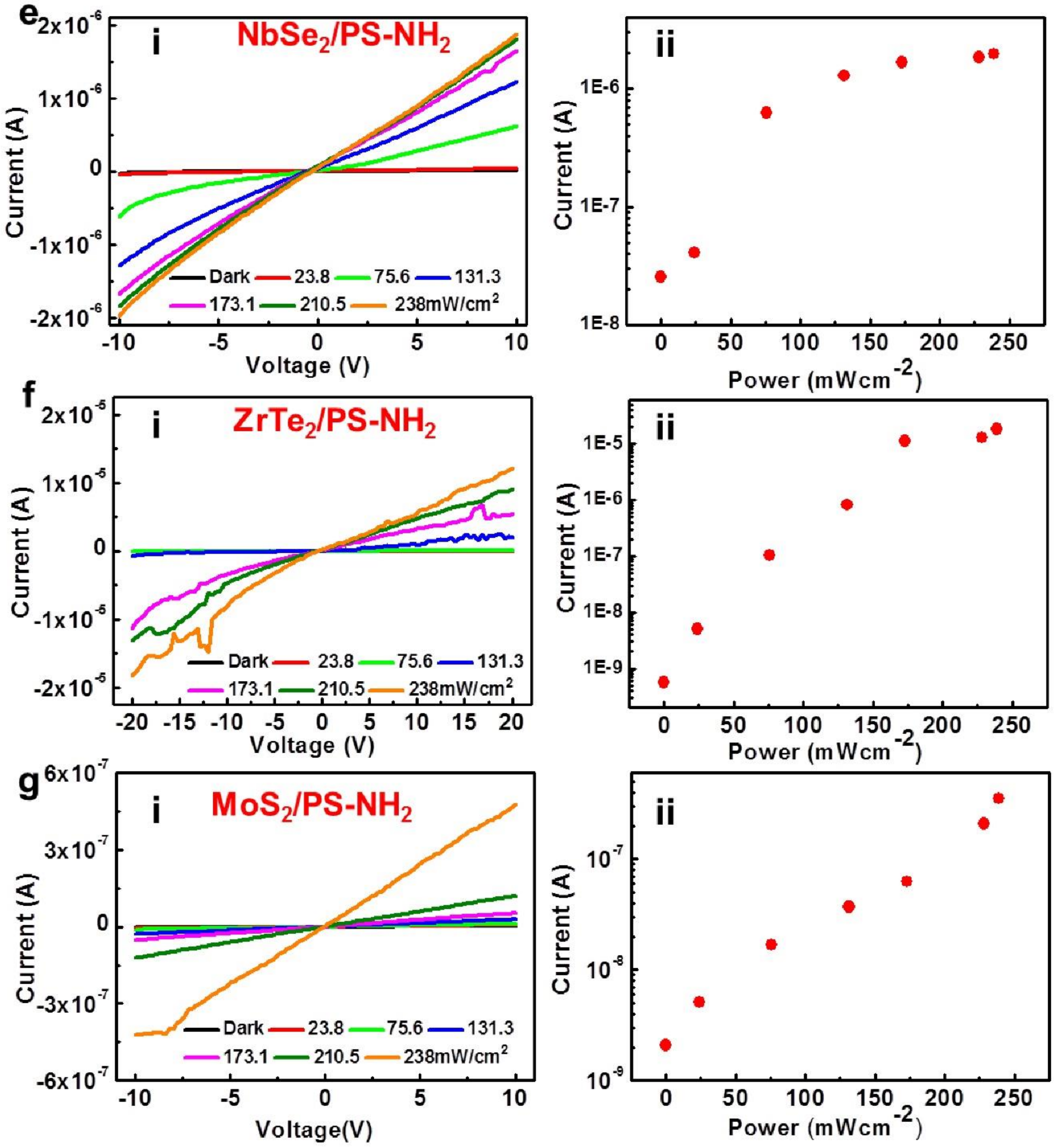

Supplementary Figure 26. **Photo-detectors with various TMDs exfoliated with PS-NH_2. a**, Photograph of thin TMD/PS-NH_2 composite thin films on conventional filter paper. **b-g**, (i) Linear scale *I-V* characteristics of a thin $MoSe_2$/PS-NH_2 composite film under exposure of NIR light at 1,064 nm with different light intensities as a function of the bias voltage.(ii), Photocurrent values of the composite films at a bias voltage of 9 V as a function of the light intensity.

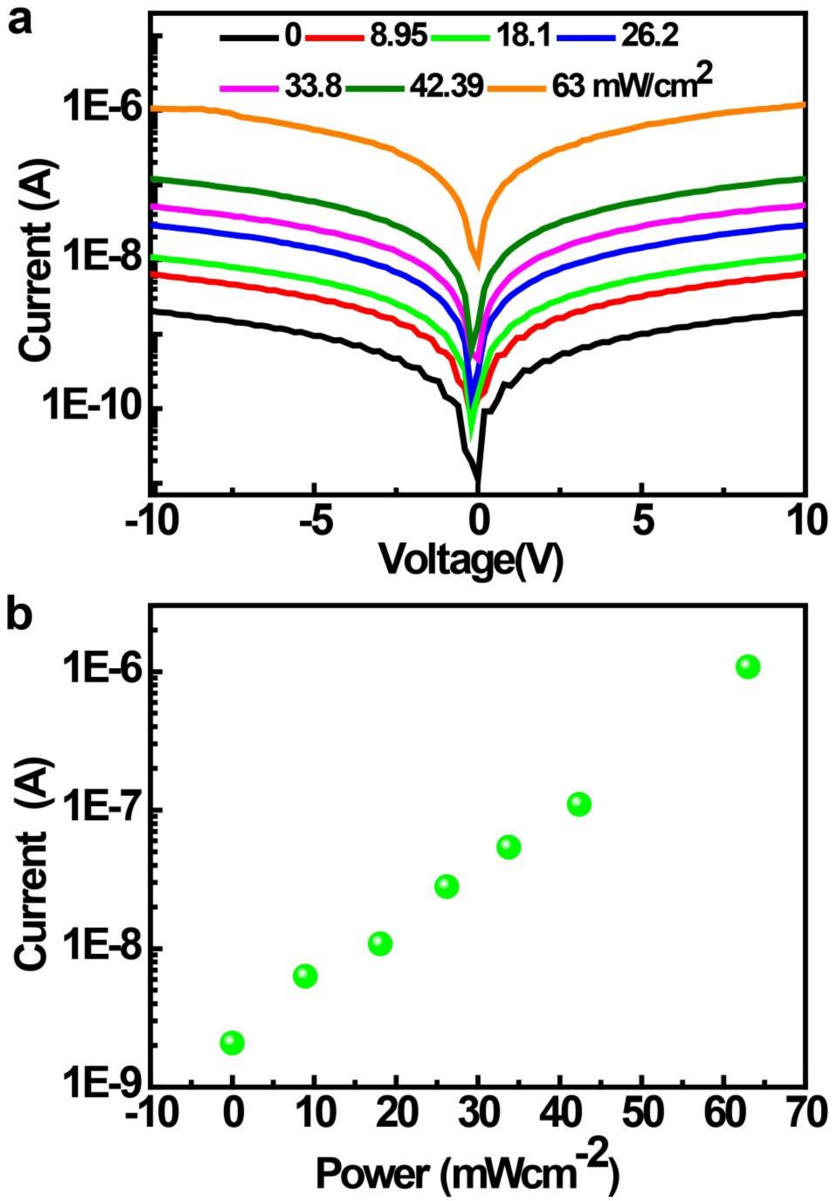

Supplementary Figure 27. **Photodetectors of MoS_2 nanosheets exfoliated with PS-NH_2.** **a**, *I*-*V* characteristics of a photo-detector with a thin MoS_2/PS-NH_2 composite film in the dark and under different light intensities of visible light at a wavelength of 532 nm with a bias voltage of ± 10 V. **b**, Photocurrent values as a function of the light intensity.

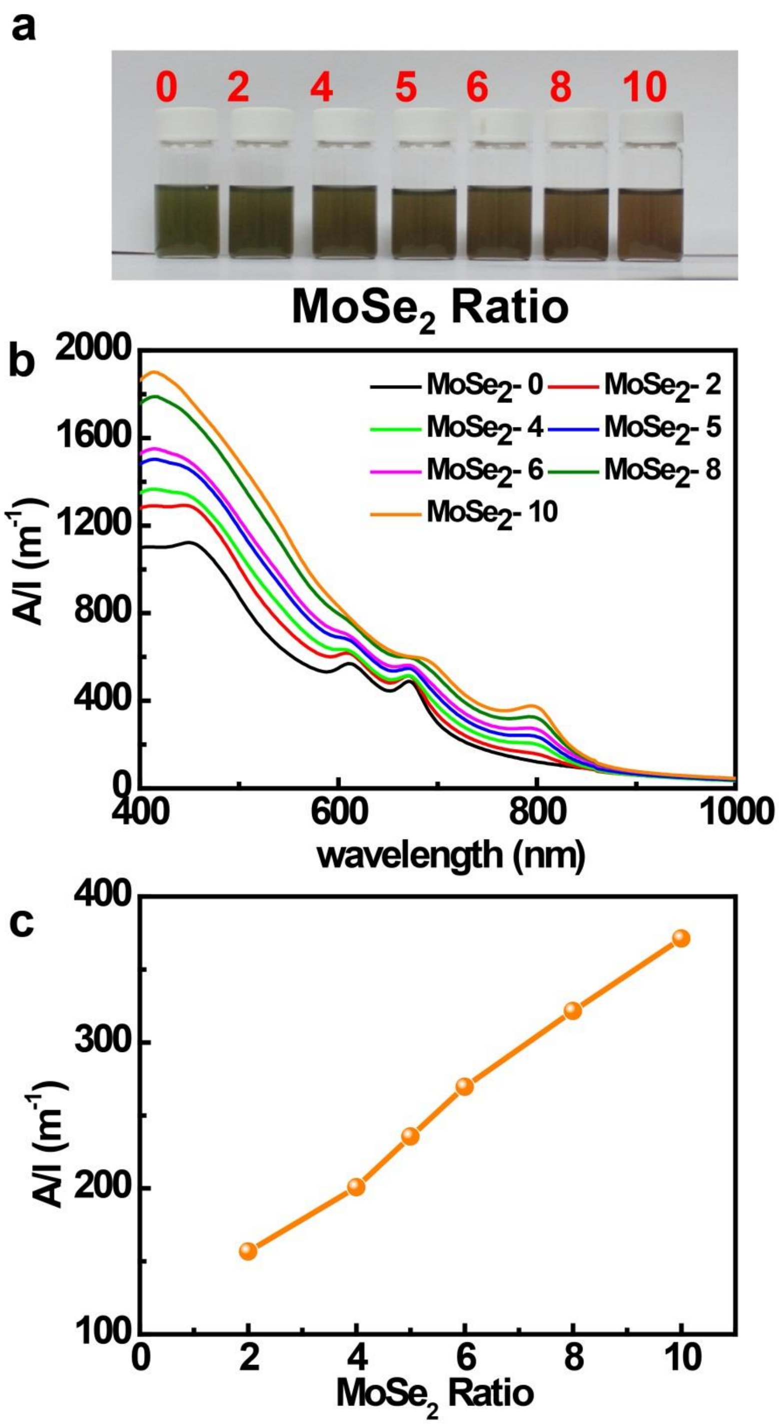

Supplementary Figure 28. **Homogeneous mixing of $MoSe_2$ and MoS_2 nanosheets with PS-NH_2. a**, Photograph of the solution blends of $MoSe_2$ and MoS_2 nanosheets dispersed with PS-NH_2 in toluene as a function of the $MoSe_2$ to MoS_2 ratio. **b**, Absorbance spectra of the solution blends and **c**, absorbance values measured at 800 nm as a function of the $MoSe_2$ to MoS_2 ratio.

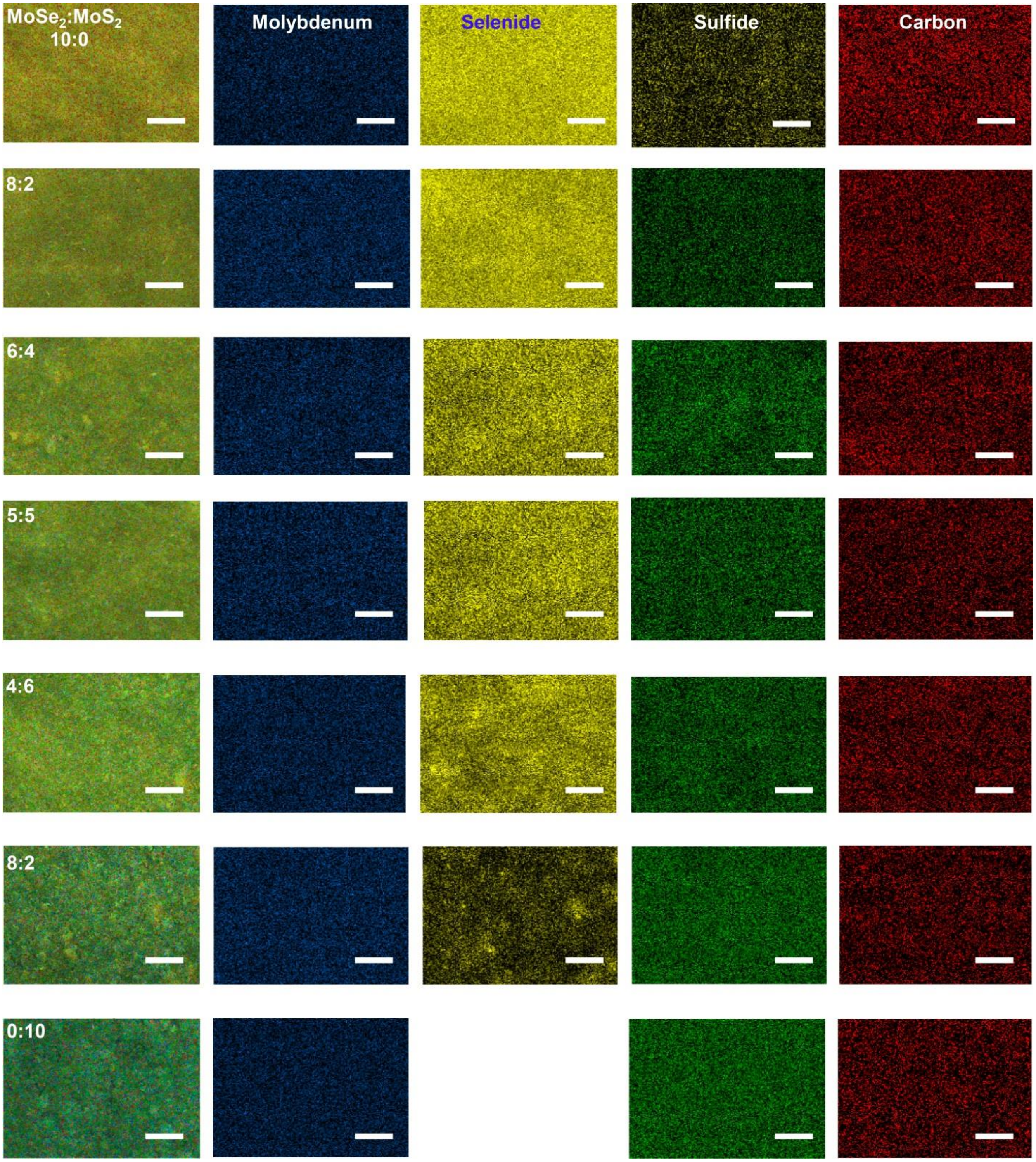

Supplementary Figure 29. **EDX mapping of the constituent atoms of $MoSe_2$ and MoS_2.** Elemental mapping of the different compositions of $MoSe_2$ and MoS_2 mixtures with PS-NH_2 (Mo-Blue, Se-Yellow, S-Green, C-Red). In all cases, the scale bar is 2 μm.

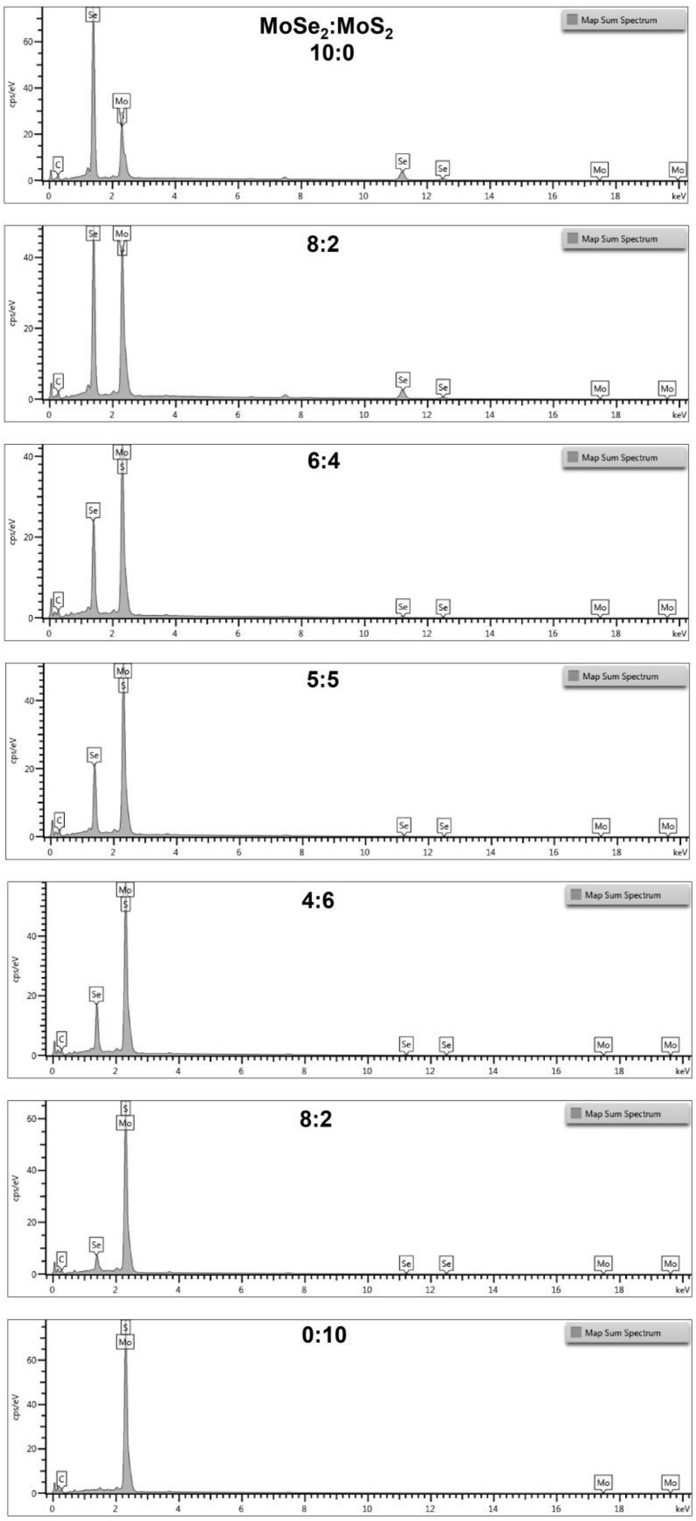

Element	Wt%
C	17.95
Se	50.74
S	1.19
Mo	30.12
Total:	100

Element	Wt%
C	18.82
S	10.34
Se	35.75
Mo	35.09
Total:	100

Element	Wt%
C	16.61
S	18.42
Se	24.61
Mo	40.36
Total:	100

Element	Wt%
C	16.23
S	21.03
Se	20.24
Mo	42.49
Total:	100

Element	Wt%
C	16.2
S	23.7
Se	15.83
Mo	44.27
Total:	100

Element	Wt%
C	15.45
S	30.04
Se	6.12
Mo	48.38
Total:	100

Element	Wt%
C	16.5
S	34.28
Mo	49.22
Total:	100

Supplementary Figure 30. **The relative atomic intensities in $MoSe_2$ and MoS_2 mixtures.** Seven different compositions of $MoSe_2$ and MoS_2 mixtures were examined with PS-NH_2.

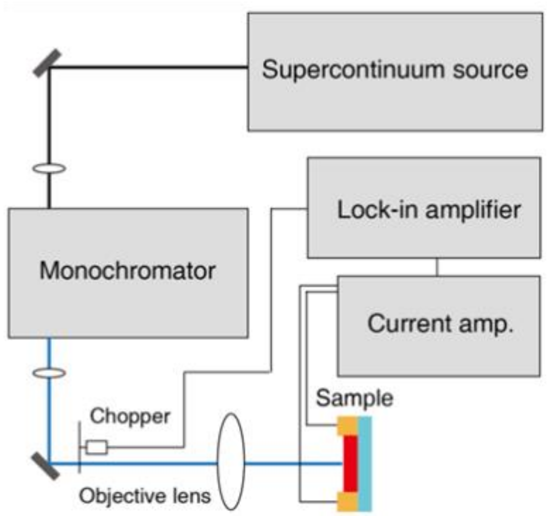

Supplementary Figure 31. **Photocurrent measurement with high selectivity system.** Schematic of the photocurrent spectrum measurements.

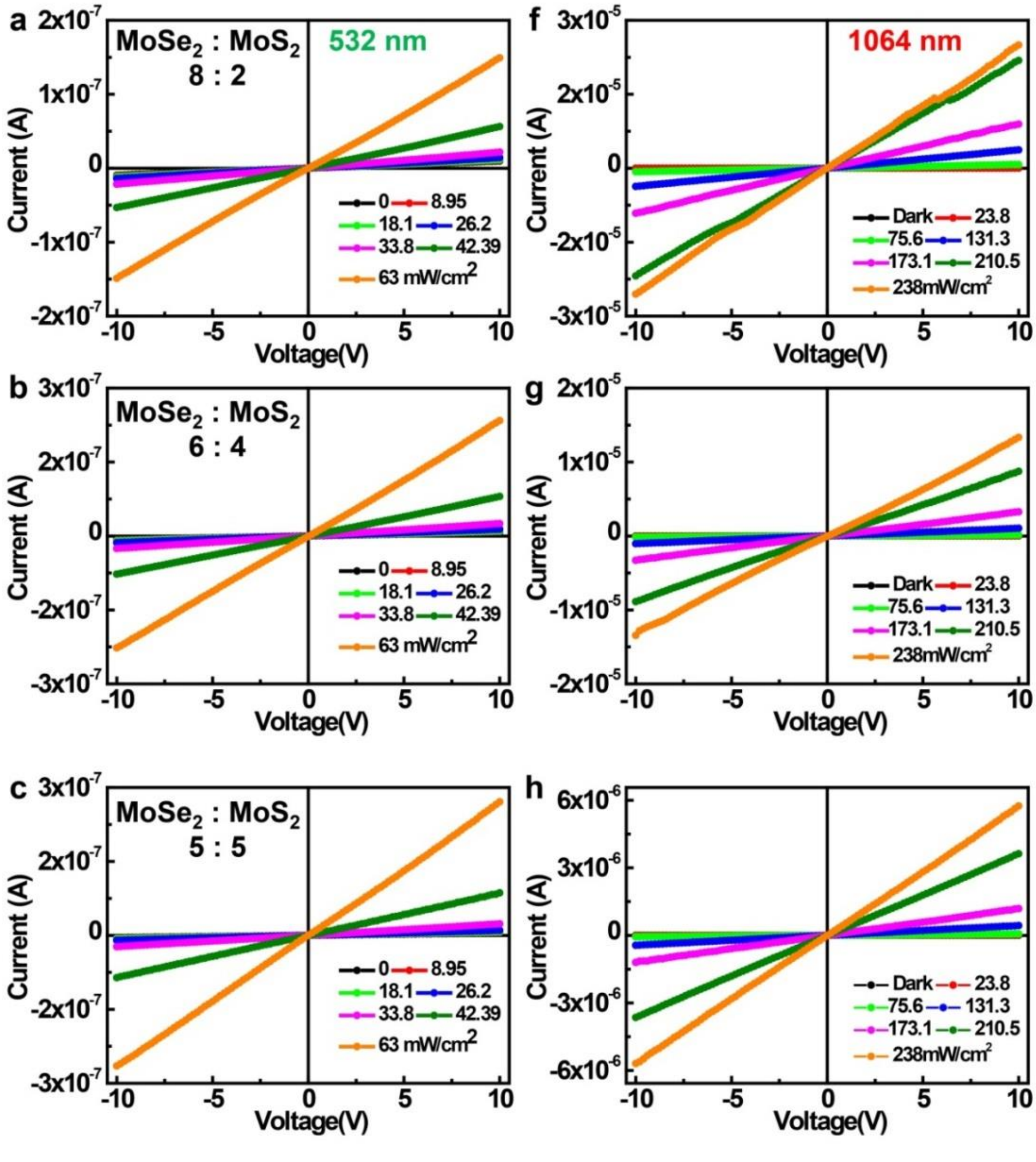

a
MoSe2 : MoS2
8 : 2
532 nm
Current (A)
Voltage(V)
0
8.95
18.1
26.2
33.8
42.39
63 mW/cm2
b
MoSe2 : MoS2
6 : 4
c
MoSe2 : MoS2
5 : 5
f
1064 nm
Dark
23.8
75.6
131.3
173.1
210.5
238mW/cm2
g
h

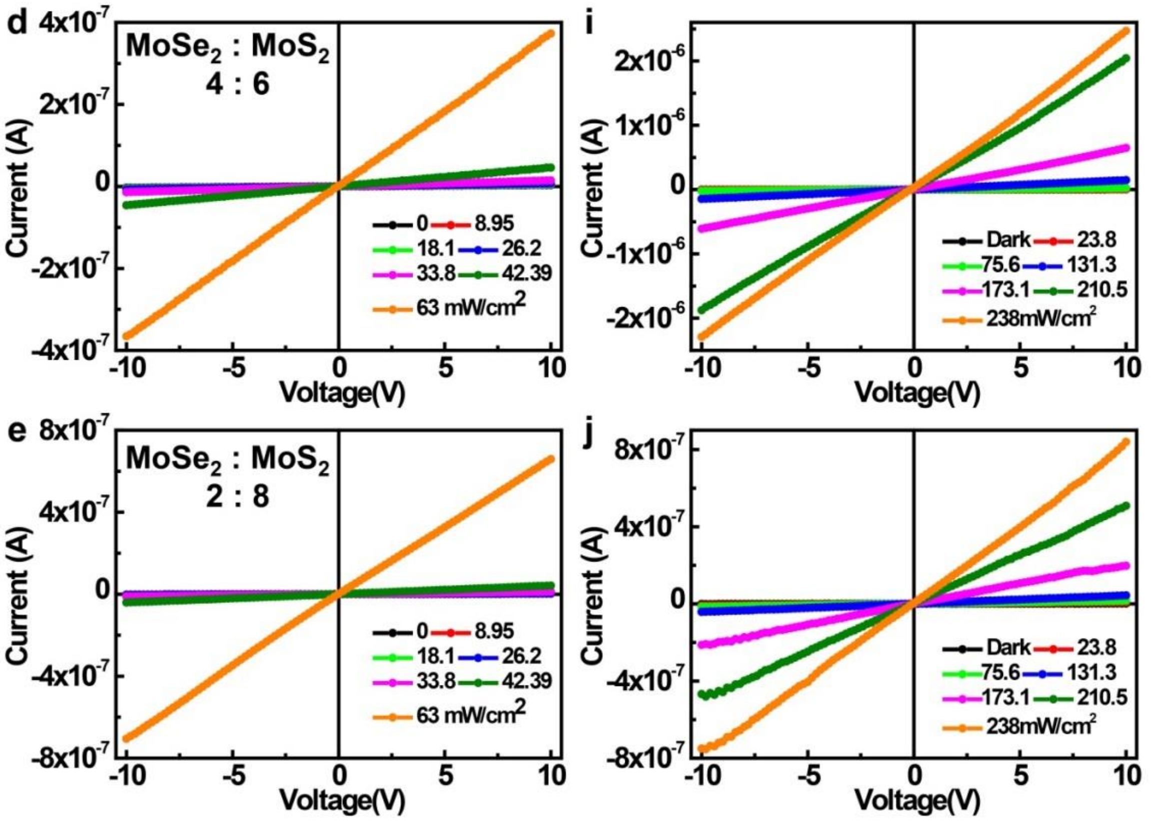

Supplementary Figure 32. Photodetection performance of thin MoS_2:$MoSe_2$ composite films. Linear scale *I*-*V* characteristics of different blend ratio of thin $MoSe_2$ and MoS_2 composite films with PS-NH_2 at wavelengths of **a-e**, 532 nm and **f-j**, 1,064 nm in the dark and under different light intensities.

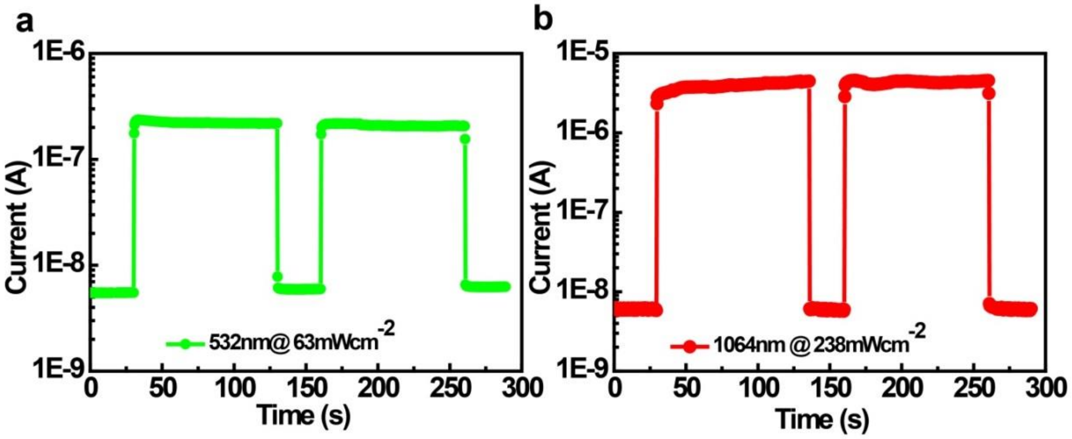

Supplementary Figure 33. Photoresponse of blended composites of MoS2:MoSe2 (5:5) films. Photoresponse behavior of the blended composite photodetector under alternating on and off cycles of **a**, visible light and **b**, NIR light with intensities of 63 and 238 mW/cm^2, respectively.

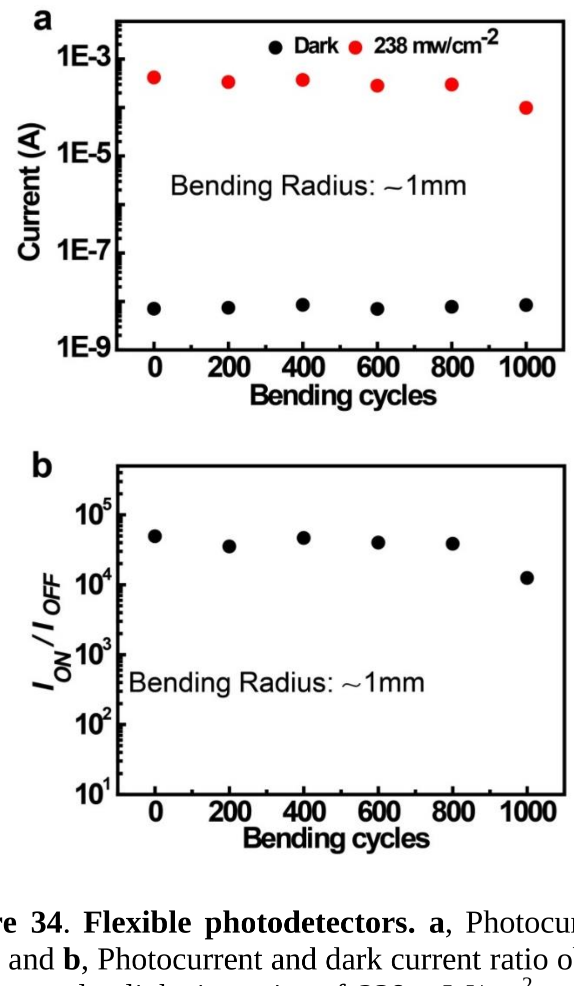

Supplementary Figure 34. **Flexible photodetectors. a**, Photocurrent and dark current of the $MoSe_2$/ PS-NH_2 device and **b**, Photocurrent and dark current ratio obtained using a NIR laser at a wavelength of 1,064 nm and a light intensity of 238 mW/cm^2 as a function of the number of bending cycles at a banding radius of approximately 1 mm.

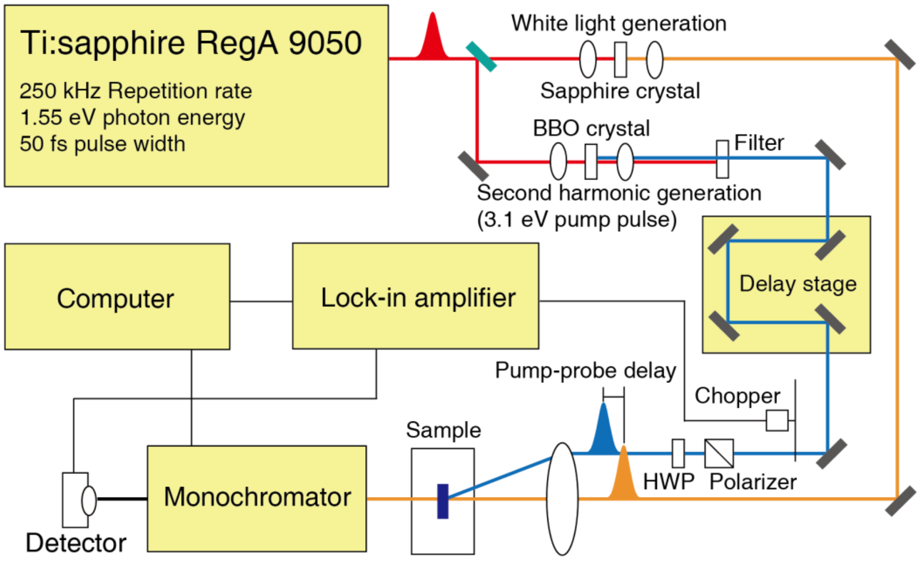

Supplementary Figure 35. **Experimental set-up.** A scheme of the ultrafast optical-pump and optical-probe spectroscopy.

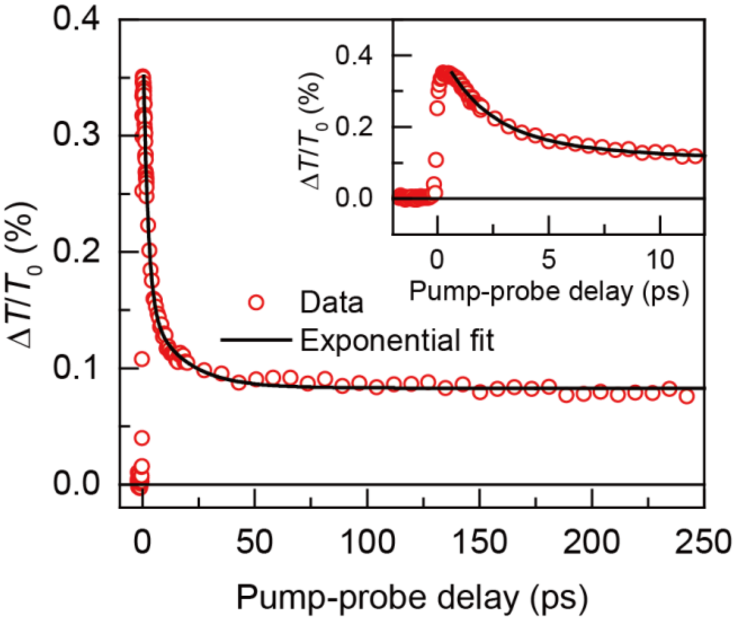

Supplementary Figure 36. **Transient relaxation dynamics of $MoSe_2$ thin films.** Transient relaxation dynamics of $MoSe_2$ and corresponding tri-exponential fitting. The probe wavelength is 700 nm with 67 μJ/cm^2 pump fluence. Inset: zoom-in relaxation dynamics of the fast decay signal.

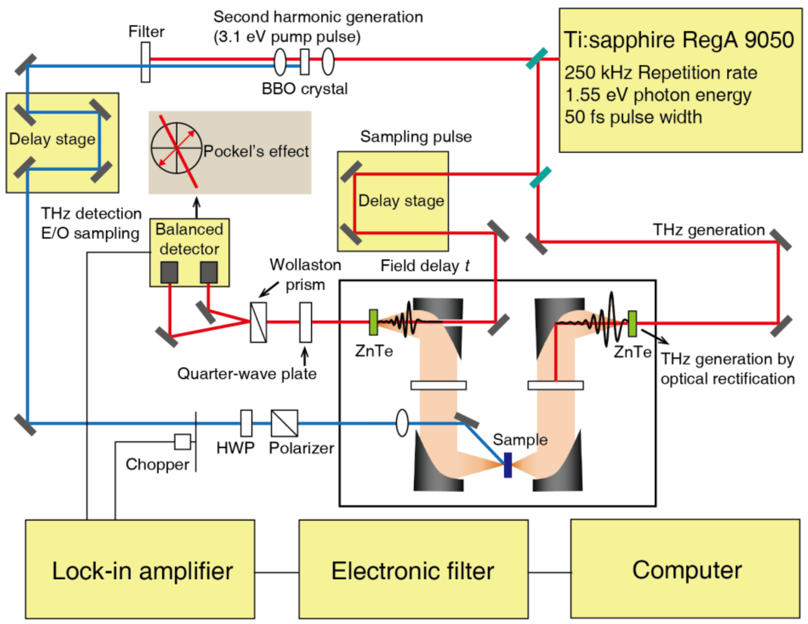

Supplementary Figure 37. **Scheme of ultrafast optical-pump and THz-probe spectroscopy.**

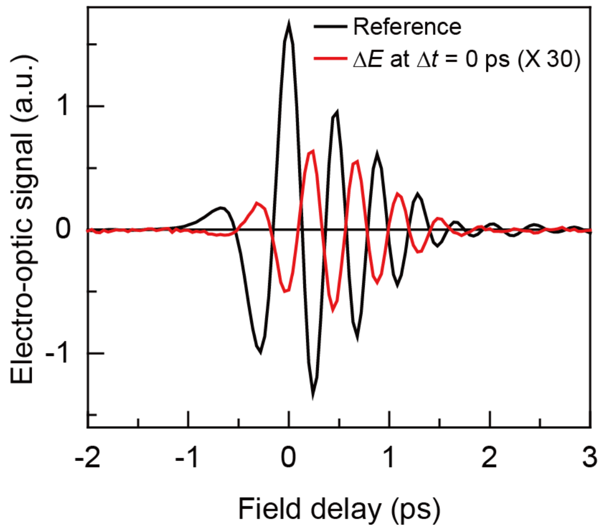

Supplementary Figure 38. **Electro-optic signal as a function of the THz-field delay**. Electro-optic reference field (black line) and differential signal, ΔE (red line), of few-layer MoS_2 nanosheets exfoliated with PS-NH_2 are shown. ΔE is magnified 30 times.

**TMDs**	**Mol.Wt**	**Size**	**Density**	**Supplier**	**Product Number**
Molybdenum (IV) selenide **$MoSe_2$**	253.87	-325 Mesh Powder	6	Alfa Aesar	13112
Molybdenum (IV) sulfide **MoS_2**	160.07	~6 µm	5.06	Sigma Aldrich	69860
Tungsten (IV) selenide **WSe_2**	341.77	~10 µm	9.32	Alfa Aesar	13084
Tungsten (IV) sulfide **WS_2**	247.98	No data	7.5	Alfa Aesar	11829
Rhenium (IV) sulfide **ReS_2**	250.33	No data	7.506	Alfa Aesar	89482
Niobium (IV) selenide **$NbSe_2$**	250.83	~5 µm	No data	Alfa Aesar	13101
Zirconim (IV) telluride **$ZrTe_2$**	346.42	-325 Mesh Powder	6.42	Alfa Aesar	36321

Supplementary Table 1. Physical properties of the TMDs used in this study.

Polymer	Structure	M_nx 10^3	PDI	Tg (^{O} C)	NH_2 Functionality
Polystyrene<br>**PS**	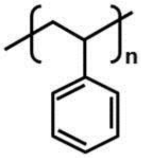	11.5	1.04	90	------
Amine Terminated Polystyrene<br>**PS-NH_2**	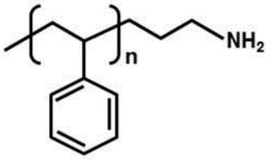	9.5	1.16	102	>98%
		25	1.35	102	>98%
		40	1.05	102	>98%
		108	1.12	102	>98%
Amine Terminated Polymethyl methaccrylate<br>**PMMA-NH_2**	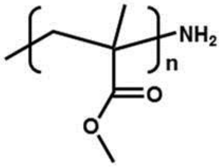	20	1.2	131	>98%
Amine Terminated Polyethylene<br>**PE-NH_2**	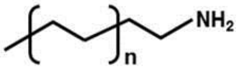	4.2	1.05	None<br>Tm - 102<br>Tc - 89	>98%
Amine Terminated Polybuda -diene<br>**PBd-NH_2**	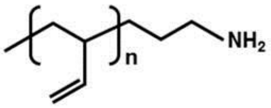	15	1.08	-14	>98%
Amine Terminated Polyethylene Oxide<br>**PEO-NH_2**	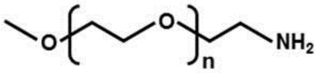	9.5	1.07	No Data	>95%
Amine Terminated Polystyrene-*b*-isoprene<br>**PS-*b*-PI-NH_2**	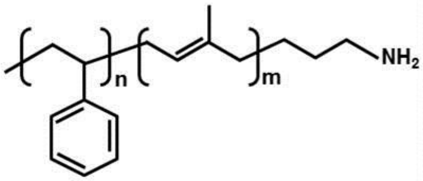	23-*b*-37.5	1.06	No Data	>90%

Supplementary Table 2. Physical properties of the polymers used in this study.

Supplementary Note 1. Mechanism for the exfoliation and dispersion of various TMDs.

- IR calculation of TMDs with amine-terminated polymers

We performed the computation of IR spectra for model systems ($PS-NH_2$, $MoSe_2/PS-NH_2$) based on density functional theory (DFT) frequency calculation employing B3LYP functional[1] with LANL2DZ basis set [2] as implemented in Gaussian 09 program [3]. For the sake of simplicity, the $PS-NH_2$ is modeled as an amine-terminated styrene monomer (H-Styrene-CH_2-NH_2) and the $(MoSe_2)n$ sheet is modeled as a Mo_4Se_8 cluster. The geometries were first optimized in the gas phase and the frequency calculations of the optimized geometries were then performed with the consideration of solvent (toluene) effect using IEF-PCM solvent model [4-6]. As seen in **Supplementary Figure 1**, the peak at 1673.94 cm^{-1} for scissoring (bending) vibration of the NH_2 in the absence of $MoSe_2$ is shifted to higher frequency (1684.61 cm^{-1}) in the $MoSe_2/PS-NH_2$ bound state, which consolidates our experimental observation.

- Exfoliation and dispersion mechanism of TMDs using different types of amine-terminated end-functionalized polymers.

We selected $MoSe_2$ and amine-terminated polystyrene ($PS-NH_2$) as examples and investigated them in detail to demonstrate the effectiveness of our proposed strategy. Our method is different from metal ions and small molecules intercalation and further sonication assisted exfoliation methods, because there is no intercalation step of TMDs with amine-terminated polymers in our process. It is indeed difficult to imagine that polymer molecules highly swollen in solvent medium with their radius of gyration of approximately a few nanometers are inserted into such a small gap of two stacked nanosheets (~ 1 nm). The root mean square radius of gyration ($\{<s>^2\}^{1/2}$) of the $PS-NH_2$ (9.5K) is approximately 4 nm in toluene, based on the equation of $\{<s>^2\}^{1/2} = \alpha(C_\infty)^{1/2}n^{1/2}l$, where α is expansion parameter in toluene, C_∞

characteristic ratio of PS, *n* the number of bonds and *l* corresponds to C-C bond length. Instead, amine groups of polymers more efficiently interact with transition metals when surface of TMD nanosheets is exposed upon sonication step and the anchored polymers on the surface of TMDs in turn stabilize the dispersion of nanosheets in solvent.

To confirm our argument, we separated the processes. A solution of bulk $MoSe_2$ in toluene was sonicated without the addition of PS-NH_2 and subsequently mixed with PS-NH_2 solution which had been prepared. (**Supplementary Figure 2a**). While a solution without polymer was immediately settled down in few minutes, the mixed solution exhibited TMDs well dispersed in toluene. (**Supplementary Figure 2b**). The quantitative analysis of the dispersion of $MoSe_2$ suggests that no significant difference in the dispersion was observed between single and two step processes, which implies that amine terminated polymers play a role of mainly stabilizing TMD sheets in solvent and preventing them from re-aggregating upon film formation. Another experiment we designed was to examine the dispersion of $MoSe_2$ with PS-NH_2 without sonication step. When a mixture of $MoSe_2$ and PS-NH_2 was gently stirred in toluene, nanosheets were rarely dispersed in solvent as expected. (**Supplementary Figure 2b**).

The (002) peak shift to low 2 theta angle observed due to the efficient intercalation of TMDs by small molecules in the previous study was not, therefore, observed in our process but the (002) peak was substantially reduced in intensity due to highly exfoliated nanosheets separated by intervening polymers. (**Supplementary Figures 2c and d**). The absorbance spectra of the $MoSe_2$ dispersions obtained under two step process (**Supplementary Figure 2e**) shows similar characteristic peaks of exfoliated $MoSe_2$ obtained by one step process and other methods without PS-NH_2, confirming the exfoliation and stable dispersion of $MoSe_2$ in toluene by our method [7,8].

To further investigate the effect of amine-terminated end-functionalized polymers as a dispersant, we simulate the physic-sorption of amine terminated PS (PS-NH_2) and methyl-terminated PS (PS-CH_3) onto TMD (TiS_2) surface using density functional tight binding (DFTB) [9-14] combined with Born-Oppenheimer molecular dynamics (MD) simulation. Since DFTB parameter sets for Mo and Se interacting with amine were not available in our system, we instead examined a common TMD, TiS_2 whose electronic structure is similar to $MoSe_2$. **Supplementary Figure 3a** compares the simulated concentration profile of PS-NH_2 and PS-CH_3in the direction perpendicular to the TMD surface (z). The profiles are obtained from the time averaging of MD samples for 5 ps after 10 ps equilibration. The profiles clearly indicates that the amine terminal group are strongly physisorbed onto the surface showing a pronounced peak at ~ 2 angstrom, whereas PS-CH_3 does not show any peak near the surface. This suggests that non-functionalized PS simply wanders away from the surface at the urging of thermal and stirring force while PS-NH_2 can be anchored onto the TMD surface forming brush-like molecular clusters. Two simulation snapshots (**Supplementary Figure 3b and c**) demonstrate typical conformational states for PS-NH_2 and PS-CH_3 on the TMD surface.

Simulation Method: The model systems are constructed by generating a chain molecule under interest on a 5x3 rectangular 1T-TiS_2 supercell with toluene molecules, where the density of molecules in the simulation cell is set to be 1.2 g/cm^3 to approximate PS/TiS_2 in toluene. The geometry optimization of the model system was performed according to density functional tight binding (DFTB) theory using DFTB+ package with the tiorg and mio parameter set and UFF-based dispersion corrections [9-14]. The optimized systems were then equilibrated by DFTB-MD simulation in NVT ensemble at 298 K for 10 ps. The concentration profiles were obtained from the time averaging of MD samples for extra 5 ps run after the equilibration.

Supplementary Note 2. Exfoliation and dispersion of TMDs with amine-terminated polymers

- Determination of the optimal conditions for dispersion: TMD to polymer ratio, initial concentration, sonication, and centrifugation time

Initially, the exfoliation of $MoSe_2$ in toluene, which is a poor solvent for $MoSe_2$, was tested with PS, PS-NH_2, and without polymer by tip sonication with a 10-s pulse on and a 5-s pulse off with an amplitude of 50% in an ice bath. $MoSe_2$ in neat toluene and with PS began to precipitate immediately after sonication and completely settled down within few minutes, whereas the dispersion with PS-NH_2 became visually non-scattering with a dark brown color and remained stable, which indicates the preferential attraction between $MoSe_2$ and the amine groups of PS-NH_2. The results prove that PS-NH_2 can enhance the dispersion of the exfoliated nanosheets in non-solvents of $MoSe_2$ (**Supplementary Figure 4a**).

In order to optimize the dispersion conditions, we further investigated the $MoSe_2$ dispersions in detail in terms of the sonication time, initial $MoSe_2$ concentration, and centrifugation rate with 1 mg/mL PS-NH_2 (**Supplementary Figure 4b-f**). The quality of the exfoliation and dispersion was initially determined by the absorbance per unit length, A/l, using UV-vis spectrometry. The optimized dispersion conditions were sonication for 45 min with an initial concentration of 10 mg/mL bulk $MoSe_2$, followed by centrifugation at 1,500 rpm for 30 min. The absorbance spectra of the $MoSe_2$ dispersions obtained under these conditions are shown in Figure 2a. Different from pure PS-NH_2, discernible peaks were observed at 800 and 690 nm, which are similar to the characteristic peaks of exfoliated $MoSe_2$ obtained by other methods without PS-NH_2, confirming the stable exfoliation and dispersion of $MoSe_2$ in toluene [7,8].

- Characterization of $MoSe_2$ dispersion with PS-NH_2: XRD, Raman, XPS and Photoluminescent results

The interaction of amine groups of PS-NH_2 with $MoSe_2$ and the resulting exfoliation into nanosheets was confirmed by X-ray diffraction (XRD) analysis, Raman spectroscopy, and X-ray photoelectron spectroscopy (XPS) and. The XRD patterns of bulk $MoSe_2$ and $MoSe_2$/PS-NH_2 are shown in the **Supplementary Figures 7a and b**. The abrupt decrease and broadening of the (002) reflection in the XRD patterns of $MoSe_2$/PS-NH_2 indicates that $MoSe_2$ was exfoliated into a few layers with different dimensions[15,16]. The Raman spectra of $MoSe_2$/PS-NH_2 deposited on the SiO_2 substrate were compared with the spectra of bulk $MoSe_2$ (**Supplementary Figure 7c**). The exfoliated $MoSe_2$ nanosheets with PS-NH_2 showed characteristic out-of-plane A_{1g} and in-plane E_{2g}^1 Raman modes at 239 and 287.5 cm^{-1}, respectively. These values are consistent with results reported for monolayer $MoSe_2$[15-21]. The observed characteristic out-of-plane A_{1g} mode at 239 cm^{-1}, not be observed in 1T-$MoSe_2$, indicates the 2H polymorph structure of the exfoliated $MoSe_2$ nanosheets. In addition, the peak at about 221 cm^{-1}, associated to a distorted 1T phase, is absent, again indicative of the 2H phase[22,23].

The XPS spectra of the $MoSe_2$/PS-NH_2 confirm the stoichiometry of $MoSe_2$ (**Supplementary Figures 7d-f).** The two characteristic peaks at 228.8 and 232 eV are attributed to the doublet Mo $3d_{5/2}$ and Mo $3d_{3/2}$ binding energies, respectively, for Mo^{4+}. The peaks corresponding to the Se $3d_{5/2}$ and Se $3d_{3/2}$ orbitals of divalent selenide ions (Se^{2-}) are observed at 54.4 and 55.2 eV, respectively. These XPS signals are consistent with values reported elsewhere, corroborating the formation of defect-free $MoSe_2$[16,20]. The N1s spectrum of PS-NH_2 was deconvoluted into three individual peaks, which are assigned to =N– (398.48 eV), –NH– (399.38 eV), and –NH_2 (399.78

eV). These peak positions of the N1s spectrum were shifted after interaction with $MoSe_2$. The binding energies for =N–, –NH–, and $–NH_2$ were shifted to 399.08, 399.78, and 400.48 eV, respectively. The shift of the binding energy of $–NH_2$ is due to the effect of the interaction between the Mo ions and the donating lone pair electrons of $–NH_2$[24,25].

On the other hand, we did not observe the shift of the characteristic peaks of Mo^{4+} corresponding to the doublet Mo $3d_{5/2}$ and Mo $3d_{3/2}$ binding energies because of relatively marginal influence of amine group on the electronic structure of Mo^{4+}. A possible reason of the zero shift of the Mo^{4+} peaks may be due to the low portion of Mo^{4+} atoms bound with amine groups on $MoSe_2$ surface. Since one amine group per polymer chain with its excluded area (S_{ex}) on the $MoSe_2$ surface of approximately 50 nm^2 ($S_{ex} = 2\pi<s>^2$) is supposed to be anchored on a Mo^{4+} atom, the number density of Mo^{4+} atoms occupied by amine terminated polymers is approximately less than 1 % (the weight ratio of PS-NH_2 to $MoSe_2$ in toluene was ~ 0.3 in our process) which may rarely affect the peak positions. In addition, no peak shift of both Mo and Se bands was observed due to the transition from 2H to 1T phase. The results imply that the TMDs did not undergo any considerable structural changes upon exfoliation, and in fact retain the semiconducting nature and 2H polymorph of their starting bulk counterparts. The results we obtained with $MoSe_2$ nanosheets modified with PS-NH_2 were similarly observed with the nanocomposites of MoS_2 with PS-NH_2 as shown in (**Supplementary Figure 9)**.

- Effect of the molecular weight of PS-NH_2 on $MoSe_2$ dispersion

The effect of the molecular weight of PS-NH_2 on the $MoSe_2$ exfoliation was also investigated with four different molecular weights (9.5 k, 25 k, 40 k, and 108 k g mol^{-1}) of PS-NH_2. The initial concentration of PS-NH_2 was kept constant at 1 mg/mL and the final concentration of exfoliated $MoSe_2$ was measured (**Supplementary Figure 10**). As expected, the effectiveness of PS-NH_2 in exfoliation decreases with increasing molecular weight of PS-NH_2. The steric

hindrance resulting from the entangled conformations of high molecular polymer chains decreases the reactivity of amine groups and restricts the mobility of the polymer chains in the solvents. Therefore, the lower the molecular weight of PS-NH_2, the more favorable the amine interactions and thus, better exfoliation and dispersion of $MoSe_2$ nanosheets occurs. PS-NH_2 with a molecular weight of 9.5 k was further utilized for all of the analyses.

- Determination of the concentration of $MoSe_2$ dispersed with PS-NH_2

The concentration of the dispersed $MoSe_2$ nanosheets with PS-NH_2 in toluene was determined by a traditional gravimetric method[8]. A precisely measured volume of the $MoSe_2$ dispersion was poured into a crucible and the solvent was removed in a vacuum oven overnight. Then, the mass of the crucible was measured and used to determine the concentration of $MoSe_2$ in the dispersion according to the following equation (**1**).

$$Concentration = \frac{\text{Final crucible mass} - \text{Initial crucible mass (mg)}}{\text{Volume of the solvent (mL)}} \quad \text{..............................} \ (\mathbf{1})$$

TGA was utilized to determine the dispersed amount of $MoSe_2$ nanosheets in the PS-NH_2-modified $MoSe_2$ composite (**Supplementary Figure 11**). From the TGA results of the PS-NH_2-modified $MoSe_2$ composite, the quantity of the $MoSe_2$ nanosheets was estimated to be 26%, which allowed us to determine the final concentration of the $MoSe_2$ nanosheets in the dispersion without PS-NH_2 to be 0.22 mg/mL.

The extinction coefficient (∝) of the $MoSe_2$ nanosheets with PS-NH_2 in various organic solvents was determined using the Lambert–Beer law (equation **2** and **3**).

$$\boldsymbol{A} = \propto \boldsymbol{lc} \quad \text{....................................} \ (\mathbf{2})$$

$$\propto = A/lc \quad \text{..................................} \quad \mathbf{(3)}$$

Here, *A* is the measured absorbance, *l* is the cell length, and *c* is the dispersed concentration.

The samples were controllably diluted in series and the absorbance per unit length (*A/l*) as a function of the $MoSe_2$ concentration was measured. The background scattering illustrated by the line in the plot of the absorbance spectrum of $MoSe_2$ with PS-NH_2 (**Supplementary Figure 11b**) was subtracted from the spectrum[8]. The background subtracted *A/l* value at 800 nm was plotted as a function of the $MoSe_2$ concentration (**Figure 2c**) and the *A/l* values linearly increased with increasing measured concentration. The calculated absorption coefficient (α) was determined from the slope of the curve to be **932 L g^{-1} m^{-1}**, which was used to determine the concentrations of the further dispersions.

- $MoSe_2$ dispersed with PS-NH_2 in various solvent media

The absorbance variations of the $MoSe_2$ dispersions were measured at an excitation wavelength of 800 nm with 1 mg/mL PS-NH_2 as a function of the $MoSe_2$ concentration in each solvent (**Supplementary Figure 12**). The absorbance linearly increased with the amount of $MoSe_2$ in accordance with Lambert-Beer behavior and the results imply that the $MoSe_2$ nanosheets are uniformly dispersed without aggregation in all of the solvents.

We further investigated important dispersion properties such as the maximum amount of $MoSe_2$ dispersed in a solvent and the amount of PS-NH_2 required for $MoSe_2$ dispersion at a certain concentration dependent upon the solvent (**Supplementary Figure 13**). The dispersed $MoSe_2$ concentration increased monotonically with increasing PS-NH_2 concentration due to the increase of reactive amine functional groups.

- Stability of $MoSe_2$ dispersed with PS-NH_2

The stability of the $MoSe_2$ dispersions after centrifugation in solvents with 5 mg/mL PS-NH_2 was monitored by optical absorbance at 800 nm as a function of time, as shown in **Supplementary Figure 14**. The absorbance of the $MoSe_2$ dispersions did not significantly change, indicating that the dispersed $MoSe_2$ was highly stable without re-aggregation and sedimentation for a period of more than 3 weeks. The high-concentration dispersion and long-term stability of $MoSe_2$ can be ascribed to the strong interaction of the amine groups with the $MoSe_2$ nanosheets as well as the long and flexible chains of polymers, which provide good solubility in various solvents.

The large amount of PS-NH_2 in the PS-NH_2-modified $MoSe_2$ composite may not be suitable for subsequent electronic applications due to the intrinsic insulating properties of PS-NH_2. To remove the excess amount of the non-interacted PS-NH_2 in the dispersions, the dispersions were centrifuged for 90 min at 15,000 rpm. The precipitate was collected and dried to remove the solvents. The resultant powder contains approximately 85% $MoSe_2$ (**Supplementary Figure 11a** (**iv**)), which was further utilized to fabricate the photodetectors.

Supplementary Note 3. Photo-detection performance of thin TMD composite films with end-functionalized polymers

- Calculation of photo-detection performance

The photodetector performance[26,27] including the responsivity, specific detectivity and external quantum efficiency values are calculated as follows (equations **4,5** and **6**),

1. Responsivity (R)

$R = \Delta I/(PA)$ ………………………..………… (**4**)
$\Delta I = I_{light} - I_{dark}$
P = *Light intensity*
A = *Active area of the device*

2. Specific Detectivity (D)*

$D^* = R \cdot A^{1/2} / (2qI_d)^{1/2}$ ……………………..…… (**5**)

R = *Responsivity*

A = *Active area of the device*

q = *Absolute value of electron charge*

(1.6 × 10−19 Coulombs)

I_d = *Dark current*

3. Quantum Efficiency (EQE)

$EQE = hcR/(e\lambda)$ ……………………..………… (**6**)

h = *Planck's constant*

c = *Velocity of light*

e = *Electronic charge*

λ = *Incident light wavelength*

- Origins of the photo-current arising from TMD nanosheets exfoliated with PS-NH_2

The photo-conduction of thin TMD composite films with end-functionalized polymer upon NIR exposure arises from photo-induced band excited carriers that can be dissociated into free electrons and holes thermally or by a large electric field. The excellent photo-detecting

performance of our composite is mainly attributed to 3-dimensionally interconnected $MoSe_2$ nanosheets with their preferred in-plane orientation between two electrodes. This alignment of nanosheets primarily provides numerous conduction pathways of free carriers produced by exciton dissociation. Furthermore, we believe that electrically insulating PS-NH_2 anchored on the $MoSe_2$ surface in between two sheets played an important role in enhancing the detection performance. The thermally insulating PS chains partially covering the surface of nanosheets may promote the exciton dissociation due to a local temperature increase of $MoSe_2$, leading to sufficient thermal energy for the dissociation. In addition, the $MoSe_2$ nanosheets partially covered with electrically insulating PS chains can act as a potential barrier to inter-nanosheet hopping. This localized electric field at the sheet-to-sheet interface can amplify the exciton dissociation, giving rise to the high photocurrent observed.

Supplementary Note 4. Tunable photo-detection of thin TMD/PS-NH_2 composite films containing solution-blended MoS_2 and $MoSe_2$ nanosheets

- Homogeneous mixing of MoS_2 and $MoSe_2$ nanosheets with PS-NH_2

Co-dispersion of MoS_2 and $MoSe_2$ nanosheets in toluene was successfully performed with PS-NH_2, as shown in **Supplementary Figure 28.** In particular, characteristic absorbance peaks arising from both MoS_2 and $MoSe_2$ nanosheets are apparent. Homogeneous mixing of the two TMD nanosheets was evidenced by the results in which the characteristic absorbance of $MoSe_2$ nanosheets at 800 nm almost linearly increases with $MoSe_2$ in a blended dispersion, as shown in **Supplementary Figure 28c**. Energy dispersive X-ray spectroscopy (EDX) is a useful tool to confirm the homogenous mixing of the two TMD nanosheets in PS-NH_2. Selective mapping of the constituent atoms of MoS_2 and $MoSe_2$ (Mo, S, and Se) clearly suggests that the atoms are distributed throughout the sample and the relative intensities of the atoms vary upon mixing of the two TMDs with PS-NH_2. For instance, Se becomes strong with the amount of $MoSe_2$ in a blended composite, as shown in **Supplementary Figure 29**. Quantitative analysis of the relative amount of the atoms was performed, as shown in **Supplementary Figure 30,** where the results again confirm the capability of our method to control the amount of two or more different TMDs in a polymer composite.

- Experimental setup for absorption and photocurrent spectra

The absorption and photocurrent spectra measurement were performed at high selectivity system. For the wavelength-selective experiments, the detailed experiment setup is shown in **Supplementary Figure 31**. Instead of sending the broadband light and measuring the absorbance, we send the supercontinuum source (available wavelength from 450 to 2400 nm) into the monochromator (± 0.2 nm accuracy, 1.6 nm/mm dispersion), through which we "pre-selected" a narrow spectrum (bandwidth ~1 nm). The absorption spectra were obtained at each selected wavelength by scanning the grating of the monochromator (grove = 150, 500 nm blaze).

Supplementary Note 5. Origin of the photocurrent of blended MoS_2 and $MoSe_2$ nanosheets with PS-NH_2: photo-induced, carrier-relaxation dynamics

- Ultrafast optical-pump and optical-probe spectroscopy

In order to measure the exciton formation and recombination kinetics of the solution-blended mixture of $MoSe_2$ and MoS_2 composite film, we performed ultrafast optical-pump and optical-probe spectroscopy. **Supplementary Figure 35** shows the schematic of the system used in the pump-probe measurements. We used a 250-kHz Ti:sapphire regenerative amplifier (Coherent RegA 9050) to generate ultrashort laser pulses with a 50-fs pulse width and an 800-nm center wavelength. The RegA output pulse was separated into two parts: one for pump excitation and one for the probe of interband-like optical transition. For the interband pump, we converted the 800-nm pulse to a 400-nm pulse via second harmonic generation in a 1-mm thick beta barium borate (BBO) crystal. The other part of the amplifier output was focused on a 1-mm thick sapphire disk to create white-light supercontinuum, which was used as a probe pulse. Both the pump and probe pulses are focused on the TMD/polymer composite film and the transmitted probe pulse was detected by a near-infrared Si photodetector after passing through a monochromator (Newport 74125). The pump pulse was mechanically chopped at 10 kHz before the sample and a lock-in amplifier (Standard Research Systems SR850) recorded the pump-induced probe-transmission changes.

- Photoconductive gain estimation using data from ultrafast experiments

One essential feature of our photo detector based on the blended MoS_2 and $MoSe_2$ nanosheets is that the direct gap nature of TMDs is fully exploited, rather than just simply mixing the two “bulk-like” TMD powders. Although XRD, Raman, XPS and FT-IR experiments may show the intrinsic properties of exfoliated nanosheets, the photo-induced ultrafast carrier relaxation dynamics can provide more direct evidence in distinguishing the bulk and few-layered TMD nanosheets than the above measurements. In addition, given that the external quantum efficiency of most TMD-based photo detector is very poor, in the range of 0.001 or less, our

device shows extremely large, ~ 10^3 (**Figure 3e**), with 19.5 of photoconductive gain G at 238.8 mW/cm^2 incident power density. The photoconductive gain G was estimated by the following way: The photocurrent ΔI is given by following equation: $\Delta I = qPAG\lambda/hc$, where PA is the total power injected on the active channel, q is the electron charge, λ is the wavelength of incident light, h is the Planck's constant, and c is the speed of light. Based on our experimental results, we have extracted G of 19.5 with ΔI of 400 mA at the drain voltage of +10 V. Ultrafast measurement also provides the information of the photoconductive gain ($=\tau_{nr}/t_{tr}$), where τ_{nr} is the nonradiative lifetime and τ_{tr} is the transit time. We extracted τ_{nr} to be approximately 500 ns for G of 19.5 with the calculated τ_{tr} ($=L^2/\mu V_{DS}$, where L is the channel length, μ is the mobility, and V_{DS} is the drain voltage). The L and V_{DS} of the channels are 50 μm and + 10 V respectively, and we applied the effective mobility of μ ~ 100 cm^2/Vs [28]. Prior investigations revealed that the nonradiative lifetime τ_{nr} of TMDs consists of multiple exponential components[29]. Some useful information can be obtained from the ultrafast measurement combined with the G calculated from photo-detection properties of our devices.

Next, the detailed nonradiative recombination components can be found by fitting the OP-OP data. We re-plotted the dynamics of $MoSe_2$ in the **Supplementary Figure 36**. The carrier relaxation dynamics involves two clear decay components and one nearly step-function-like response (limited by our temporal window). The lifetimes for each component are extracted as τ_1 = 2.1 ± 0.1 ps and τ_2 = 16.6 ± 2.0 ps. As demonstrated in prior investigations [29-31], we assign each lifetime to the following relaxation channels: the fastest τ_1 is attributed to the carrier trap to the interfacial defects, τ_2 is the electron-phonon scattering. It should be, however, noted that there exist many nonradiative relaxation channels in TMD, and the origin of each of these

mechanisms are still under active research. This is particularly true in our TMD/polymer composites due to the added ligands in our TMD devices. It is indeed very challenging for us to exactly identify the effect of ligands on the nonradiative dynamics. However, the fact that the interband absorption response is similar between the TMD/polymer composite and the bare TMD nanosheet provides us some clue that the effect of ligands may be marginal. Nevertheless, given that the fast relaxation time constants of τ_1 and τ_2 are similar to that of most TMD materials, we attributed τ_1 and τ_2 to the defect and electron-phonon scattering, respectively.

If we use $\tau_{nr} \approx \tau_1$ or τ_2, then G does not match with the extracted G–this can be well understood because both τ_1 (which is related to the defect) and τ_2 (which is electron-phonon scattering) do not contribute to the photo-carrier transport, i.e. both trapped and inelastically scattered carriers do not contribute to G. The extracted G of 20 matches well when we use $\tau_{nr} \approx$ 500 ns, which is almost a step function in our pump-probe transients. Because of the limited pump-probe delay (~ 250 ps), our pump-probe measurement could not exactly determine the longest time-constant, but we can only infer the long nonradiative lifetime only in an indirect way, speculating in the range of a few hundreds of ns. Our pump-probe experiments combined with the calculation of photocoductive gain suggest at least that the fast relaxation components can be excluded to explain the photo-response behavior we observed in our devices.

Supplementary Note 6. Ultrafast optical-pump THz-probe spectroscopy

Ultrafast optical-pump and THz-probe spectroscopy was performed to investigate the photo generated intraband carrier relaxation dynamics of few-layer MoS_2 nanosheets exfoliated by PS-NH_2. The experimental setup for the optical-pump THz-probe measurement is shown in

Supplementary Figure 37. For the pump (400 nm center wavelength), we employed the same configuration used in the optical pump-probe spectroscopy analysis. For the THz probe, a broadband THz pulse covering the frequency range of 0.7–2.7 THz was generated by optical rectification in a 0.5-mm-thick (110) oriented ZnTe crystal. To detect the THz pulse, we used conventional electro-optic sampling using the same pair of ZnTe crystals. For the electro-optic sampling, a Wollaston prism splits the elliptically polarized 800-nm sampling pulse into two orthogonal components. A pair of Si-balanced detectors measures the intensity difference between the two, which is proportional to the incident THz-field amplitude. **Supplementary Figure 38** shows the measured electro-optic signal as a function of the THz-field delay (electro-optic sampling delay), in which the black line is the reference THz field (THz probe without pump) and the red line is the pump-induced THz-field change (ΔE) when the delay between the 400-nm pump and THz-probe pulse is overlapped (i.e. pump-probe delay Δt = 0 ps). We note that the two electro-optic fields are out-of-phase, indicating that the increased THz conductivity is due to pump excitation. For the time-resolved dynamics shown in **Figure 5f** (main text), the data were taken when the THz-field delay was zero.

Supplementary References

1. Becke, A. D. Density-functional thermochemistry. III. The role of exact exchange. *J. Chem. Phys.* **98**, 5648–5652 (1993).

2. Hay, P. J. & Wadt, W. R. *Ab initio* effective core potentials for molecular calculations. Potentials for K to Au including the outermost core orbitals. *J. Chem. Phys.* **82**, 299 (1985).

3. Frisch, M. J. *et al*. GAUSSIAN 09 (Revision D.01), Gaussian, Inc., Wallingford CT, 2004.

4. Mennucci, B., Cances, E., & Tomasi, J. Evaluation of Solvent Effects in Isotropic and Anisotropic Dielectrics and in Ionic Solutions with a Unified Integral Equation Method: Theoretical Bases, Computational Implementation, and Numerical Applications. *J. Phys. Chem. B* **101**, 10506-10517 (1997).

5. Cances, E., Mennucci, B., & Tomasi, J. A new integral equation formalism for the polarizable continuum model: Theoretical background and applications to isotropic and anisotropic dielectrics. *J. Chem. Phys.* **107**, 3032 (1997).

6. Tomasi, J., Mennucci, B., & Cammi, R. Quantum mechanical continuum solvation models. *Chem. Rev.* **105**, 2999-3094 (2005).

7. Wilson, J. A. & Yoffe, A. D. Transition metal dichalcogenides: discussion and interpretation of observed optical, electrical and structural properties. *Adv. Phys.* **18**, 193–335 (1969).

8. Coleman, J. N. *et al.* Two-dimensional nanosheets produced by liquid exfoliation of layered materials. *Science* **331**, 568–571 (2011).

9. Aradi, B., Hourahine, B. & Frauenheim, T. DFTB+, a sparse matrix-based implementation of the DFTB method. *J. Phys. Chem. A* **111**, 5678–5684 (2007).

10. Porezag, D., Frauenheim, T., Köhler, T., Seifert, G., & Kaschner, R. Construction of tight-binding-like potentials on the basis of density-functional theory: Application to carbon. *Phys. Rev. B* **51**, 12947 (1995).

11. Seifert, G., Porezag, D., Frauenheim. Calculations of molecules, clusters, and solids with a simplified LCAO-DFT-LDA scheme. Int. *J. Quant. Chem.* **58,** 185-192 (1996).

12. M. Elstner, M. *et al*. Hydrogen bonding and stacking interactions of nucleic acid base pairs: A density-functional-theory treatment. *J. Chem. Phys.* **114** , 5149-5155 (2001).

13. Zhechkov, L. *et al*. An efficient *a posteriori* treatment for dispersion interaction in density-functional-based tight binding. *J. Chem. Theory Comput.* **1**, 841-847 ***(***2005).

14. Dolgonos, G., Aradi, B., Moreira, N. H. & Frauenheim, T. An Improved Self-Consistent-Charge Density-Functional Tight-Binding (SCC-DFTB) Set of Parameters for Simulation of Bulk and Molecular Systems Involving Titanium. *J. Chem. Theory Comput.***6**, 266-278 ***(***2010).

15. Matte, H. S. S. R., Plowman, B., Datta. R, & Rao, C. N. R. Graphene analogues of layered metal selenides. Dalton Trans. **40**, 10322–10325 (2011).

16. Zhou, X. *et al.* Fast colloidal synthesis of scalable Mo-rich hierarchical ultrathin $MoSe_{2-x}$ nanosheets for high-performance hydrogen evolution. *Nanoscale* **6**, 11046-11051 (2014).

17. Tonndorf, P. *et al*. Photoluminescence emission and raman response of monolayer MoS_2, $MoSe_2$, and WSe_2. *Opt. Exp.* **21**, 4908–4916 (2013).

18. Shaw, J. C. *et al*. Chemical vapor deposition growth of monolayer $MoSe_2$ nanosheets. *Nano Res.* **7**, 1-7 (2014).

19. Tongay, S. *et al*. Thermally driven crossover from indirect toward direct bandgap in 2D semiconductors: $MoSe_2$ *versus* MoS_2. *Nano Lett.* **12**,5576-5580 (2012).

20. Chang, *Y-H. et al.* Monolayer $MoSe_2$ grown by chemical vapor deposition for fast photodetection. *ACS Nano* **8**,8582-8590 (2014).

21. Xia, J. *et al*. CVD synthesis of large-area, highly crystalline $MoSe_2$ atomic layers on diverse substrates and application to photodetectors. *Nanoscale* **6**, 8949-8955 (2014).

22. Gupta, U. *et al*. *et al*. Characterization of few-layer 1T-$MoSe_2$ and its superior performance in the visible-light induced hydrogen evolution reaction. *APL Mat.* **2**, 092802 (2014).

23. Guardia, L. Production of aqueous dispersions of inorganic graphene analogues by

exfoliation and stabilization with non-ionic surfactants. *RSC Adv.***4**, 14115-14127 (2014).

24. Kuo, P.L. *et al*. Stabilizing effect of pseudo-dendritic polyethylenimine on platinum nanoparticles supported on carbon. *J. Phys. Chem. B* **110**, 3071–3077 (2006).

25. Li, J. et al. Enhanced-electrocatalytic activity of $Ni_{1-x}Fe_x$ alloy supported on polyethyleneimine functionalized MoS_2 nanosheets for hydrazine oxidation. *RSC Adv.* **4**, 1988-1995 (2014).

26. Gong, X. *et al*. High-detectivity polymer photodetectors with spectral response from 300 nm to 1450 nm. *Science* **325**, 1665-1667 (2009).

27. Fang, X. *et al*. New ultraviolet photodetector based on individual Nb_2O_5 nanobelts. *Adv. Funct. Mater.* **21**, 3907–3915 (2011).

28. Kim, S. *et al*. High-mobility and low-power thin-film transistors based on multilayer MoS_2 crystals. *Nat*. *Comm*.**3**,1011 (2012).

29. Shi, H. *et al*. Exciton dynamics in suspended monolayer and few-layer MoS_2 2D crystals. *ACS nano* **7**, 1072-1080 (2013).

30. Wang, Q. *et al*. Valley carrier dynamics in monolayer molybdenum disulfide from helicity-resolved ultrafast pump–probe spectroscopy. *ACS nano* **7**, 11087-11093 (2013).

31. Cui, Q, Ceballos, F., Kumar, N. & Zhao, H. Transient Absorption Microscopy of Monolayer and Bulk WSe_2. *ACS nano* **8**, 2970-2976 (2014).
